# Supplementary material for: CcrZ is a pneumococcal spatiotemporal cell cycle regulator that interacts with FtsZ and controls DNA replication by modulating the activity of DnaA
Source: Nat Microbiol. 2021 Aug 9;6(9):1175–87. doi: 10.1038/s41564-021-00949-1 (PMC8387234; doi:10.1038/s41564-021-00949-1)
Supplement: Supplementary file 1 — Legends to Supplementary Videos 1–8, Methods, Tables 1–4 and references. [file 41564_2021_949_MOESM1_ESM.pdf]

nature portfolio

https://doi.org/10.1038/s41564-021-00949-1

Supplementary information

CcrZ is a pneumococcal spatiotemporal cell cycle regulator that interacts with FtsZ and controls DNA replication by modulating the activity of DnaA

In the format provided by the authors and unedited

Supplementary Information

CcrZ is a pneumococcal spatio-temporal cell cycle regulator that interacts with FtsZ and controls DNA replication by modulating the activity of DnaA

Clement Gallay, Stefano Sanselicio, Mary E. Anderson, Young Min Soh, Xue Liu, Gro A. Stamsås, Simone Pelliciari, Renske van Raaphorst, Julien Dénéréaz, Morten Kjos, Heath Murray, Stephan Gruber, Alan D. Grossman, Jan-Willem Veening

Contents:

Supplementary Videos legends
1. 3D-SIM of GFP-CcrZ in wild type cells.
2. Time lapse microscopy of GFP-CcrZ in live cells.
3. Time lapse analysis of GFP-CcrZ and FtsZ-mCherry in live cells.
4. 3D-SIM of GFP-CcrZ and FtsZ-mCherry in wild type cells.
5. Time lapse microscopy of CcrZ-mKate2 in FtsZ depleted cells.
6. Time lapse analysis of HlpA-mKate2 in Δ*ccrZ* cells.
7. Time lapse microscopy of FtsZ-mTurquoise2 in $DnaA^{TS}$ following temperature shift.
8. Time lapse analysis of CcrZ-mKate2, $DnaN\text{-}sfTQ^{OX}$ and $ParB_p$-sfmYFP in live cells.

Supplementary Methods: Strains construction

Supplementary Tables 1-4
1. Strains and plasmids used in this study
2. Oligonucleotides used in this study
3. Ratio of spectral counts between GFP-CcrZ and GFP from LC-MS/MS
4. CRISPRi-seq results for *ccrZ*-complementation vs *ccrZ*-depletion

Supplementary References

Supplementary Video 1: 3D-SIM of GFP-CcrZ in wild type cells.
Volume projection of 240 reconstructed 3D-SIM images from a chain of four live *S. pneumoniae* cells expressing msfGFP-CcrZ shows CcrZ forming patchy rings.

Supplementary Video 2: Time lapse microscopy of GFP-CcrZ in live cells.
Localization overtime of msfGFP-CcrZ (green) at 30°C overlaid with phase contrast (gray) shows that CcrZ localizes exclusively at the division site overtime. Time interval: 10 min. Scale bar: 3 µm.

Supplementary Video 3: Time lapse analysis of GFP-CcrZ and FtsZ-mCherry in live cells.
Localization of msfGFP-CcrZ (green) and FtsZ-mCherry (red) overlaid with phase contrast (gray) at 30°C shows that CcrZ and FtsZ co-localize overtime. Time interval: 10 min. Scale bar: 3 µm.

Supplementary Video 4: 3D-SIM of GFP-CcrZ and FtsZ-mCherry in wild type cells.
Volume projection of 240 reconstructed 3D-SIM images of a chain of four *S. pneumoniae* cells shows that msfGFP-CcrZ (green) and FtsZ-mCherry (red) form a similar ring structure and co-localize.

Supplementary Video 5: Time lapse microscopy of CcrZ-mKate2 in FtsZ depleted cells.
Depletion of FtsZ overtime shows a rapid spread of CcrZ-mKate2 (red) signal in the cytoplasm. Cells are visualized by phase contrast (grey). Time interval: 10 min. Scale bar: 3 µm.

Supplementary Video 6: Time lapse analysis of HlpA-mKate2 in Δ*ccrZ* cells.
Localization overtime of HlpA-mKate2 (red) used as a chromosomal marker in live *ccrZ*-deleted cells shows absence of nucleus and "guillotined" chromosome in several cells visualized by phase contrast (grey). Time interval: 5 min. Scale bar: 3 µm.

Supplementary Video 7: Time lapse microscopy of FtsZ-mTurquoise2 in DnaA^{TS} following temperature shift.
Localization overtime of FtsZ-mTurquoise2 (cyan) in DnaA^{TS} when cells are grown at 40°C following a pre-incubation at 30°C shows a delocalization of FtsZ after roughly four to five generations. Cells are visualized by phase contrast (grey). Time interval: 15 min. Scale bar: 3 µm.

Supplementary Video 8: Time lapse analysis of CcrZ-mKate2, DnaN-sfTQ^{OX} and ParB$_p$-sfmYFP in live cells.
Localization overtime of CcrZ-mKate2 (red, marked by red arrows), DnaN-sfTQ^{OX} (cyan, marked by cyan arrows) and ParB$_p$-sfmYFP (yellow, marked by yellow arrows) overlaid with phase contrast (grey) shows that the origin of replication is first brought to the future division site, while the replication machinery localizes at mid-cell with CcrZ, until CcrZ re-localizes to the new division site to start a new round of replication. Time interval: 2 min 30 sec. Scale bar: 1.5 µm.

Supplementary Methods

Strains constructions

All strains and plasmids used for constructions are listed in Supplementary Table 1 and all primers are listed in Supplementary Table 2.

All *S. pneumoniae* strains were constructed by integrating into the chromosome by double homologous recombination either circular plasmids or linear DNA fragments possessing two ~1 kb region homology assembled using standard restriction-ligation, PCR assembly, Gibson assembly or Golden Gate assembly. In all cases parental strains were transformed directly with the assembled products. Constructs were confirmed by PCR and the resulting fragment sequenced.

P_{Zn}-*ccrZ*$^{+/+}$ and *ccrZ*$^{+/+}$. For insertion of a second copy of *spv_0476* (*ccrZ*) under control of either P_{Zn} or P_{lac}, *spv_0476* with its native RBS was amplified by PCR from D39V genome with primers 1/2 or 1/9 respectively and cloned into pMK11 vector[1] between EcoRI-SpeI restriction sites allowing for the genetic fusion P_{Zn}-*spv_0476* to be inserted in place of *bgaA* (coding for β-galactosidase) locus, or cloned into pPEPY[2] between EcoRI-BamHI sites to obtain the genetic fusion P_{lac}-*spv_0476* for insertion in place of the *cil* locus (chromosomal integration locus – disrupting the non-coding *spv_2165* gene). pMK11-*spv_0476* was then transformed into D39V wild type and pPEPY-*spv_0476* into D39V *lacI*[3] as this strains expresses constitutively the repressor LacI.

P_{Zn}-*ccrZ*$^{-/+}$ and *ccrZ*$^{-/+}$. For deletions of *spv_0476,* both flanking regions of the gene were amplified by PCR on D39V genome with primers 3/4 and 5/6 and assembled using Gibson assembly[4] to an erythromycin resistant marker amplified from a strain *ftsZ-mKate2*-ery[1] with primers 7/8. The final product was transformed into P_{Zn}-*ccrZ*$^{+/+}$ strain in presence of $ZnCl_2$ in order to express the extra copy of *ccrZ*. The resulting *ccrZ*::*ery* product together with the flanking regions was amplified from the P_{Zn}-*ccrZ*$^{-/+}$ genome with primers 10/11 and transformed into *ccrZ*$^{+/+}$ in presence of IPTG.

ftsZ-spc and *ftsZ-mTurquoise2*. The spectinomycin resistance marker s*pc* was amplified by PCR from was plasmid pPEP23[5] with primers 16/17, *ftsZ* and flanking regions were amplified on D39V genome with primers 12/13 and 14/15. All three fragments were assembled using Golden Gate assembly with BsmBI[6] and the resulting product transformed into D39V wild type. *mTurquoise2* was amplified from vector mTurquoise2-pBAD (addgene) with primers 19/20, *ftsZ-linker* was amplified from *ftsZ-mKate2* strain[1] with primers 12/18 and downstream flanking region of *ftsZ* with *spc* amplified on *ftsZ-spc* genome with primers 15/21. All three fragments were assembled using Golgen Gate assembly (BsmBI) and transformed into D39V wild type in order to replace the native *ftsZ* with *ftsZ-mTurquoise2-ery* fusion.

ccrZ$^{-/+}$ *ftsZ-mTurquoise2* and *dnaA*^{TS} *mTurquoise2*. *ftsZ-mTurquoise2* genetic fusion and flanking regions were amplified by PCR with primers 12/15 from *ftsZ-mTurquoise2* strain and inserted either into *ccrZ*$^{-/+}$ strain in presence of IPTG to keep high levels of CcrZ (*ccrZ*$^{-/+}$ *ftsZ-mTurquoise2*) or into the thermosensitive *dnaA* mutant *dnaA*^{TS} [7] (*dnaA*^{TS} *mTurquoise2*).

ccrZ$^{-/+}$ Δ*trmB*. For co-deletion of *ccrZ* and *trmB* (same operon and overlapping), upstream region of *ccrZ* was amplified by PCR on D39V genome with primers 22/23, erythromycin resistance marker was

amplified with primers 24/25 on *ccrZ*$^{-/+}$ genome and downstream region of *trmB* was amplified on D39V genome with primers 26/27. All three fragments were assembled using Golden Gate (BsaI) and inserted into strain *ccrZ*$^{-/+}$ in presence of IPTG.

P_{Zn}-ccrZ-gfp, *P_{Zn}-ftsZ-gfp, P_{Zn}-gfp-ccrZ*, R6 *P_{Zn}-gfp-ccrZ* and TIGR4 *P_{Zn}-gfp-ccrZ*. For translational fusion of monomeric superfolder green fluorescent protein (msfGFP) at the C-terminal extremities of CcrZ and FtsZ, *ccrZ* and *ftsZ* together with their respective RBS were amplified by PCR on D39V genome with primers 28/29 and 30/31, respectively, and cloned into plasmid pMK17[1] between restriction sites NotI-SpeI, allowing for insertion of fusion *ccrZ-gfp* or *ftsZ-gfp* under control of P_{Zn} at the *bgaA* locus. The final product was then transformed into D39V wild type, R6[8] and TIGR4[9]. For translational fusion of msfGFP at the N-terminal extremity of CcrZ (as *ccrZ* 3' region overlaps with *trmB* 5' region), *ccrZ* excluding the START codon and including STOP codon was first amplified by PCR from D39V genome with primers 32/33 and cloned into pCG6[3] between restriction sites SpeI-NotI, allowing for genetic fusion of *msfgfp* to the 5' of *ccrZ* under control of P_{Zn}. pCG6 derivates from pMK17 and can therefore integrates into the *bgaA* locus. D39V wild type was then transformed with the final product.

gfp-ccrZ and *gfp-ccrZ ftsZ-mCherry*. *gfp-ccrZ* fragment was amplified by PCR from *P_{Zn}-gfp-ccrZ* strain with primers 37/38, upstream flanking region was amplified with primers 34/35 from D39V genome and a kanamycin marker was amplified with primers 36/39 from pPEP2K1[10]. All three fragments were assembled by Gibson assembly and transformed into D39V wild type resulting in strain *gfp-ccrZ*, with *gfp-ccrZ* fusion in place of native *ccrZ* and under control of the native P_{ccrZ} promoter. For co-localization of CcrZ with FtsZ, *ftsZ-mCherry* was amplified by PCR with primers 12/15 from *ftsZ-mCherry* strain[1] and transformed into *gfp-ccrZ* strain.

ftsZ$^{+/+}$ and *ftsZ*$^{-/+}$. For insertion of an inducible ectopic version of *ftsZ*, *ftsZ* gene was amplified from D39V genome by PCR with primers 40/41 and cloned into vector pPEPY between EcoRI and BamHI restriction sites. D39V *lacI* was then transformed with the ligated product, leading to insertion of P_{lac}-*ftsZ* in the *cil* locus. Spectinomycin marker together with *ftsZ* downstream region were amplified with primers 15/42 from strain *ftsZ-spc* and the region upstream of *ftsZ* START codon was amplified by PCR from D39V genome with primers 43/44. Both fragments were assembled using Golden Gate assembly (BsmBI) and the strain *ftsZ*$^{+/+}$ was transformed with the resulting product in presence of IPTG, to keep a high level of FtsZ, leading to the depletion strain *ftsZ*$^{-/+}$.

ccrZ-mKate2 and *ftsZ*$^{-/+}$ *ccrZ-mKate2*. For genetic fusion of *ccrZ* with the fluorescent protein mKate2, *ccrZ* upstream and downstream regions were amplified by PCR with primers 45/46 and 49/50, respectively, from D39V genome and *mKate2-ery* (with linker) was amplified with primers 47/48 from *ftsZ-mKate2* strain. All three fragments were assembled by Golden Gate assembly (BsmBI) and the product transformed into D39V wild type. *ccrZ-mKate2* was then amplified by PCR from the previously created strain with primers 50/51 and the product transformed into *ftsZ*$^{-/+}$ strain in presence of IPTG to keep a high level of FtsZ.

ccrZ-LgBit. In order to fuse *ccrZ* to *LgBit* sequence in place of the native *ccrZ*, upstream region of *ccrZ* was amplified by PCR with primers 52/53 and the downstream region together with erythromycin marker was amplified with primers 54/55 on strain *ccrZ-mKate2*. A gBlocks fragment (Integrated DNA

Technologies) containing the *LgBit* sequence flanked with two BsaI sites was synthesised. Both PCR fragments and gBlocks were assembled using Golden Gate assembly (BsaI) and inserted into D39V wild type by transformation.

ftsZ-SmBit and *ccrZ-LgBit ftsZ-SmBit*. For fusion of *ftsZ* with *SmBit* sequence, *SmBit* sequence was synthesized as a gBlocks fragment (Integrated DNA Technologies) and amplified by PCR with primers 56/57. *ftsZ* with its upstream region sequence were amplified with primers 58/59 on D39V genome and the downstream region with spectinomycin marker were amplified with primers 60/61 on strain *ftsZ-spc*. All three fragments were then assembled with Golden Gate assembly (BsmBI) and transformed into D39V wild type and *ccrZ-LgBit* strains.

ccrZ-SmBit and *ccrZ-LgBit ccrZ-SmBit*. For *ccrZ-SmBit* construction, *ccrZ* upstream and downstream regions were amplified by PCR with primers 62/63 and 66/67, respectively, on D39V gDNA and SmBit was amplified with primers 64/65 from strain *ftsZ-SmBit*. All three fragments were assembled with Golden Gate assembly (BsmBI) and the product was transformed into strain D39V wild type. *ccrZ-SmBit* was then amplified by PCR with primers 70/67 from the resulting strain and *ccrZ-LgBit*, with upstream region was amplified with primers 68/69 from strain *ccrZ-LgBit*. The two fragments obtained were assembled by Golden Gate assembly (BsmBI) and transformed into D39V wild type.

ccrZ-LgBit cps2E-SmBit, ccrZ-LgBit hlpA-SmBit, ccrZ-LgBit dnaA-SmBit, ccrZ-LgBit ezrA-SmBit, ccrZ-LgBit ftsA-SmBit, ccrZ-LgBit ftsH-SmBit, ccrZ-LgBit ftsW-SmBit, ccrZ-LgBit pepN-SmBit, ccrZ-LgBit pbp2x-SmBit, ccrZ-LgBit zapA-SmBit, ccrZ-LgBit fruA-SmBit, ccrZ-LgBit plsC-SmBit, ccrZ-LgBit scrA-SmBit and *ccrZ-LgBit spv_1621-SmBit*. All fourteen double LgBit-SmBit labeled strains were constructed as followed. *SmBit* fragment was amplified by PCR from *ftsZ-SmBit* genome with primers 71/72 when using BsmbI and 73/74 when using BsaI (for *dnaA-SmBit and ezrA-SmBit*). *cps2E, hlpA*, *dnaA, ezrA, ftsA, ftsH, ftsW, pepN, pbp2x, zapA, fruA, plsC, scrA* and *spv_1621* upstream (of Start codon) regions were amplified from D39V genome with primers 75/76, 79/80, 83/84, 87/88, 91/92, 95/96, 99/100, 103/104, 107/108, 111/112, 115/116, 119/120, 123/124 and 127/128, respectively, and downstream regions were amplified from D39V genome with primers 77/78, 81/82, 85/86, 89/90, 93/94, 97/98, 101/102, 105/106, 109/110, 113/114, 117/118, 121/122, 125/126 and 129/130, respectively. Each downstream and upstream fragment was assembled to *SmBit* fragment using Golden Gate assembly (BsmBI for all except *dnaA* and *ezrA* using BsaI) and transformed into strain *ccrZ-LgBit*.

hlpA-LgBit and *hlpA-LgBit hlpA-SmBit*. For construction of the final strain *hlpA-LgBit hlpA-SmBit* used as positive control for SplitLuc assay as HlpA proteins interact together[11], a first strain *hlpA-LgBit* was made as followed. *Prs1* upstream region was amplified by PCR with primers 131/132 on strain VL2429 (Veening lab collection) and fragment *LgBit* fuse to chloramphenicol resistant marker, together with *prs1* downstream region was amplified with primers 135/136 from strain VL2429. *hlpA* was amplified with primers 133/134 from D39V genome. All three fragments were assembled by overlapping PCR using primers 131/136 and transformed into D39V wild type. *hlpA-SmBit* was amplified from strain *ccrZ-LgBit hlpA-SmBit* with primers 79/82 and transformed into the previously created strain *hlpA-LgBit*, leading to strain *hlpA-LgBit hlpA-SmBit*.

P_{lac}-*dnaA-SmBit*. For insertion of *dnaA* fused to *SmBit* under control of P_{lac} promoter, plasmid pPEPZ[2] was amplified by PCR with primers 222/223 (upstream) and 224/225 (downstream) and *dnaA-SmBit* was amplified by PCR with primers 226/227 from strain *ccrZ-LgBit dnaA-SmBit*. All three fragments were assembled using Golden Gate (BsmBI) and inserted into D39V *lacI* strain, allowing for insertion of P_{lac}-*dnaA-SmBit* into the *zip* locus (pPEPZ integration platform - causing disruption of the non-coding gene *spv_2417*).

P_{lac}-*dnaA-SmBit yabA-LgBit* and *yabA-LgBit*. For fusion of *yabA* with *LgBit*, *LgBit* fragment was amplified by PCR with primers 228/229 from strain *ccrZ-LgBit* and *yabA* together with upstream and downstream regions were amplified with primers 230/231 and 232/233 respectively. All three fragments were assembled using Golden Gate (BsmBI) and inserted into strain P_{lac}-*dnaA-SmBit* and D39V, leading to strains P_{lac}-*dnaA-SmBit yabA-LgBit* and *yabA-LgBit*.

yabA-LgBit ccrZ-SmBit. *ccrZ-SmBit* fragment, containing upstream and downstream regions, was amplified from *ccrZ-SmBit* strain using primers 62/67 and transformed into strain *yabA-LgBit*, resulting in strain *yabA-LgBit ccrZ-SmBit*.

hlpA-mKate2 Δ*ccrZ*. *ccrZ* ::*ery* fragment was amplified by PCR with primers 10/11 on strain *ccrZ*$^{-/+}$ and transformed into *hlpA-mKate2* strain[1].

P_{Zn}-*ccrZ*$^{+/+}$ *ftsZ-cfp*, P_{Zn}-*ccrZ*$^{+/+}$ *ftsZ-cfp hlpA-mKate2* and P_{Zn}-*ccrZ*$^{-/+}$ *ftsZ-cfp hlpA-mKate2*. *ftsZ-cfp* was amplified from *ftsZ-cfp* strain[1] by PCR with primers 12/15 and transformed into strain P_{Zn}-*ccrZ*$^{+/+}$ leading to strain P_{Zn}-*ccrZ*$^{+/+}$ *ftsZ-cfp*. *hlpA-mKate2* was amplified by PCR from strain *hlpA-mKate2*[1] with primers 137/138 and the resulting fragment was used for transformation of strain P_{Zn}-*ccrZ*$^{+/+}$ *ftsZ-cfp*. The newly created strain P_{Zn}-*ccrZ*$^{+/+}$ *ftsZ-cfp hlpA-mKate2* was then transformed in presence of IPTG with *ccrZ*::*ery* fragment amplified by PCR with primers 10/11 on strain *ccrZ*$^{-/+}$, resulting in strain P_{Zn}-*ccrZ*$^{-/+}$ *ftsZ-cfp hlpA-mKate2*.

ccrZ^{supp1}, *ccrZ*^{supp2} and *ccrZ*^{supp3}. The deletion fragment *ccrZ*::*ery* was amplified by PCR with primers 10/11 on strain *ccrZ*$^{-/+}$ and inserted in place of *ccrZ* in strain D39V wild type. Among the 10,000 colonies appearing on erythromycin-agar plates, 200 colonies were as large as wild type colony, while nearly 9,800 were very small colonies. 3 large colonies were cultivated, and their growth was assessed by plate-reader assay, before to be stored at -80°C. Mutation determination is described in *Genome resequencing of ccrZ suppressors by NGS* section. *ccrZ*^{supp1} has a 741G>T substitution leading to DnaA-Q247H and an additional insertion 13_14insA into *licD2*; *ccrZ*^{supp2} has a 874G>T substitution leading to DnaA-S292G; and *ccrZ*^{supp3} had a 277G>T substitution leading to YabA E93* (insertion of STOP codon).

Δ*ccrZ dnaA-Q247H* and Δ*ccrZ dnaA-S292G*. For re-insertion of the two *dnaA* point mutations in wild type background, fragments corresponding to *dnaA-Q247H* and *dnaA-S292G* with flanking region (marker-less) were first constructed by PCR assembly. The fragment corresponding to *dnaA-Q247H*_up was amplified on D39V genome with primers 143/140 and *dnaA-Q247H*_down was amplified with primers 139/144; a final PCR with primers 143/144 using both fragments as template led to the final *dnaA-Q247H* product. *dnaA-S292G* fragment was constructed in the same way using primers 143/142 and 141/144. Either assemblies were then inserted into D39V wild type, together with the deletion fragment *ccrZ*::*ery* (amplified

by PCR with primers 10/11 on strain *ccrZ*$^{-/+}$), allowing for selection. In both cases, small colonies with few large colonies (2%) were present and only the large colonies were selected for confirmation by PCR and sequencing.

kan-ccrZ, dnaA-Q247H and *dnaA-S292G*. In order to re-insert *ccrZ* into strain Δ*ccrZ dnaA-Q247H* and Δ*ccrZ dnaA-S292G*, a strain *kan-ccrZ* was constructed as intermediate. The upstream region of *ccrZ*, together with *kan* (kanamycin resistant marker), was amplified by PCR on strain *gfp-ccrZ* with primers 45/145 and *ccrZ* gene was amplified with primers 146/11 on D39V genome. Both fragments were then assembled using Golden Gate assembly (BsmBI) and inserted into D39V wild type. *kan-ccrZ* fragment was amplified by PCR from the resulting strain *kan-ccrZ* with primers 22/55. Strains Δ*dnaA Q247H* and Δ*dnaA S292G* were then transformed with the obtained DNA fragments and selected for kanamycin resistance, allowing for replacement of *ccrZ*::*ery* with *kan-ccrZ*.

Δ*yabA* and Δ*yabA* Δ*ccrZ*. For *yabA* deletion, sequences upstream and downstream of *yabA* were amplified on D39V genome by PCR with primers 147/148 and 151/152, respectively, and spectinomycin resistance marker was amplified on pPEP23 with primers 149/1509. All fragments were then assembled using Golden Gate assembly (BsmBI) and inserted into D39V wild type. Δ*yabA* strain was then transformed with a fragment *ccrZ*::*ery* amplified by PCR with primers 10/11 on strain *ccrZ*$^{-/+}$ resulting in strain Δ*yabA* Δ*ccrZ*.

ccrZ-N164A, *ccrZ-H157A* and *ccrZ-D177A*. Insertion of *ccrZ* point mutations into P_{Zn}-*ccrZ*$^{+/+}$ strain was performed as followed. For *ccrZ-N164A*, *kan-ccrZ_up* was amplified on *kan-ccrZ* genome with primers 45/154 and *ccrZ_down* was amplified with primers 153/11 on D39V genome. Both fragments were assembled by PCR assembly using primers 45/11. *ccrZ-H157A* and *ccrZ-D177A* were constructed in a similar manner, with primers 45/156, 155/11, 45/158 and 157/11 respectively. All three assembled products were then inserted into strain P_{Zn}-*ccrZ*$^{+/+}$ in presence of $ZnCl_2$ in order to keep high CcrZ levels.

P_{lac}-*ccrZ-gfp*, P_{lac}-*ccrZ-H157A-gfp*, P_{lac}-*ccrZ-N164A-gfp* and P_{lac}-*ccrZ-D177A-gfp*. For insertion of *ccrZ* fused to *gfp* under control of P_{lac} promoter, *ccrZ-gfp* was amplified from P_{Zn}-*ccrZ-gfp* strain genome with primers 161/162 and plasmid pPEPZ[2] was amplified by PCR with primers 159/160. Assembly of both fragments using Golden Gate (BsmBI) and insertion into D39V *lacI* strain allowed insertion of P_{lac}-*ccrZ-GFP* into the *zip* locus (pPEPZ integration platform - causing disruption of the non-coding gene *spv_2417*). To mutate *ccrZ-gfp*, PCR assembly was used for the three mutants. For P_{lac}-*ccrZ-H157A-gfp*, upstream of the *zip* locus together with P_{lac}-*ccrZ_up* were amplified by PCR with primers 163/156 and *ccrZ-gfp_down* with downstream region of *zip* locus was amplified with primers 155/164, from P_{lac}-*ccrZ-gfp* genome. Both fragments were assembled by PCR assembly using primers 163/164. P_{lac}-*ccrZ-N164A-gfp* and P_{lac}-*ccrZ-D177A-gfp* fragments were constructed in a similar manner, using primers 163/154, 153/164, 163/158 and 157/164 respectively. All three products were then inserted into strain D39V *lacI*.

comCDE-parS$_p$ *ccrZ-mKate2*, *parB*$_p$*-YFP comCDE-parS*$_p$ *ccrZ-mKate2* and *dnaN-mTQ*^{ox} *parB*$_p$*-YFP comCDE-parS*$_p$ *ccrZ-mKate2*. In order to visualize the replication machinery together with the origin of replication and CcrZ, a first strain was made, expressing CcrZ-mKate2 and containing $parS_p$ sites from *Lactococcus lactis* (able to bind $parB_p$ proteins from *L. lactis*) in the genome close to the origin of replication *oriC*. *comCDE-parS*$_p$ was amplified by PCR from strain D39V, *comCDE-parS*$_p$ *bgaA*::*parB*$_p$*-sfmGFP*[1]

using primers 165/166 and the resulting fragment was transformed into strain *ccrZ-mKate2*. Plasmid pMK19-02 (carrying *bgaA*::*P_{Zn}-parB$_p$-msfYFP*, Veening Lab collection) was transformed into the resulting strain *comCDE-parS$_p$ ccrZ-mKate2*. To prevent any alteration in replication process, the genetic fusion of *dnaN* with *sfmTurquoise2^{ox}* was introduced as a second copy downstream of the original *dnaN* gene. *dnaN* and upstream region were amplified by PCR with primers 167/168 from D39V genome, the second *dnaN* copy was amplified with primers 169/170 from D39V genome, *sfmTurquoise2^{ox}* with linker were amplified with primers 173/174 from strain D39V, *CEP*::*P3-spv_1159-sfmTurquoise2^{ox}-opt* (Veening lab collection), chloramphenicol resistance marker (*cam*) was amplified with primers 175/176 from *hlpA-mKate2* strain and downstream of *dnaN* was amplified with primers 171/172 from D39V genome. All five fragments were assembled using Golden Gate assembly (BsaI for the three first fragments and BsaI / SapI for the two others) and transformed into strain *parB$_p$-YFP comCDE-parS$_p$ ccrZ-mKate2*.

ccrZ-mKate2 P_{Zn}-dnaA-GFP. *dnaA* was amplified from D39V genome by PCR with primers 177/178 and the resulting fragment was digested with NotI-SpeI restriction enzymes and ligated into plasmid pMK17, allowing genetic fusion with *msfgfp* under control of $ZnCl_2$-inducible promoter. The resulting product was transformed into strain *ccrZ-mKate2*.

$ccrZ_{Sa}$^{sgRNA} and *$ccrZ_{Sa}$*^{sgRNA} *P_{ccrZSa}-$ccrZ_{Bs}$*. Inverse PCR was used to construct sgRNA-plasmid, as described previously[3,12]; in which the phosphorylated primer 179 was combined with gene specific forward primers containing the 20 bp targeting region as overhangs. Primer 180 was used as specific primer to construct the plasmid pCG248-sgRNA(*$ccrZ_{Sa}$*). The sgRNA was designed to target the 5' end *$ccrZ_{Sa}$*. For construction of the complementation plasmid pCG248-sgRNA(*$ccrZ_{Sa}$*)-*P_{ccrZ}-$ccrZ_{Bs}$*, *B. subtilis ccrZ*-homolog, *ytmP* (*$ccrZ_{Bs}$*), was fused to the *$ccrZ_{Sa}$* promoter and integrated into plasmid pCG248-sgRNA(*$ccrZ_{Sa}$*). *$ccrZ_{Bs}$* was amplified from plasmid pSG3174 (pUC19-*$ccrZ_{Bs}$*, see below) using primers 183/184. *$ccrZ_{Sa}$* -promoter (*P_{ccrZSa}*) was amplified from *S. aureus* SH1000 genome using primers 181/182. Both fragments were fused in a second PCR step, using primers 181/184. The resulting fragment was digested with restriction enzymes BamHI and KpnI and ligated into plasmid pCG248-sgRNA(*$ccrZ_{Sa}$*).

P_{lac}-gfp-$ccrZ_{Sa}$. For expression of CcrZ-GFP under control of an IPTG-inducible promoter in *S. aureus* SH1000, *$ccrZ_{Sa}$* gene was amplified from genomic DNA of *S. aureus* SH1000 using primers 185/186. The fragment was digested with NcoI and BamHI and ligated into the corresponding sites of plasmid pLOW-*parB-msfgfp*. The resulting plasmid expressing the translational fusion $CcrZ_{Sa}$-msfGFP fusion under control of an IPTG-inducible promoter was then transformed into SH1000 strain.

P_{tet}-dCas9, P_{lac}-ccrZ, ΔccrZ. A *S. pneumoniae* strain expressing lacI and tetR under control of the constitutive promoter PF6 (*prs1*::PF6-*lacI-tetR*) was transformed with a DNA fragment *P_{tet}-dCas9,* amplified by PCR from strain VL2212[13] (D39V, *prs1*::*PF6-tetR*, *bgaA::P_{tet}-dcas9*) with primers 219/220, that integrated in the *bgaA* region. The resulting *P_{tet}-dCas9* strain was then transformed with a PCR fragment *P_{lac}-ccrZ* amplified with primers 217/218 from strain *ccrZ*$^{+/+}$. This fragment integrated in place of the *CIL* locus. The resulting strain *P_{tet}-dCas9, P_{lac}-ccrZ* was then transformed in presence of 0.1 mM IPTG with a PCR fragment *ccrZ::ery* amplified with primers 10/11 from strain *ccrZ*$^{-/+}$, resulting in strain *P_{tet}-dCas9, P_{lac}-ccrZ, ΔccrZ.*

All *B. subtilis* strains were constructed by integrating into the chromosome by double homologous recombination using either genomic DNA or linear DNA fragments possessing two ~1 kb region homology. Assembled products were introduced into parent strains using natural transformation. Constructs were confirmed by PCR and the resulting fragment sequenced.

1A700 Δ*ccrZ*$_{Bs}$. In frame deletion of *ccrZ* homolog in *B. subtilis*, *ytmP* (*ccrZ*$_{Bs}$), was performed using Golden Gate allelic replacement strategy as described before[14]. Upstream homology region of *ccrZ*$_{Bs}$ was amplified by PCR with primers 187/188 and ligated into plasmid pUC19 (leading to pSG3174), 5' region and 3' region (including downstream homology of *ccrZ*$_{Bs}$) were amplified with primers pairs 189/190 and 191/192 and ligated into plasmid pUC19 (leading to pSG3178 and pSG3177, respectively). The three resulting plasmids and plasmid pSG0682[14] (carrying an erythromycin resistance cassette) where assembled together with Golden Gate backbone plasmid pSG1525[14] and transformed into 1A700 wild type.

ccrZ$_{Bs}$*-gfp*. For translational fusion of *ccrZ*$_{Bs}$ with msfGFP, *ccrZ*$_{Bs}$ (*ytmP*) was amplified by PCR with primers 193/194 and ligated into plasmid pUC19 (leading to pSG3175), *msfgfp* was amplified with primers 195/196 and ligated into plasmid pUC19 (giving pSG3179) and homology region downstream of *ccrZ*$_{Bs}$ was amplified with primers pair 189/190 and ligated into pUC19 (leading to pSG3176). The three plasmids obtained and plasmid pSG0682 were assembled using Golden Gate and transformed into 1A700 wild type strain.

Δ*ccrZ*$_{Bs}$. Deletion of *ccrZ*$_{Bs}$ was constructed by replacing the open reading frame with a chloramphenicol resistance cassette (cam) by using linear Gibson assembly fragments containing ~1KB of flanking homology for *ccrZ*$_{Bs}$. Upstream of *ytmP* (*ccrZ*$_{Bs}$) was amplified using primers 197/198; the chloramphenicol resistance cassette was amplified from pGEM*::cat*[15] using primers 199/200; downstream of *ccrZ*$_{Bs}$ was amplified using primers 201/202.

oriN and *oriN* Δ*ccrZ*$_{Bs}$. Strain *oriN* was constructed by introducing the heterologous origin and initiator (*oriN/repN*)[16] in place of *oriC* and introducing a constitutive promoter to drive expression of *dnaN*. The constitutive promoter used was Ppen[17] with the following sequence replacing the -10 to -35 box (5'-GTTGCATTTATTCTTAGATAGTGTAATACT-3'). The various fragments were amplified by PCR and assembled using Gibson assembly. The fragments were amplified using the following primers and templates: upstream *dnaA* was amplified using primer 203/204, kanamycin cassette was amplified from pGK67[18] using primers 205/206, *oriN*/*repN* was amplified from pDL110[16] using primers 207/208, Ppen2027 was amplified from CAL2072 using 209/210 and *dnaN* was amplified with primers 211/212. The assembled fragments were then transformed into strain JH642 (*oriN*) or Δ*ccrZ*$_{Bs}$ (*oriN* Δ*ccrZ*$_{Bs}$).

Supplementary Table 1: Strains and plasmids used in this study

S. pneumoniae strains	Relevant genotype	Reference
D39V wild type	*S. pneumoniae* serotype 2	[19]
R6 wild type	*S. pneumoniae* D39 derivative	gift from C. Grangeasse laboratory, Lyon France
TIGR4 wild type	*S. pneumoniae* serotype 4	[20]
ccrZ^{sgRNA}	D39V, *prs1*::*lacI*, *bgaA::P$_{lac}$-dCas, CEP::P3-ccrZ-sgRNA*; Gen^R, Tet^R, Spc^R	3
P$_{Zn}$-ccrZ$^{+/+}$	D39V, *bgaA*::*P$_{Zn}$-spv_0476*; Tet^R	This study
P$_{Zn}$-ccrZ$^{-/+}$	D39V, *bgaA*::*P$_{Zn}$-spv_0476*, Δ*spd_0476*; Tet^R, Ery^R	This study
D39V *lacI*	D39V, *prs1*::*lacI*; Gen^R	3
ccrZ$^{+/+}$	D39V, *prs1*::*lacI*, *cil*::*P$_{lac}$-spv_0476*; Gen^R, Kan^R	This study
ccrZ$^{-/+}$	D39V, *prs1*::*lacI*, *cil*::*P$_{lac}$-spv_0476*, Δ*spd_0476*; Gen^R, Kan^R, Ery^R	This study
ftsZ-spc	D39V, *ftsZ-spc*; Spc^R	This study
ftsZ-mTurquoise2	D39V, *ftsZ*::*ftsZ-mTurquoise2*; Spc^R	This study
ccrZ$^{-/+}$ *ftsZ-mTurquoise2*	D39V, *prs1*::*lacI*, *cil*::*P$_{lac}$-spv_0476*, Δ*spd_0476*, *ftsZ*::*ftsZ-mTurquoise2*; Gen^R, Kan^R, Ery^R, Spc^R	This study
dnaA^{TS}	D39V, *dnaA*::*dnaA-M398T*	7
dnaA^{TS} *ftsZ-mTurquoise2*	D39V, *dnaA* thermosensitive; *ftsZ*::*ftsZ-mTurquoise2; Spc*^{R}	This study
ccrZ$^{-/+}$ Δ*trmB*	D39V, *prs1*::*lacI*, *cil*::*P$_{lac}$-spv_0476*, *spv_0476-trmB*::*ery*; Gen^R, Kan^R, Ery^R	This study
P$_{Zn}$-ccrZ-gfp	D39V, *bgaA*::*P$_{Zn}$-spv_0476-msfgfp*; Tet^R	This study
P$_{Zn}$-gfp-ccrZ	D39V, *bgaA*::*P$_{Zn}$-msfgfp-spv_0476*; Tet^R	This study
P$_{Zn}$-ftsZ-gfp	D39V, *bgaA*::*P$_{Zn}$-ftsZ-msfgfp*; Tet^R	This study
R6 *P$_{Zn}$-gfp-ccrZ*	R6, *bgaA::P$_{Zn}$-msfGFP-spv_0476;* Tet^R	This study
TIGR4 *P$_{Zn}$-gfp-ccrZ*	TIGR4, *bgaA::P$_{Zn}$-msfGFP-spv_0476;* Tet^R	This study
gfp-ccrZ	D39V, *spv_0476*::*msfgfp-spv_0476*; Kan^R	This study
ftsZ-mCherry	D39V, *ftsZ*::*ftsZ-mCherry*; Kan^R	1
gfp-ccrZ ftsZ-mCherry	D39V, *spv_0476*::*msfgfp-spv_0476*, *ftsZ*::*ftsZ-mCherry*; Kan^R, Ery^R	This study
ftsZ$^{+/+}$	D39V, *prs1*::*lacI*, *cil*::*P$_{lac}$-ftsZ*; Gen^R, Kan^R	This study
ftsZ$^{-/+}$	D39V, *prs1*::*lacI*, *cil*::*P$_{lac}$-ftsZ*, Δ*ftsZ*; Gen^R, Kan^R, Spc^R	This study
ftsZ-mKate2	D39V, *ftsZ*::*ftsZ-mKate2*; Ery^R	1
ccrZ-mKate2	D39V, *spv_0476*::*spv_0476-mKate2*; Ery^R	This study
ftsZ$^{-/+}$ *ccrZ-mKate2*	D39V, *prs1*::*lacI*, *cil*::*P$_{lac}$-ftsZ*, Δ*ftsZ*, *spv_0476*::*spv_0476-mKate2*; Gen^R, Spc^R, Kan^R, Ery^R	This study
ccrZ-LgBit	D39V, *spv_0476*::*spv_0476-LgBit*; Ery^R	This study
ftsZ-SmBit	D39V, *ftsZ*::*ftsZ-SmBit*; Spc^R	This study
ccrZ-LgBit ftsZ-SmBit	D39V, *spv_0476*::*spv_0476-LgBit*, *ftsZ*::*ftsZ-SmBit*; Ery^R, Spc^R	This study
ccrZ-LgBit ccrZ-SmBit	D39V, *spv_0476*::*spv_0476-LgBit-spv_0476-SmBit*; Ery^R, Spc^R	This study
ccrZ-LgBit cps2E-SmBit	D39V, *spv_0476*::*spv_0476-LgBit*, *cps2E*::*cps2E-SmBit*; Ery^R, Spc^R	This study
ccrZ-LgBit hlpA-SmBit	D39V, *spv_0476*::*spv_0476-LgBit*, *hlpA*::*hlpA-SmBit*; Ery^R, Spc^R	This study
ccrZ-LgBit dnaA-SmBit	D39V, *spv_0476*::*spv_0476-LgBit*, *dnaA*::*dnaA-SmBit*; Ery^R, Spc^R	This study

ccrZ-LgBit ezrA-SmBit	D39V, *spv_0476*::*spv_0476-LgBit*, *ezrA*::*ezrA-SmBit*; Ery^R, Spc^R	This study
ccrZ-LgBit ftsA-SmBit	D39V, *spv_0476*::*spv_0476-LgBit*, *ftsA*::*ftsA-SmBit*; Ery^R, Spc^R	This study
ccrZ-LgBit ftsH-SmBit	D39V, *spv_0476*::*spv_0476-LgBit*, *ftsH*::*ftsH-SmBit*; Ery^R, Spc^R	This study
ccrZ-LgBit ftsW-SmBit	D39V, *spv_0476*::*spv_0476-LgBit*, *ftsW*::*ftsW-SmBit*; Ery^R, Spc^R	This study
ccrZ-LgBit pepN-SmBit	D39V, *spv_0476*::*spv_0476-LgBit*, *pepN*::*pepN-SmBit*; Ery^R, Spc^R	This study
ccrZ-LgBit pbp2x-SmBit	D39V, *spv_0476*::*spv_0476-LgBit*, *pbp2x*::*pbp2x-SmBit*; Ery^R, Spc^R	This study
ccrZ-LgBit zapA-SmBit	D39V, *spv_0476*::*spv_0476-LgBit*, *zapA*::*zapA-SmBit*; Ery^R, Spc^R	This study
ccrZ-LgBit fruA-SmBit	D39V, *spv_0476*::*spv_0476-LgBit*, *fruA*::*fruA-SmBit*; Ery^R, Spc^R	This study
ccrZ-LgBit plsC-SmBit	D39V, *spv_0476*::*spv_0476-LgBit*, *plsC*::*plsC-SmBit*; Ery^R, Spc^R	This study
ccrZ-LgBit scrA-SmBit	D39V, *spv_0476*::*spv_0476-LgBit*, *scrA*::*scrA-SmBit*; Ery^R, Spc^R	This study
ccrZ-LgBit spv_1621-SmBit	D39V, *spv_0476*::*spv_0476-LgBit*, *spv*_1621::*spv*_1621-*SmBit*; Ery^R, Spc^R	This study
comCDE-LgBit	D39V, *prs1*::P_{comC}-*comC-comD-comE-LgBit*; Cam^R	Veening Lab collection
hlpA-LgBit	D39V, *prs1*::P_{hlpA}-*hlpA-LgBit*; Cam^R	This study
hlpA-LgBit hlpA-SmBit	D39V, *prs1*::P_{hlpA}-*hlpA-LgBit*, *hlpA*::*hlpA-SmBit;* Cam^R, Spc^R	This study
D39V *lacI ccrZ-LgBit*	D39V, *prs1*::*lacI, spv_0476*::*spv_0476-LgBit;* Ery^R, Gen^R	This study
ccrZ-LgBit P_{lac}-*ftsZ*	D39V, *prs1*::*lacI, spv_0476*::*spv_0476-LgBit, cil*::P_{lac}-*ftsZ*; Gen^R, Ery^R, Kan^R	This study
P_{lac}-*dnaA-SmBit*	D39V, *prs1*::*lacI*, *zip*::P_{lac}-*dnaA-SmBit*; Gen^R, Spc^R	This study
P_{lac}-*dnaA-SmBit yabA-LgBit*	D39V, *prs1*::*lacI*, *zip*::P_{lac}-*dnaA-SmBit, yabA*::*yabA-LgBit*; Gen^R, Spc^R, Ery^R	This study
yabA-LgBit	D39V, *yabA*::*yabA-LgBit*; Ery^R	This study
yabA-LgBit ccrZ-SmBit	D39V, *yabA*::*yabA-LgBit, spv_0476*::*spv_0476-SmBit*; Spc^R, Ery^R	This study
P3-gfp	D39V, *cep*::*P3-sfgfp*; Spc^R	5
hlpA-mKate2	D39V, *hlpA*::*hlpA-hlpA-mKate2*; Cam^R	[21]
hlpA-mKate2 Δ*ccrZ*	D39V, *hlpA*::*hlpA-hlpA-mKate2*; Δ*spd_0476*; Cam^R, Ery^R	1
ftsZ-cfp	D39V, *ftsZ*::*ftsZ-cfp*; Kan^R	1
P_{Zn}-*ccrZ*$^{+/+}$ *ftsZ-cfp*	D39V, *bgaA*::P_{Zn}-*spv_0476*, *ftsZ*::*ftsZ-cfp*; Tet^R, Kan^R	This study
P_{Zn}-*ccrZ*$^{+/+}$ *ftsZ-cfp hlpA-mKate2*	D39V, *bgaA*::P_{Zn}-*spv_0476*, *ftsZ*::*ftsZ-cfp, hlpA*::*hlpA-hlpA-mKate2*; Tet^R, Kan^R, Cam^R	This study
P_{Zn}-*ccrZ*$^{-/+}$ *ftsZ-cfp hlpA-mKate2*	D39V, *bgaA*::P_{Zn}-*spv_0476*, *ftsZ*::*ftsZ-cfp, hlpA*::*hlpA-hlpA-mKate2*, Δ*spd_0476*; Tet^R, Kan^R, Cam^R, Ery^R	This study
ccrZ^{supp1}	D39V, Δ*spd_0476*, *dnaA*::*dnaA-Q247H,* 13_14insA *licD2*; Ery^R	This study
ccrZ^{supp2}	D39V, Δ*spd_0476*, *dnaA*::*dnaA-S292G*; Ery^R	This study
ccrZ^{supp3}	D39V, Δ*spd_0476*, *yabA*::*yabA-E93**; Ery^R	This study
Δ*ccrZ dnaA-Q247H*	D39V, Δ*spd_0476*, *dnaA*::*dnaA-Q247H*; Ery^R	This study
Δ*ccrZ dnaA-S292G*	D39V, Δ*spd_0476*, *dnaA*::*dnaA-S292G*; Ery^R	This study
kan-ccrZ	D39V, *spv_0476*::*kan-spv_0476*; Kan^R	This study
dnaA-Q247H	D39V, *spv_0476*::*kan-spv_0476*, *dnaA*::*dnaA-Q247H*; Kan^R	This study
dnaA-S292G	D39V, *spv_0476*::*kan-spv_0476*, *dnaA*::*dnaA-S292G*; Kan^R	This study
Δ*yabA*	D39V, Δ*yabA*; Spc^R	This study
Δ*yabA* Δ*ccrZ*	D39V, Δ*yabA*, Δ*spd_0476*; Spc^R, Ery^R	This study
ccrZ-N164A	D39V, *bgaA*::P_{Zn}-*spv_0476*, *spv_0476*::*kan-spv_0476-N164A*; Tet^R, Kan^R	This study

ccrZ-H157A	D39V, *bgaA*::P_{Zn}-*spv_0476*, *spv_0476*::*kan-spv_0476-H157A*; Tet^R, Kan^R	This study
ccrZ-D177A	D39V, *bgaA*::P_{Zn}-*spv_0476*, *spv_0476*::*kan-spv_0476-D177A*; Tet^R, Kan^R	This study
P_{lac}-*ccrZ-gfp*	D39V, *prs1*::*lacI*, *zip*::P_{lac}-*spv_0476-msfgfp*; Gen^R, Spc^R	This study
P_{lac}-*ccrZ-H157A-gfp*	D39V, *prs1*::*lacI*, *zip*:: P_{lac}-*spv_0476-H157A-msfgfp*; Gen^R, Spc^R	This study
P_{lac}-*ccrZ-N164A-gfp*	D39V, *prs1*::*lacI*, *zip*:: P_{lac}-*spv_0476-N164A-msfgfp*; Gen^R, Spc^R	This study
P_{lac}-*ccrZ-D177A-gfp*	D39V, *prs1*::*lacI*, *zip*:: P_{lac}-*spv_0476-D177A-msfgfp*; Gen^R, Spc^R	This study
comCDE-parS$_p$ *ccrZ-mKate2*	D39V, *spv_0476::spv_0476-mKate2, comCDE::comCDE-parS*$_p$; Ery^R	This study
parB$_p$*-YFP comCDE-parS*$_p$ *ccrZ-mKate2*	D39V, *spv_0476::spv_0476-mKate2, comCDE::comCDE-parS*$_p$, *bgaA::*P_{Zn}-*parB*^{p}*-msfYFP*; Ery^R, Tet^R	This study
dnaN-mTQ^{ox} *parB*$_p$*-YFP comCDE-parS*$_p$ *ccrZ-mKate2*	D39V, *spv_0476::spv_0476-mKate2, comCDE::comCDE-parS*$_p$, *bgaA::*P_{Zn}-*parB*^{p}*-msfYFP,* ; Ery^R, Tet^R, Cam^R	This study
comCDE-parS$_p$ *parB*$_p$*-GFP*	D39V, *comCDE-parS*$_p$ *bgaA::parB*$_p$*-sfmGFP*; Kan^R	1
spv_1159-mTurquoise2^{ox}	D39V, *CEP*::*P3-spv_1159-sfmTurquoise2*^{ox}*-opt;* Spc^R	Veening Lab collection
ccrZ-mKate2 P_{Zn}*-dnaA-GFP*	D39V, *ccrZ::ccrZ-mKate2, bgaA::*P_{Zn}*-dnaA-msfGFP;* Ery^R, Tet^R	This study
P_{tet}*-dCas9*	*D39V, prs1::PF6-lacI-tetR, bgaA::Ptet-dcas9; Gen*^{R}*, Tet*^{R}	This study
P_{tet}*-dCas9,* P_{lac}*-ccrZ*	*D39V, prs1::PF6-lacI-tetR, bgaA::Ptet-dcas9, cil::Plac-spv_0476; Gen*^{R}*, Tet*^{R}*, Kan*^{R}	This study
P_{tet}*-dCas9,* P_{lac}*-ccrZ,* Δ*ccrZ*	*D39V, prs1::PF6-lacI-tetR, bgaA::Ptet-dcas9, cil::Plac-spv_0476, Δspd_0476; Gen*^{R}*, Tet*^{R}*, Kan*^{R}*, Ery*^{R}	This study
***S. aureus* strains**		
SH1000	*S. aureus* SH1000	[22]
SH1000 *dcas9*	SH1000, *pLOW-dcas9;* Ery^R	12
ccrZ$_{Sa}^{sgRNA}$	SH1000, *pLOW-dcas9*, *pCG248-sgRNA(ccrZ*$_{Sa}$*);* Ery^R, Cam^R	This study
ccrZ$_{Sa}^{sgRNA}$ P_{ccrZSa}*-ccrZ*$_{Bs}$	SH1000, *pLOW-dcas9*, *pCG248-sgRNA(ccrZ*$_{Sa}$*)-*P_{ccrZSa}*-ccrZ*$_{Bs}$*;* Ery^R, Cam^R	This study
P_{lac}*-gfp-ccrZ*$_{Sa}$	SH1000, *pLOW-m(sf)gfp-ccrZ*$_{Sa}$*;* Ery^R	This study
***B. subtilis* strains**		
1A700	*B. subtilis* 1A700	Gruber lab collection
1A700 Δ*ccrZ*$_{Bs}$	1A700, Δ*ytmP*; Ery^R	This study
ccrZ$_{Bs}$*-gfp*	1A700, *ytmP-msfgfp*; Ery^R	This study
JH642	*B. subtilis* JH642	[23]
JH642 Δ*ccrZ*$_{Bs}$	JH642, Δ*ytmP*; Cam^R	This study
oriN	JH642, *oriC::oriN-repN, Ppen-dnaN*; Kan^R	This study
oriN, Δ*ccrZ*$_{Bs}$	JH642, *oriC::oriN-repN, Ppen-dnaN, ΔytmP*; Cam^R; Can^R	This study
dnaB134	*dnaB* temperature sensitive mutant	Grossman lab collection
**Plasmids**		
pPEPY	Vector with *Plac,* allowing integration into *S. pneumoniae* CIL locus (*spv_2166-spv_2165*); Kan^R	[24]
pPEPZ	Vector with *Plac,* allowing integration into *S. pneumoniae* ZIP locus (spv_2416-*spv_2419*); Spc^R	[24]
pCG6	pMK17 derivative, encoding *ccrZ* fused to 3'of *msfyfp;* Tet^R	3

pMK11	Vector with P_{Zn} (P_{czcD}) allowing integration into *S. pneumoniae bgaA* locus; Tet^R	1
pMK17	pMK11 derivative, encoding *msfgfp;* Tet^R	1
pMK19-02	pMK17 derivative, encoding *parB* fused to *msfyfp;* Tet^R	Veening Lab collection
pLOW-dcas9	*dcas9* downstream of P_{lac} promoter; Amp^R, Ery^R	12
pCG248-sgRNA(ccrZ$_{Sa}$)	For constitutive expression of sgRNA(*ccrZ$_{Sa}$*); Amp^R, Cam^R	This study
pCG248-sgRNA(ccrZ$_{Sa}$)-P$_{ccrZSa}$-ccrZ$_{Bs}$	For constitutive expression of sgRNA(*ccrZ$_{Sa}$*), *ccrZ$_{Bs}$* (*ytmP*) expressed from P_{ccrZSa}; Amp^R, Cam^R	This study
pLOW-*msggfp-parB*	*msfgfp-parB* fusion downstream of P_{lac} promoter; Amp^R, Ery^R	Kjos Lab collection
pGK67	pGEM derivative, carrying a kanamycin resistance cassette; Cam^R	18
pDL110	pJH101 derivative, carrying *oriN-repN* from pBPA23; Amp^R	[25]
pUT18	Encoding CyaA T18 domain under control of P_{lac}; Amp^R	[26]
pST25	Encoding CyaA T25 domain under control of P_{lac}; Spc^R	[26]
pUC19	pUC18 derivative, inverted MCS, Amp^R	Addgene
pJET1.2	pJET1.2	Invitrogen
pSG1525	pET-Gate2 derivative with MazEF toxin-antitoxin system for Golden gate assembly selection; Kan^R	14
pSG0682	pJET1.2 derivative with erythromycin (*ermB*) cassette; Ery^R	14
pSG3174	pUC19 derivative, carrying *ccrZ$_{Bs}$* (*ytmP*); Amp^R	This study
pSG3177	pUC19 derivative, carrying sequence of upstream region of *ccrZ$_{Bs}$* (*ytmP*); Amp^R	This study
pSG3178	pUC19 derivative, carrying sequence of downstream region of *ccrZ$_{Bs}$* (*ytmP*); Amp^R	This study
pSG3175	pUC19 derivative, carrying *ccrZ$_{Bs}$* (*ytmP*) with no STOP codon for fusion with *msfgfp*; Amp^R	This study
pSG3176	pUC19 derivative, carrying sequence of downstream region of *ccrZ$_{Bs}$* (*ytmP*); Amp^R	This study
pSG3179	pUC19 derivative, carrying *msfgfp*; Amp^R	This study
pSG2949	pET-Gate2 derivative, encoding *ccrZ* with C-terminal cysteine protease domain (CPD) with a 10his tag ; Kan^R	This study
pSG2950	pET-Gate2 derivative, encoding *ccrZ* fused to *sfgfp* with a 10his tag ; Kan^R	This study
pSG4227	pJET1.2 derivative, carrying *ftsZ* from *S. pneumoniae*; Amp^R	This study
pSG1694	pET-Gold1 derivative encoding a *ccdB* cassette, used for Golden gate assembly ; Amp^R	Gruber Lab collection
pSG4268	pETGold1 derivative encoding *ftsZ* from *S. pneumoniae ;* Amp^R	This study
pSG436	pET-Gate2 derivative encoding *ccdB* cassette ; Kan^R	Gruber Lab collection
pSG366	pJET1.2 derivative carrying short sequence2 for Golden gate assembly ; Amp^R	Gruber Lab collection
pSG367	pJET1.2 derivative carrying short sequence3 for Golden gate assembly ; Amp^R	Gruber Lab collection
pSG2559	pET-Gold1 derivative carrying C-terminal cysteine protease domain (CPD) with a 10his tag; Amp^R	Gruber Lab collection
pSG2562	pET-Gold1 derivative carrying *sfGFP* with a 10his ; Amp^R	Gruber Lab collection
***E. coli* strains**		

HM1784	BTH101 derivative, F^-, *cya-99*, *araD139*, *galE15*, *galK16*, *rpsL1*, *hsdR2*, *mcrA1*, *mcrB1,* Δ*rnh*::*kan*; Str^R	26
IM08B	K12 DH10B derivative, *mcrA,* Δ(*mrr-hsdRMS-mcrBC*), φ80*lacZ*Δ*M15,* Δ*lacX74, recA1, araD139,* Δ(*ara-leu*)7697, *galU, galK, rpsL, endA1, nupG,* Δ*dcm*, Ω*Phelp-hsdMS* (CC8-2), Ω*PN25-hsdS* (CC8-1); Str^R	27
BL21 DE3 Gold	B $F^–$, *ompT, hsdS*($r_B^–$ $m_B^–$), *dcm*$^+$, *gal*λ(DE3), *endA,* Hte; Tet^R	Agilent Technologies

Abbreviations: Tet^R: tetracycline resistance; Kan^R: kanamycin resistance; Cam^R: chloramphenicol resistance; Ery^R: erythromycin resistance; Spc^R: spectinomycin resistance; Gen^R: gentamycin resistance; Amp^R: ampicillin resistance; SmBit: Small Bit; LgBit: Large Bit

Supplementary Table 2: Oligonucleotides used in this study

Primer name	forward / reverse	Sequence (5' -> 3')
1	F	CGATGGAATTCGAAACTTTATACGGAGGAAAGAAATG
2	R	GCGCACTAGTTCATCTTCTCTTTCCATACTTGTCT
3	F	CACTTATTTTCCTAGATTCCA
4	R	TGTTCATATGAAAATTCCTCCGGGCGCGACTTCTTTCCTCCGTATAAAGTTTCCT
5	F	AGTTATCTATTATTTAACGGGAGGAAATAAGACAAGTATGGAAAGAGAAGATGAG
6	R	CATATATTCCATAGTCAACTC
7	F	GTCGCGCCCGGAGGAATTTTCATATG
8	R	TTATTTCCTCCCGTTAAATAATAGA
9	R	GCGCGGATCCTCATCTTCTCTTTCCATACTTGTCT
10	F	CATTTCTTCGCTGTTTCTTC
11	R	GGAAATATTGTTCTGGGAAG
12	F	CGGATTTCCAACAAGCTTCA
13	R	GGAACGTCGTCTCCGTTTTAACGATTTTTGAAAAATGGAGG
14	F	GGAACGTCGTCTCGTAGTTTTCAAAAATCGTTAAGTAAATGAATG
15	R	CCAAATAGTCCAAACAACGAC
16	F	GGAACGTCGTCTCGAAACGAGGTGAAATCATGAGC
17	R	GGAACGTCGTCTCGACTAATTGAGAGAAGTTTCTATAGAATTTT
18	R	GGATTCCCGTCTCCACTCCTTTAGCTGCAGCTTC
19	F	GGATTCCCGTCTCCGAGTGAGCAAGGGCGAGGAG
20	R	GGATTCCCGTCTCCGTTTTACTTGTACAGCTCGTCC
21	F	GGATTCCCGTCTCGAAACGAGGTGAAATCATGAGC
22	F	CAGCCATAGAGGAGATCATCATGTA
23	R	TGCGCTAGGTCTCCTTTCTTTCCTCCGTATAAAGTTTC
24	F	TGCGCTAGGTCTCGGAAATGAACAAAAATATAAAATATTCTCAAAACT
25	R	TGCGCTAGGTCTCGTCTTATTTCCTCCCGTTAAATAATAGAT
26	F	TGCGCTAGGTCTCGAAGAGATAGCCTAAAATTAGGCTG
27	R	GGCTGCTACACGACCAAACTCAG
28	F	GCATGCGGCCGCAAACTTTATACGGAGGAAAGAAATG
29	R	CGCCACTAGTTCTTCTCTTTCCATACTTGTCTCGG
30	F	GCATGCGGCCGCATAAAGAGGAAAAATAAATTATGAC
31	R	GCGCACTAGTACGATTTTTGAAAAATGGAGGTGTA
32	F	GCGCACTAGTGATTTGGGTGATAATGAGC
33	R	GCATGCGGCCGCTCATCATCTTCTCTTTCCATACTTGT
34	F	GTACTGCCTGCGTGGTTATC
35	R	ACGGATCCCCAGCTTGCGCGTCCTCTTCTCAAAGAAAAGCCTCTGGATTG
36	R	CTATTCGTCAACCACTCTAACCCGAAGATATAAAACATCAGAGTATGGAC
37	F	TCTGATGTTTTATATCTTCGGGTTAGAGTGGTTGACGAATAGGCCAAAAACTAGTAGAATAGTAAG GAAACTTTATACGGAGGAAAGAAATGAAACATCTTAGCTCAAAAGGAGAAGAGC
38	R	TCATCTTCTCTTTCCATACTTGTCTC
39	F	GAGGACGCGCAAGCTGGGGATCCGT
40	F	CGATGGAATTCAATAAAGAGGAAAAATAAATTATG
41	R	GCGCGGATCCTTAACGATTTTTGAAAAATGGAGGTG
42	F	TGTCCTCCGTCTCCTAACGAGGTGAAATCATGAGC
43	R	GGAAATTACGGATCAAGATG
44	R	TGTCCTCCGTCTCCGTTAATTTATTTTTCCTCTTTATTCGTCA
45	F	GCTATGTGGTCGGATTTTGGTT
46	R	CAGGACACGTCTCGATCTTCTCTTTCCATACTTGTCT
47	F	CAGGACACGTCTCGAGATCCGGATCTGGTGGAGAA
48	R	CAGGACACGTCTCGACTTATTTCCTCCCGTTAAATAATAGAT
49	F	CAGGACACGTCTCCAAGTATGGAAAGAGAAGATGAGAGT
50	R	CCAAACGGCTATCAAATACTTCA
51	F	GCTATGTGGTCGGATTTTGGTT
52	F	CTTTGAGAATCGGGTTAGAG

53	R	GCTGTCAGGTCTCGCCTCTTCTCTTTCCATACTTGTCT
54	F	GCTGTCAGGTCTCCTAACCCGGAGGAATTTTCA
55	R	GGCCATGGATCTGAAAAGTTC
56	F	GGAAGGTCGTCTCCTCGTACAACAGATTCAGTCGTTT
57	R	TGCCGATCGTCTCCTACATCGCTG
58	F	GATCGGAAGCATGTTTGACG
59	R	GGAAGGTCGTCTCCACGAACAATCGATTCACGGCG
60	F	TGCCGATCGTCTCGTGTAGATGTTCTAGTCGTCGTGAATC
61	R	CCAGCTAAAACATGACAAGC
62	F	GACAATTCTTGGTTAGGGCT
63	R	TGAGGGTCGTCTCCCTCTTCTCTTTCCATACTTGTCT
64	F	TGAGGGTCGTCTCCAGAGGCGGCTCATCAGGCGGCG
65	R	TGAGGGTCGTCTCCACCTAATTGAGAGAAGTTTCTATAG
66	F	TGAGGGTCGTCTCCAGGTATGGAAAGAGAAGATGAGAGT
67	R	ATTCGGACGGTTTTCTTACGCG
68	F	GATACCCAAGCCAATCCAGAGGC
69	R	TGAGGGTCGTCTCGGTTATTTCCTCCCGTTAAATAATAGAT
70	F	TGAGGGTCGTCTCCTAACTTTATACGGAGGAAAGAAATGG
71	F	CCCGTATCGTCTCTGGCGGCTCATCAGGCGGCGGC
72	R	CCCGTATCGTCTCCCTAATTGAGAGAAGTTTCTATAG
73	F	CCCGTATGGTCTCTGGCGGCTCATCAGGCGGCGGC
74	R	CCCGTATGGTCTCCCTAATTGAGAGAAGTTTCTATAG
75	F	CTACTTAGTGCTGTGAGAGAAGC
76	R	CCCGTATCGTCTCCCGCCCTTCGCTCCATCTCTCA
77	F	CCCGTATCGTCTCGTTAGTTTACTTTTGTTTTAGACTACTAG
78	R	GCTTCTGCAATTCGTTGGCTAG
79	F	AGCATAAATAGCAGCACCTA
80	R	CCCGTATCGTCTCCCGCCTTTAACAGCGTCTTTAAGAGCTTTACC
81	F	CCCGTATCGTCTCGTTAGTCAGTCTTTAAAAAGCCTATTGTATC
82	R	GCGATGGTTTCAATATCCAAGTG
83	F	GGCTGTATCAGCCGCTTTAGCTGTC
84	R	CCCGTATGGTCTCCCGCCTTTGATTTCTTTTTGATTGATTCA
85	F	CCCGTATGGTCTCGTTAGTTTGTGGATAACTTTTAGTTTTTATC
86	R	CACCAGTAACCTGATCAGTATCGA
87	F	GGCCTTAAGTCATATTGTGGATCGT
88	R	CCCGTATGGTCTCCCGCCAAAACGAATCGTTTCACGTGTTTTC
89	F	CCCGTATGGTCTCGTTAGTAAAAGAAAAAGATTTATTGTGTGAG
90	R	TGCCTCTGCAGCCTTCAAATGGCGG
91	F	GATGCAGCAGCAACTGCTATC
92	R	CCCGTATCGTCTCCCGCCTTCGTCAAACATGCTTCCGATC
93	F	CCCGTATCGTCTCGTTAGAGAGGAAAAATAAATTATGAC
94	R	CTCACGGTAGTTAGTAGCAGATCTA
95	F	GAGGTAATGACGAACGTGAACAAAC
96	R	CCCGTATCGTCTCCCGCCTTTTTCGTCATTCATTTTTGACTTTAC
97	F	CCCGTATCGTCTCGTTAGCCCTGAGAGAGGCTGGAGCCTC
98	R	TTAGGCACGCCGCCAGCGTTCGTCC
99	F	GCGCCTTGCAGTTGGTTCGAAACC
100	R	CCCGTATCGTCTCCCGCCCTTCAACAGAAGGTTCATTGGTTG
101	F	CCCGTATCGTCTCGTTAGGATAAAGAAAGGATAGTTTATGTCTC
102	R	GAATATCAAACAAGGCACGACGG
103	F	CACTGAAAATACTGCCCACTATATT
104	R	CCCGTATCGTCTCCCGCCTGCATTTCCGTATTGAAGAACAACTGC
105	F	CCCGTATCGTCTCGTTAGATAAGCCTAAAATAAAAAGAAAACTCAGC
106	R	CGATAGATTTCTTCCTGCCCATAGC
107	F	GAAGGATTGACTGGTGGCAGAATG
108	R	CCCGTATCGTCTCCCGCCGTCTCCTAAAGTTAATGTAATTTTT
109	F	CCCGTATCGTCTCGTTAGTATGTTTATTTCCATCAGTGCTG

110	R	CATCAGCTTCGCCTTATCTACAACC
111	F	GAGATCACGCGCAATGACAGAAGAA
112	R	CCCGTATCGTCTCCCGCCTAAGGAATCCTCAATCTTGCTCTGT
113	F	CCCGTATCGTCTCGTTAGCAGAGCAAGATTGAGGATTCCTTATG
114	R	CGACGGATTCGCGCCAATTCTTCAC
115	F	GATGAGTGGTGTATCTCAAATG
116	R	CCCGTATCGTCTCCCGCCTGCTTGTGGTTTGCGTAGGTAACCA
117	F	CCCGTATCGTCTCGTTAGAAAAATAGAAAAATGAAAAGATTGG
118	R	CAAAGCTAGCAAAGGTTGCTTCTAA
119	F	GGTCATGAGATTGAGCTTGTCC
120	R	CCCGTATCGTCTCCCGCCTGCAAGTTCTTCTCTTTTCTTATCT
121	F	CCCGTATCGTCTCGTTAGAAGAAATGAACCTTGGCCAAACAGC
122	R	TTCCTACGTTCGACATTACCCACTA
123	F	CATGTCTATCCTTGGACTCTTTGTC
124	R	CCCGTATCGTCTCCCGCCGATTTTCACTTCGATCACAGCATCCC
125	F	CCCGTATCGTCTCGTTAGTCAATCCTCTCTAATGTGAAAACG
126	R	GTCGCTACCGTCCGTAATCACTTAA
127	F	GACCTGCCATGAGTTTGTTGAAACT
128	R	CCCGTATCGTCTCCCGCCAAGAGTATAGGCCATGGCCCCTGC
129	F	CCCGTATCGTCTCGTTAGAAATCTCTTTAAACCATGTCAGC
130	R	GCGGCTGTCGCAGAGTTGAGACAAA
131	F	ATGTCTTTTTCTGATTTAAAGCTGTTTGCCC
132	R	CTATTTCTTCCAATTTTATTTATTTTGAAGGCGAATGCTCTATCCAGC
133	F	GCTGGATAGAGCATTCGCCTTCAAAATAAATAAAATTGGAAGAAATAG
134	R	GCCGCCGCCGCCTGATGAGCCGCCTTTAACAGCGTCTTTAAGAGCTTTAC
135	F	GTAAAGCTCTTAAAGACGCTGTTAAAGGCGGCTCATCAGGCGGCGGCGGC
136	R	CTTGTCCCCACTGACAATGGTAATATC
137	F	GATTGTAACCGATTCATCTG
138	R	GGAATGCTTGGTCAAATCTA
139	F	CAAGCAAAAACATATTGTCCTAACG
140	R	CGTTAGGACAATATGTTTTTGCTTG
141	F	GCCATTTTACAACGTAAAACGGAAC
142	R	GTTCCGTTTTACGTTGTAAAATGGC
143	F	TCTTCTTTTATCCCCAACCTG
144	R	GAGTCTGTGAAATCTGTGGA
145	R	ACCCTGACGTCTCGTTTCTTTCCTCCGTATAAAGTTTC
146	F	ACCCTGACGTCTCCGAAATGGATTTGGGTGATAATGAGC
147	F	GCTACTTCTCTATATTGCCAGTC
148	R	CACGAACCGTCTCGCATAAAACAGCCCTTTCCTTTCTTTATCATTC
149	F	CACGAACCGTCTCGTATGAGCAATTTGATTAACGGAAAAATAC
150	R	CACGAACCGTCTCGGCCTAATTGAGAGAAGTTTCTATAGAATTTTTC
151	F	CACGAACCGTCTCCAGGCATGCAGATTCAAAAAAGTTTTAAGG
152	R	CCCTTTCTTTCATCTTCTTCCC
153	F	GTACGACATAGTGCTTGGATTGAGAC
154	R	GTCTCAATCCAAGCACTATGTCGTAC
155	F	GCGACCATTGTCGCTGGAGATGTACG
156	R	CGTACATCTCCAGCGACAATGGTCGC
157	F	GATTTATTTAGTAGCTTGGGATTCGG
158	R	CCGAATCCCAAGCTACTAAATAAATC
159	R	GCGTCACGTCTCAGCATTATTTTTCCTCCTTATTTAT
160	F	GCGTCACGTCTCACGGATCCCTCCAGTAACTCGAGAA
161	F	GCGTCACGTCTCAATGCAAACTTTATACGGAGGAAAGAAATGG
162	R	GCGTCACGTCTCATCCGGCTTACTTATAAAGCTCATCCATGCC
163	F	GCCAATAAATTGCTTCCTTGTTTTG
164	R	AATAATTCTTTCTTTGACACCGTATATG
165	F	GATGTTATTGAACATCAAGTGAGACTAGAG
166	R	AATCGCCATCTTCCAATCCC

167	F	AATTGCGGCCGCAGGAGGTTATCATGTCAATTTATCAAGAATTTG
168	R	AGTGGGTCTCCCTTTCACTTAATTTGTACGAACTGG
169	F	AGTGGGTCTCGAAAGGAGAATCCATGATTCATTTTT
170	R	AGTGGGTCTCGATCCATTTGTACGAACTGGTGTAATG
171	F	GGTTGCTCTTCGGAAAGAGGTTGAGCCTGGCTC
172	R	CAAAGGCGTCTTCACGTCCTTG
173	F	TCACGGTCTCGGGATCCGGATCTGGTGGA
174	R	TCACGGTCTCGTAGCTTATTATTATTTGTATAGTTCGTCCATGC
175	F	TCACGGTCTCCGCTAGTAGGAGGCATATCAAATGA
176	R	TGAGGCTCTTCGTTCTGCCGCTTATAAAAGCCAG
177	F	GCATGCGGCCGCAATAGTAAAGGAGGAGAAAGGATTG
178	R	CGCCACTAGTTTTGATTTCTTTTTGATTGATTCA
179	R	TATAGTTATTATACCAGGGGGACAGTGC
180	F	GCTGATAATGCCGCAATAAAGTTTAAGAGCTATGCTGGAAACAG
181	F	TCGAGGATCCTAGAGTTTATCGCCTACAGAG
182	R	GTAATTGTCCCAACCAGTTCAT CTCGTCCACCTCACTTTCAA
183	F	TTGAAAGTGAGGTGGACGAG ATGAACTGGTTGGGACAATTAC
184	R	ACTGGGTACCTTAATCAACAATTCGTTTCATGAGGA
185	F	GATCCCATGGAGCAGTTTTATCAATTAGG
186	R	TCCGGGGATCCAAATAAACATGTTACTATTCATAACT
187	F	GTTACAGAATTCGGTCTCGCGAGTAGCACCGATTGTTCCGGAACC
188	R	GTTACATCTAGAGGTCTCGTATTTCAAGCAAAACTCTTAAGATCTGATGC
189	F	GTTACAGAATTCGGTCTCGAGGTCATATCCTCATGAAACGAATTGTTG
190	R	GTTACATCTAGAGGTCTCTATGGCAATATCTGCGTTTTCAGCC
191	R	GTTACAGAATTCGGTCTCGCCTGTAATCAGGAATACGAATCCATTTGC
192	R	GTTACATCTAGAGGTCTCGACCTAGTAATTGTCCCAACCAGTTCAT
193	F	GTTACAGAATTCGGTCTCGTCTACGAAGTTTGTGCTATAGCGCTAAAATTTAAAG
194	R	GTTACATCTAGAGGTCTCGCTCCATCAACAATTCGTTTCATGAGG
195	F	GTTACAGAATTCGGTCTCAGGAGCAGGCGGTAGCGGCAGCGGTGGCTCA
196	R	GTTACATCTAGAGGTCTCTCAGGTCATTTGTATAGTTCATCCATGC
197	F	CAAATCGAGACTGACGTATGTCAG
198	R	TCAGGAATACGAATCCATTTGCTC
199	F	GGGTAGAGCAAATGGATTCGTATTCCTGAGGCTTAACTATGCGGCATCAG
200	R	GCTGGATAGACTAGAAACGATTAGCCCGGTATTTTCTCCTTACGCATCTG
201	F	GGCTAATCGTTTCTAGTCTATCCAG
202	R	CAAGCTGTGATGCATGGAATTG
203	F	TACGCCAACCATACTTAATAGCA
204	R	CGCAACTGTCCATACTCTGATGTCTATTATGGTTGCAAGAAATAAAAG
205	F	CTTTTATTTCTTGCAACCATAATAGACATCAGAGTATGGACAGTTGCG
206	R	GGAAAAGATTTTAGGAGGAAGCTGAACCATTTGAGGTGATAGGTAAG
207	F	GTTGAACTAATGGGTGCAGCTTCCTCCTAAAATCTTTTCCCAT
208	R	CGTTTTTTCGGAAAGAAGAATATGTAGAAGAAGTTATTGATG
209	F	CTTCTACATATTCTTCTTTCCGAAAAAACGGTTGCATTTA
210	R	TCCTCCTAACGGATAATGTATGCAGCCGACTCAAACATCAAATC
211	F	GCTGCATACATTATCCGTTAGGAGGATAAAAATGAAATTCACGAT TCAAAAAGATCGTC
212	R	CTGTATCAATTGAAATCGGATTTGC
213	F	CTCGAGGGTCTCACGAGAATAATTTTGTTTAACTTTAAGAAGGAGATATACATATGGATTTGGGTGA TAATGAGCTAACACTG
214	R	GCTGCAGGTCTCAGCCAGCTCTTCTCTTTCCATACTTGTCTCGGAAATG
215	F	GCAGCCCGTCTCATATGATGACATTTTCATTTGATACAGCTGCTGC
216	R	CGTCTCACAGGTTAACGATTTTTGAAAAATGGAGGTGTATCC
217	F	GCATCAACATCTTGTGTGGCACG
218	R	GCTTGGTTAATCAAACACGCCC
219	F	GCACTCAAACCTAGAAGAGC
220	R	CAACTCACATGAACTACATGATG
222	F	GCCAATAAATTGCTTCCTTGTTTTG
223	R	GCGTCACGTCTCAGCATTATTTTTCCTCCTTATTTAT

224	F	GCGTCACGTCTCACGGATCCCTCCAGTAACTCGAGAA
225	R	AATAATTCTTTCTTTGACACCGTATATG
226	F	GCGTCACGTCTCAATGCAATAGTAAAGGAGGAGAAAGGATTG
227	R	GCGTCACGTCTCATCCGTTATTATAGGATTTCTTCGA
228	F	CCCTGTACGTCTCCagggctcatcaggcggcggcg
229	R	CCCTGTACGTCTCCgcTTATTTCCTCCCGTTAAATAATAGATAAC
230	F	gatgtcaccttgattaagccag
231	R	CCCTGTACGTCTCCccctccctgtatagcaactcg
232	F	CCCTGTACGTCTCGAAgcatgcagattcaaaaaagtt
233	R	gtcaacttcatcagctctcttatct
OT1	F	ACGGTCTATCCCAGCTGTTG
OT2	R	ATAGGCGCGTGCTTCTTCTA
OT3	F	GAAAAGTACCATCCCCAGCA
OT4	R	AGCCTTGGTGCCTATCATTG
OT5	F	TGCATATCCGCGTCAAATAG
OT6	R	GCATGAATGACGGTCGTATG
OT7	F	CGCGTGTAGGTATTGCGTTA
OT8	R	CTTCGCGCACTTGAATAACA
OT9	F	TTGCCGCAGATTGAAGAG
OT10	R	AGGTGGACACTGCAAATAC
OT11	F	CGCGCTGACTCTGATATTATG
OT12	R	CAAAGAGGAGCTGCTGTAAC

Supplementary Table 3: Ratio of spectral counts between GFP-CcrZ and GFP from LC-MS/MS

Identified Proteins	Fold change (GFP-CcrZ / GFP)
SPV_0476 (CcrZ)	27
ScrA	12
PepN	8.5
Pbp2X	8.3
FruA	7.4
EzrA	7.4
SPV_1621	6.8
FtsZ	6.6
PlsC	6
FtsH	5.1
DnaA	2.8

Supplementary Table 4. CRISPRi-seq results for *ccrZ*-complementation vs *ccrZ*-depletion

Significance of differential sgRNA depletion/enrichment (absolute $\log_2 FC > 1$, FDR-adjusted P value < 0.05) was tested with the DESeq2 package (see Methods).

sgRNA Locus Tag	sgRNA Genes Name	ccrZ + FC	ccrZ - FC	ΔFC	adjusted p-value
SPV_0826	holB	-2.17930	0.68752	2.88021	1.8E-23
SPV_0827	yabA	-0.59237	1.90685	2.50661	1.1E-21
SPV_0878	orfX	-0.55568	-2.93204	-2.35409	1.2E-07
SPV_1694, SPV_1683, SPV_1686, SPV_1691, SPV_1689, SPV_1682, SPV_1684, SPV_1692, SPV_1688, SPV_1695, SPV_1685, SPV_1690, SPV_1693, SPV_1687	tRNA-Thr-2, tRNA-Ile-1, tRNA-fMet-1, tRNA-Arg-3, tRNA-Met-1, tRNA-Ser-2, tRNA-Gly-1, tRNA-Leu-2, tRNA-Ile2-1, tRNA-Leu-3, tRNA-Phe-1, tRNA-Pro-1, tRNA-Gly-2, tRNA-Ser-3	-2.89305	-0.90916	1.99195	6.4E-06
SPV_1428	cmk	-1.97401	-0.27583	1.71463	2.0E-05
SPV_1885	tRNA-fMet-2	-3.43986	-1.49497	1.95470	5.5E-05
SPV_0506	pheT	-2.48212	-0.78336	1.70863	8.0E-05
SPV_1416, SPV_1417	murT, gatD	-1.92621	-0.19268	1.75009	8.0E-05
SPV_1477	sepF	-3.55870	-1.58888	1.98277	2.4E-04
SPV_0721	folD	-1.65240	-0.18280	1.48331	4.7E-04
SPV_0825	tmk	-3.86986	-1.89413	1.98323	7.1E-04
SPV_2447	srf-27	-2.31680	-0.47976	1.84793	8.2E-04
SPV_0098	unknown	-2.12298	-0.49091	1.64505	2.5E-03
SPV_0774	ftsK	-0.17089	-1.70687	-1.51904	2.5E-03
SPV_1039, SPV_1040	ptsI, ptsH	-3.17079	-1.28447	1.90596	3.4E-03
SPV_1216, SPV_1217	alaS, unknown	-2.97262	-1.31148	1.67317	3.4E-03
SPV_1548, SPV_1547	gmk, rpoZ	-2.89919	-1.12040	1.78808	3.4E-03
SPV_1123, SPV_1127, SPV_1125, SPV_1124, SPV_1126	licC, tarI, licA, licB, tarJ	-1.68717	-0.14800	1.55259	1.4E-02
SPV_1128	tacF	-1.64076	-0.16633	1.48908	1.8E-02
SPV_1305, SPV_2339, SPV_1303, SPV_1304	glyQ, unknown, unknown, glyS	-2.68473	-1.06266	1.63681	2.3E-02
SPV_0346, SPV_0349, SPV_0347, SPV_0348	mvk, fni, mvaD, mvaK2	-0.73104	0.66488	1.41140	3.5E-02
SPV_1129	licD1	-1.25224	0.17989	1.44633	3.5E-02
SPV_0967	murA-1	-0.74607	0.70383	1.46592	3.9E-02
SPV_1475	ylmH	-3.10233	-1.26574	1.85460	3.9E-02
SPV_1476	ylmG	-3.37976	-1.69753	1.69454	4.2E-02
SPV_0099	aatA	-2.23984	-0.61836	1.63300	5.2E-02
SPV_1484	ddl	-5.03792	-3.13035	1.92305	5.2E-02
SPV_0968	unknown	-0.40840	0.94624	1.36594	6.6E-02
SPV_1373	aspC	-2.66016	-1.17033	1.50242	6.8E-02
SPV_0248	glmS	-2.36386	-0.85340	1.52023	9.9E-02
SPV_0477, SPV_0476	trmB, unknown	-1.59225	-0.13290	1.48177	1.0E-01
SPV_1790	rpmH	-2.32602	-0.82953	1.51143	1.3E-01
SPV_1639	sczA	-2.11368	-0.53646	1.59522	1.3E-01
SPV_0526	fba	-1.90239	-0.53794	1.37655	1.5E-01
SPV_1791	unknown	-3.65783	-1.94843	1.71258	1.5E-01
SPV_1358	ytgP	-1.92015	-0.53300	1.40307	1.6E-01
SPV_1940, SPV_1941	unknown, aspS	-2.89975	-1.33455	1.57645	2.4E-01
SPV_2421	srf-23	-2.98851	-1.47692	1.52779	2.7E-01
SPV_2330	srf-20	-4.22250	-2.43257	1.80152	3.3E-01
SPV_1374	unknown	-2.78366	-1.38349	1.41504	4.0E-01
SPV_1886	tRNA-Ser-4	-3.43400	-1.96408	1.48260	4.2E-01
SPV_2449	srf-28	-1.21815	0.11773	1.35248	5.2E-01
SPV_0664	metK	-2.55487	-1.14934	1.41806	5.4E-01
SPV_0244, SPV_0243, SPV_0245, SPV_0246	cdsA, uppS, eep, proS	-2.78046	-1.38060	1.41361	6.1E-01
SPV_2066	tRNA-Arg-5	-2.87606	-1.58010	1.30915	6.1E-01
SPV_1200, SPV_1197, SPV_1198, SPV_1199, SPV_1201	unknown, tarQ, tarP, cps23FU, licD3	-0.80883	0.56746	1.39351	6.2E-01
SPV_0834	pyrH	-1.84418	-0.47869	1.37997	6.5E-01
SPV_1041, SPV_1042, SPV_1043	nrdH, nrdE, nrdF	-5.31669	-3.61374	1.71968	7.9E-01
SPV_1472	ileS	-3.23326	-1.83165	1.41615	9.0E-01
SPV_0019	rrfA	-2.82999	-1.44210	1.40219	9.5E-01
SPV_1628, SPV_1629	NA, pbuX	-0.04137	0.05785	0.11339	1
SPV_0001, SPV_0002	dnaA, dnaN	-2.87828	-3.49810	-0.60566	1
SPV_0003	unknown	0.13287	-0.21255	-0.32910	1
SPV_0004	ychF	-0.21524	-0.05853	0.17299	1

SPV_0005	pth	0.04314	0.25657	0.22820	1
SPV_0006	mfd	0.03865	0.16602	0.14424	1
SPV_0007, SPV_0008	unknown, divIC	-0.46091	0.15123	0.62513	1
SPV_0010	unknown	-0.42058	-0.08736	0.35188	1
SPV_0011	tilS	-0.22678	-0.08684	0.15099	1
SPV_0012	hpt	-0.61879	-0.68118	-0.05081	1
SPV_0013	ftsH	-0.25060	-0.37279	-0.11047	1
SPV_0014	comX1	0.12194	0.13112	0.02359	1
SPV_0016	rrsA	-0.26622	-0.55192	-0.27175	1
SPV_0017	tRNA-Ala-1	0.08545	-0.08251	-0.15582	1
SPV_0018	rrlA	-3.04883	-2.04468	1.01509	1
SPV_0025, SPV_0026	unknown, scRNA	-0.02723	0.12527	0.16124	1
SPV_0026	scRNA	-0.25914	-0.05052	0.22194	1
SPV_0029, SPV_0027, SPV_0028	radA, dut, unknown	-0.07174	0.06255	0.14911	1
SPV_0030, SPV_2079	unknown, prsW	-0.25432	0.76406	1.03112	1
SPV_0033	prs1	-2.00358	-1.06580	0.94864	1
SPV_0034	unknown	0.05659	0.10022	0.06248	1
SPV_0038	polA	-0.05992	-0.22017	-0.15087	1
SPV_0039	unknown	0.21695	-0.04187	-0.25231	1
SPV_0040	yeiH	0.01764	0.02924	0.02319	1
SPV_0041, SPV_0045, SPV_0043, SPV_0042, SPV_0044	araT, unknown, plsX, recO, unknown	-0.18768	-0.17497	0.02937	1
SPV_0046	blpU	0.21196	-0.03617	-0.22929	1
SPV_0047	unknown	0.05435	-0.03059	-0.06751	1
SPV_0060, SPV_0056, SPV_0057, SPV_0055, SPV_0054, SPV_0062, SPV_0052, SPV_0061, SPV_0051, SPV_0059, SPV_0053, SPV_0058	purK, vanZ, purH, purN, purM, purB, purL, unknown, purC, purE, purF, purD	-0.00973	0.10750	0.12774	1
SPV_0060, SPV_0062, SPV_0061, SPV_0059, SPV_0058	purK, purB, unknown, purE, purD	0.24252	0.07007	-0.15220	1
SPV_0062, SPV_0061	purB, unknown	-0.63402	-0.09203	0.55972	1
SPV_0063	strH	0.13033	0.06744	-0.04544	1
SPV_0064	cpsR	-0.20488	-0.16572	0.05407	1
SPV_0066, SPV_0065, SPV_0068, SPV_0067, SPV_0071, SPV_0069, SPV_0070	gadV, bgaC, gadE, gadW, galM, gadF, agaS	0.00431	-0.01246	-0.00262	1
SPV_0073	unknown	-0.00888	-0.00313	0.02176	1
SPV_0073, SPV_0072	unknown, unknown	0.07242	-0.07479	-0.13308	1
SPV_0074	unknown	0.03079	0.07028	0.05352	1
SPV_0076	unknown	0.01245	0.08752	0.08585	1
SPV_0077	cabP	0.02176	0.08028	0.07328	1
SPV_0080	pavB	0.02653	0.00699	-0.00076	1
SPV_0082, SPV_0081	unknown, unknown	0.06198	0.01506	-0.02824	1
SPV_0083	rpsD	-3.36441	-2.39486	0.98529	1
SPV_0086	unknown	0.04821	0.02632	-0.00673	1
SPV_0087	unknown	-0.00491	0.11419	0.13041	1
SPV_0088	unknown	0.21833	-0.05030	-0.24940	1
SPV_0089	unknown	0.21134	-0.01796	-0.21252	1
SPV_0090	unknown	-0.01205	0.04289	0.06088	1
SPV_0091	unknown	-0.09572	0.05070	0.15995	1
SPV_0092	unknown	-0.08541	0.06534	0.16691	1
SPV_0093, SPV_0095, SPV_0094, SPV_0096	ptvC, ptvA, ptvB, ptvR	0.08385	0.05394	-0.01715	1
SPV_0097	unknown	0.24712	0.00676	-0.22539	1
SPV_0101, SPV_0102, SPV_0103, SPV_2095	gph, sdhA, sdhB, unknown	0.08914	0.03150	-0.03805	1
SPV_0104	unknown	-0.00191	0.05235	0.06531	1
SPV_0106	unknown	-0.07350	0.01574	0.09804	1
SPV_0107, SPV_0108	unknown, unknown	-0.10741	0.10449	0.21994	1
SPV_0109	unknown	0.11159	0.19738	0.09766	1
SPV_0110	argG	0.03558	-0.02995	-0.04690	1
SPV_0111	argH	0.03371	0.11819	0.10138	1
SPV_0112	unknown	0.36199	-0.01985	-0.36709	1
SPV_0113	unknown	0.01663	-0.04900	-0.05171	1
SPV_0114	unknown	0.01649	0.06643	0.06621	1
SPV_0115	unknown	0.02838	0.06719	0.05230	1
SPV_0116	unknown	0.10109	0.03659	-0.05038	1
SPV_0117	unknown	0.17255	0.09601	-0.06442	1

SPV_0118	unknown	0.17230	0.04600	-0.11072	1
SPV_0123, SPV_2103, SPV_0124	unknown, unknown, unknown	-0.00752	0.03021	0.04781	1
SPV_0126	pspA	0.30846	0.05882	-0.23130	1
SPV_0129, SPV_0128, SPV_0127, SPV_2107	mnmG, unknown, mnmA, srf-04	-2.47899	-1.62238	0.86947	1
SPV_0129, SPV_0128, SPV_2107	mnmG, unknown, srf-04	0.05888	-0.07267	-0.11872	1
SPV_0130	rnjA	-1.88700	-0.70523	1.19623	1
SPV_0131	unknown	-1.46584	-0.44449	1.03742	1
SPV_0134	unknown	-0.89447	-0.78437	0.12516	1
SPV_0135	rimI	-0.67355	-0.72673	-0.04121	1
SPV_0136	tsaD	-0.80454	-0.76366	0.05595	1
SPV_0138	epsF	-0.13205	0.08622	0.23162	1
SPV_0139	unknown	0.00280	0.06856	0.07732	1
SPV_0140	unknown	0.12481	0.07802	-0.03251	1
SPV_0141	unknown	-0.15297	0.10075	0.26750	1
SPV_0142	unknown	-0.08599	0.00981	0.10649	1
SPV_0143	ugd	0.09886	0.10761	0.02337	1
SPV_0144	mutR1	0.36824	0.00816	-0.34571	1
SPV_0147, SPV_0148	unknown, unknown	-0.18271	0.08138	0.27579	1
SPV_0147, SPV_0148, SPV_0146	unknown, unknown, unknown	-0.04135	0.07338	0.12960	1
SPV_0147, SPV_0148, SPV_0146, SPV_0145	unknown, unknown, unknown, unknown	0.51005	-0.07544	-0.56539	1
SPV_0149	azlC	-1.00861	0.04576	1.07190	1
SPV_0150	gshT	0.23809	0.01105	-0.21319	1
SPV_0151	metQ	0.20129	0.01178	-0.17496	1
SPV_0152	dapE	0.20991	0.14256	-0.05421	1
SPV_0154, SPV_0153	metP, metN	0.26826	-0.01508	-0.26730	1
SPV_0155	unknown	0.41569	-0.01152	-0.41200	1
SPV_0157, SPV_0156, SPV_0158	unknown, unknown, unknown	-0.13360	0.08031	0.22644	1
SPV_0159	unknown	0.13495	0.16080	0.03752	1
SPV_0160	nrdI	0.14662	0.02383	-0.10111	1
SPV_0161	unknown	-0.08949	0.01644	0.12268	1
SPV_0163, SPV_0162	unknown, unknown	0.26470	0.09811	-0.14937	1
SPV_0165	hexB	-0.04935	0.02202	0.08222	1
SPV_0167, SPV_0168, SPV_0166, SPV_0169	ribB, ribE, ribH, ribD	0.11449	0.01852	-0.08197	1
SPV_0171, SPV_0170	tag, ruvA	-0.09515	-0.05244	0.05362	1
SPV_0172	unknown	-0.05388	-0.01198	0.05406	1
SPV_0173	unknown	0.13536	0.09854	-0.01957	1
SPV_0175, SPV_0174	corA1, unknown	-1.05038	-0.66909	0.38539	1
SPV_0177, SPV_0176	ypdF, uvrA	0.19855	0.06316	-0.12274	1
SPV_0178	spxA2	-0.82448	-0.48613	0.35199	1
SPV_0179	unknown	0.42592	-0.00557	-0.41248	1
SPV_0181, SPV_0182, SPV_0180	yqgF, unknown, unknown	0.18555	-0.53610	-0.70614	1
SPV_0183	folC	-0.18140	0.02948	0.22148	1
SPV_0184	unknown	-0.00190	0.06152	0.07873	1
SPV_0185	cls	0.17556	-0.23744	-0.39939	1
SPV_0186	unknown	0.14089	-0.08215	-0.20620	1
SPV_0189, SPV_0187, SPV_0190, SPV_0191, SPV_0188	unknown, nrdD, nrdG, unknown, unknown	-0.11068	-0.02552	0.09966	1
SPV_0191	unknown	0.24007	0.10513	-0.12004	1
SPV_0210, SPV_0204, SPV_0213, SPV_0206, SPV_0203, SPV_0209, SPV_0196, SPV_0192, SPV_0211, SPV_0194, SPV_0208, SPV_0202, SPV_0200, SPV_0212, SPV_0195, SPV_0199, SPV_0193, SPV_0201, SPV_0198, SPV_0205, SPV_0207, SPV_0197	rpsE, rplX, secY, rpsN, rplN, rplR, rplB, rpsJ, rpmD, rplD, rplF, rpsQ, rplP, rplO, rplW, rpsC, rplC, rpmC, rplV, rplE, rpsH, rpsS	-3.63622	-2.97779	0.67464	1
SPV_0210, SPV_0204, SPV_0213, SPV_0206, SPV_0203, SPV_0209, SPV_0196, SPV_0211, SPV_0194, SPV_0208, SPV_0202, SPV_0200, SPV_0212, SPV_0195, SPV_0199, SPV_0193, SPV_0201, SPV_0198, SPV_0205, SPV_0207, SPV_0197	rpsE, rplX, secY, rpsN, rplN, rplR, rplB, rpmD, rplD, rplF, rpsQ, rplP, rplO, rplW, rpsC, rplC, rpmC, rplV, rplE, rpsH, rpsS	-0.43576	-0.07678	0.36886	1

SPV_0210, SPV_0204, SPV_0213, SPV_0206, SPV_0203, SPV_0209, SPV_0211, SPV_0208, SPV_0202, SPV_0212, SPV_0201, SPV_0205, SPV_0207	rpsE, rplX, secY, rpsN, rplN, rplR, rpmD, rplF, rpsQ, rplO, rpmC, rplE, rpsH	-3.87978	-3.12225	0.77014	1
SPV_0214	adk	-2.91157	-1.76949	1.16353	1
SPV_0218, SPV_0217, SPV_0216, SPV_0219, SPV_0215, SPV_2122	rpoA, rpsK, rpsM, rplQ, infA, rpmJ	-3.20607	-2.55720	0.66782	1
SPV_0220, SPV_0221	unknown, unknown	-0.07474	0.09267	0.18442	1
SPV_0222	gpmB1	-0.00926	0.08474	0.10061	1
SPV_0225	unknown	0.20737	0.01988	-0.18005	1
SPV_0229, SPV_0228	unknown, unknown	0.12385	-0.05011	-0.15444	1
SPV_0230	deoR	0.06195	0.04663	0.00167	1
SPV_0231	unknown	-0.02640	0.05736	0.09501	1
SPV_0232	unknown	0.18563	0.01399	-0.15263	1
SPV_0233	unknown	-0.05175	0.18960	0.25043	1
SPV_0234	unknown	-0.19636	0.06000	0.26882	1
SPV_0235	unknown	0.03273	0.05266	0.03399	1
SPV_0236	talC	0.12664	0.00335	-0.10987	1
SPV_0237	gldA	0.03269	0.00016	-0.02383	1
SPV_0238	leuS	-2.81320	-1.88731	0.93930	1
SPV_0239	unknown	0.03761	0.07136	0.04797	1
SPV_0240	unknown	0.25363	0.03038	-0.20578	1
SPV_0241	ruvB	-0.04621	-0.32302	-0.25515	1
SPV_0242	unknown	-2.57690	-1.34596	1.24210	1
SPV_0247	bglA	0.28680	0.03702	-0.22748	1
SPV_0249	unknown	0.26918	0.09006	-0.16076	1
SPV_0250	spuA	0.28915	0.15102	-0.11746	1
SPV_0251, SPV_0252	rpsL, rpsG	-3.96070	-2.85478	1.11989	1
SPV_0253	fusA	-2.73332	-2.44312	0.30405	1
SPV_0254	polC	-1.89947	-1.11847	0.78593	1
SPV_0255	relB1	0.16283	0.02636	-0.12396	1
SPV_0256	relE1	-0.23048	-0.05823	0.18548	1
SPV_0257	unknown	-0.00551	0.13954	0.15884	1
SPV_0258	pepS	0.20382	0.13967	-0.05038	1
SPV_0259	unknown	0.09062	0.04130	-0.03832	1
SPV_0260	rsuA-1	0.22743	0.07510	-0.13665	1
SPV_0261	pepC	0.07948	-0.02226	-0.08659	1
SPV_0262, SPV_0263, SPV_0264	manN, manM, manL	-0.06821	0.11662	0.19813	1
SPV_0265	adhA	-0.03893	0.03450	0.08308	1
SPV_0266	unknown	-0.03319	0.04181	0.08934	1
SPV_0267	unknown	0.22413	0.02205	-0.18846	1
SPV_0268	unknown	0.09156	-0.08937	-0.16201	1
SPV_0269	sulA	-1.28587	-0.33511	0.95684	1
SPV_0270	sulB	-1.10273	-0.22310	0.89389	1
SPV_0271	sulC	-1.21005	-0.11633	1.10562	1
SPV_0272	sulD	-0.16295	0.07456	0.25938	1
SPV_0273	unknown	0.10487	0.04762	-0.04869	1
SPV_0274, SPV_0275	rplM, rpsI	-3.38618	-2.59039	0.81158	1
SPV_0276	unknown	0.13739	0.18552	0.06196	1
SPV_0283, SPV_0280, SPV_0278, SPV_0277, SPV_0281, SPV_0279, SPV_0282	celD, celR, unknown, celA, celC, celB, unknown	0.03771	0.18629	0.16386	1
SPV_0283, SPV_0280, SPV_0281, SPV_0279, SPV_0282	celD, celR, celC, celB, unknown	0.07666	0.03227	-0.02597	1
SPV_0283, SPV_0280, SPV_0281, SPV_0282	celD, celR, celC, unknown	0.03469	0.11611	0.09213	1
SPV_0284	unknown	0.14922	0.06032	-0.08200	1
SPV_0285	unknown	0.08721	0.11821	0.04326	1
SPV_0286	basA	0.05982	0.10308	0.06014	1
SPV_0287	spnHL	0.13870	0.03617	-0.08699	1
SPV_0289, SPV_0290, SPV_0291, SPV_0292	eda, unknown, unknown, unknown	-0.04923	0.05354	0.11503	1
SPV_0293	unknown	0.20802	-0.09008	-0.28112	1
SPV_0294	ugl	0.00278	0.14041	0.15015	1
SPV_0295	unknown	0.20158	0.00847	-0.17283	1
SPV_0296	unknown	0.14614	0.11207	-0.02300	1
SPV_0297	unknown	-0.10446	0.11126	0.22653	1

SPV_0298	yajC-1	0.12348	0.05316	-0.05029	1
SPV_0301	regR	0.02630	0.01488	0.00486	1
SPV_0302	unknown	0.18554	-0.04205	-0.21411	1
SPV_0303	unknown	0.22515	0.01381	-0.19721	1
SPV_0304	mraW	-6.58252	-4.96591	1.59861	1
SPV_0305	ftsL	-6.37725	-4.98683	1.40334	1
SPV_0307, SPV_0306	mraY, pbp2x	-5.55005	-4.60387	0.96889	1
SPV_0308	clpL	0.14329	0.02380	-0.10231	1
SPV_0309	luxS	0.00439	-0.06003	-0.04781	1
SPV_0310	unknown	-0.03550	0.06892	0.12006	1
SPV_0311, SPV_0313	dexB, unknown	-0.09099	0.06623	0.17048	1
SPV_0326, SPV_0329, SPV_0330, SPV_0323, SPV_0320, SPV_0317, SPV_0315, SPV_0319, SPV_0324, SPV_0328, SPV_0321, SPV_0322, SPV_0316, SPV_0327, SPV_0318, SPV_0325	cps2K, cps2M, cps2N, cps2H, cps2T, cps2C, cps2A, cps2E, cps2I, cps2L, cps2F, cps2G, cps2B, cps2P, cps2D, cps2J	0.34644	0.57611	0.23666	1
SPV_0326, SPV_0329, SPV_0330, SPV_0323, SPV_0324, SPV_0328, SPV_0321, SPV_0322, SPV_0327, SPV_0325	cps2K, cps2M, cps2N, cps2H, cps2I, cps2L, cps2F, cps2G, cps2P, cps2J	0.37310	0.36427	0.00362	1
SPV_0331	cps2O	-0.53262	-0.62518	-0.07676	1
SPV_0334	aliA	0.07127	0.07356	0.01649	1
SPV_0335	eng	0.12040	-0.05158	-0.15339	1
SPV_0337, SPV_0336	recU, pbp1a	-0.49687	-0.61120	-0.10342	1
SPV_0338	unknown	-0.00921	0.03462	0.05914	1
SPV_0339	gpsB	-1.73158	-0.50203	1.25508	1
SPV_0340	rnpB	-2.05910	-0.94412	1.12723	1
SPV_0341	rlmL	-0.00587	-0.20645	-0.18668	1
SPV_0342	mapZ	-0.08648	-0.25995	-0.15998	1
SPV_0344, SPV_0343	ritR, gnd	-4.07428	-2.53280	1.55838	1
SPV_0345	cbpF	0.27566	0.05064	-0.20944	1
SPV_0352, SPV_2148, SPV_0350, SPV_0351	vraR, alkD, vraT, vraS	0.09689	-0.12111	-0.20589	1
SPV_0355, SPV_0357, SPV_0356	unknown, cbpK, cbpG	0.01664	0.09716	0.09650	1
SPV_0358	unknown	0.29688	-0.08751	-0.37134	1
SPV_0360	mtlA	-0.02763	-0.00423	0.03783	1
SPV_0361	mtlR	-0.10601	-0.02359	0.09456	1
SPV_0362	mtlA2	0.30639	0.01025	-0.28032	1
SPV_0363	mtlD	0.20708	0.14682	-0.04485	1
SPV_0364	unknown	0.21433	0.10866	-0.09176	1
SPV_0365	tig	-0.07710	-0.54365	-0.45267	1
SPV_0366	yrrC	0.11179	-0.16587	-0.26078	1
SPV_0367, SPV_0368	lepB, rnhC	-1.43412	-0.78629	0.66173	1
SPV_0370, SPV_0369	zapB, zapA	0.06697	-0.09311	-0.14447	1
SPV_0371	mutS2	0.07682	0.00928	-0.05502	1
SPV_0372	glyP	-0.17912	-0.22218	-0.02580	1
SPV_0373	mip	0.21579	-0.04616	-0.24255	1
SPV_0374	shetA	0.28984	0.13805	-0.14036	1
SPV_0375	serS	-2.35755	-1.12159	1.25477	1
SPV_0376, SPV_0375, SPV_0377	manO, serS, lysC	-0.16951	0.18361	0.36561	1
SPV_0380, SPV_0378, SPV_0379, SPV_0381	fabH, fabM, fabT, acpP	0.13670	0.07010	-0.05159	1
SPV_0380, SPV_0379, SPV_0381	fabH, fabT, acpP	-1.13298	0.07042	1.21433	1
SPV_0385, SPV_0389, SPV_0388, SPV_0386, SPV_0383, SPV_0390, SPV_0384, SPV_0387, SPV_0382	fabF, accD, accC, accB, fabD, accA, fabG1, fabZ, fabK	-0.77397	-0.01569	0.77006	1
SPV_0393, SPV_0395, SPV_0394	nusB, efp, unknown	-0.59633	-0.64154	-0.03192	1
SPV_0397, SPV_0398, SPV_0396	gatA, gatC, gatB	-2.82509	-1.63406	1.20899	1
SPV_0399	prfC	0.14445	-0.10518	-0.23923	1
SPV_0400	unknown	-0.01860	0.02002	0.04937	1
SPV_0401	rpmB	-0.06776	-0.12400	-0.04493	1
SPV_0402	asp23	-0.16988	0.05183	0.23942	1
SPV_0403	unknown	-0.72419	0.02993	0.76777	1
SPV_0404, SPV_0406, SPV_2159, SPV_0405, SPV_0408	ilvB, ilvC, unknown, ilvH, unknown	0.25748	0.05257	-0.19343	1
SPV_0409	ilvA	-0.04539	0.07936	0.13685	1
SPV_0410	unknown	0.06594	-0.52094	-0.57124	1

SPV_0412, SPV_0411	glnHP1, glnQ1	0.28242	0.17193	-0.09530	1
SPV_0413	unknown	0.09110	0.13602	0.05953	1
SPV_0414	unknown	-0.05549	0.00900	0.07849	1
SPV_0417	uppP	-0.08128	0.43174	0.52786	1
SPV_0418	unknown	-0.12997	0.15774	0.29774	1
SPV_0419	dinP	0.05788	-0.04646	-0.08952	1
SPV_0420	pfl	0.45774	0.07944	-0.37325	1
SPV_0422	unknown	0.26254	-0.10224	-0.34890	1
SPV_0423	xylR	0.23092	0.15477	-0.05900	1
SPV_0424	unknown	0.10311	-0.05218	-0.14279	1
SPV_0425	unknown	0.11161	0.09851	0.01008	1
SPV_0426	lacF-1	-0.15460	0.15099	0.31843	1
SPV_0427	lacG-1	0.02227	0.06471	0.05444	1
SPV_0428	lacE-1	0.02728	0.11501	0.09865	1
SPV_0429, SPV_0430	trkH, trkA	0.58664	-0.13824	-0.71186	1
SPV_0431	unknown	0.15054	0.05768	-0.08120	1
SPV_0432	mtsA	0.11331	0.01480	-0.08612	1
SPV_0433	unknown	-0.05947	0.29641	0.36973	1
SPV_0434	mtsB	0.14951	0.07275	-0.05823	1
SPV_0435	mtsC	0.14255	0.03691	-0.08856	1
SPV_0436	cspR	0.15446	0.06780	-0.07622	1
SPV_0438	unknown	-0.10839	-0.01015	0.11607	1
SPV_0439	unknown	-0.02984	-0.13167	-0.09279	1
SPV_0440	unknown	-0.02933	-0.01177	0.03434	1
SPV_0441	rpoE	-1.83291	-0.99776	0.85037	1
SPV_0442	pyrG	0.34006	-0.01387	-0.33841	1
SPV_0443	nptA	0.02284	-0.40834	-0.42017	1
SPV_0444	endoD	0.15320	0.13504	-0.00568	1
SPV_0445	pgk	-0.81264	-0.38398	0.44499	1
SPV_0446	unknown	0.05472	0.24296	0.20510	1
SPV_0447, SPV_0448	glnR, glnA	0.00704	0.36223	0.36884	1
SPV_0449	unknown	0.14123	0.07323	-0.05400	1
SPV_0452	creX	-0.02768	-0.07400	-0.03408	1
SPV_0453	unknown	0.06503	0.08365	0.03369	1
SPV_0455, SPV_0454, SPV_2170	hsdR, hsdM, hsdS-F	-0.08873	0.05944	0.16494	1
SPV_0456	unknown	0.06306	-0.07847	-0.12931	1
SPV_0457	unknown	0.04632	0.07274	0.04300	1
SPV_0458, SPV_0460, SPV_0459, SPV_0461, SPV_2171	hrcA, dnaK, grpE, dnaJ, unknown	-0.11540	-0.21046	-0.07891	1
SPV_0463, SPV_0462	unknown, unknown	0.13936	0.03654	-0.09253	1
SPV_0464, SPV_0465	ecsA, ecsB	0.10041	0.05241	-0.03143	1
SPV_0466	blpT	-0.02233	0.11618	0.15940	1
SPV_0467, SPV_0468, SPV_0469	blpS, blpR, blpH	-0.04846	0.01196	0.06803	1
SPV_0470, SPV_0471, SPV_0472	blpC, blpB, blpA	0.21683	0.01138	-0.19202	1
SPV_0475, SPV_2176, SPV_0474, SPV_0473	pncP, pncW, blpZ, blpY	0.01410	0.11327	0.11524	1
SPV_0482, SPV_0480, SPV_0478, SPV_0483, SPV_0481, SPV_0479	infB, unknown, rimP, rbfA, unknown, nusA	-1.27275	-0.16381	1.12372	1
SPV_0484	unknown	0.10492	0.02840	-0.05774	1
SPV_0485	unknown	-0.07502	0.08955	0.17929	1
SPV_0486	unknown	-0.02167	-0.02499	0.01411	1
SPV_0487, SPV_0488	unknown, unknown	0.00428	-0.03228	-0.02034	1
SPV_0489	unknown	-0.14977	0.12619	0.29077	1
SPV_0490	unknown	-0.03522	-0.09135	-0.04469	1
SPV_0493	unknown	-2.59525	-1.14465	1.46753	1
SPV_0494	valS	-2.36302	-1.10772	1.26657	1
SPV_0496, SPV_0495	unknown, unknown	-2.55889	-2.12430	0.45166	1
SPV_0501	bglG	0.00549	0.10682	0.11507	1
SPV_0502	bglF	0.18066	0.04900	-0.11933	1
SPV_0503	bglA-2	0.04343	0.09364	0.05806	1
SPV_0504, SPV_0505	pheS, paiA	-1.54581	-0.72243	0.83476	1
SPV_0507	unknown	0.02884	0.10551	0.08713	1
SPV_0508	unknown	-0.18913	0.08887	0.28955	1
SPV_0509	higA	0.05167	0.04235	0.00923	1
SPV_0510, SPV_0511	metE, metF	0.11407	0.00544	-0.09685	1
SPV_0512, SPV_0516, SPV_0513, SPV_0517, SPV_0514, SPV_0515	pnp, mrnC, cysE, unknown, unknown, cysS	-1.75566	-0.77501	0.99155	1
SPV_0523, SPV_0522, SPV_0521	vex3, vex2, vex1	0.04216	0.08388	0.05705	1

SPV_0525, SPV_0524	vncS, vncR	0.04919	-0.05010	-0.07849	1
SPV_0527	unknown	0.22916	0.03902	-0.17091	1
SPV_0531, SPV_0529, SPV_0528, SPV_0530	glnQ2, glnP2a, glnP2b, glnH2	0.07257	0.08752	0.02677	1
SPV_0532	recJ	0.08371	-0.01361	-0.08565	1
SPV_0533	rnjB	-1.58751	-0.37082	1.22999	1
SPV_0534	estA	0.00561	0.14064	0.14673	1
SPV_0535, SPV_0536	murM, murN	0.11470	0.37268	0.27268	1
SPV_0537	unknown	0.14950	0.08780	-0.04606	1
SPV_0539, SPV_0538	unknown, uvrC	0.03502	0.00132	-0.01996	1
SPV_0540	unknown	0.21167	0.01556	-0.18551	1
SPV_0542, SPV_0541	pepV, nrd	-0.21149	-0.21147	0.01532	1
SPV_0543	unknown	-0.07820	-0.01726	0.07432	1
SPV_0546	brnQ	0.05477	0.10710	0.06652	1
SPV_0548, SPV_0547, SPV_0549	unknown, unknown, ldcB	0.03469	0.14425	0.12221	1
SPV_0550, SPV_0551	rplK, rplA	-2.34347	-1.18926	1.17160	1
SPV_0552	unknown	0.03475	0.07458	0.05252	1
SPV_0553	unknown	-0.02051	0.03486	0.06677	1
SPV_0554	unknown	0.13617	0.04512	-0.07718	1
SPV_0555	unknown	0.28349	0.10408	-0.16479	1
SPV_0556	unknown	0.09684	0.08425	0.00148	1
SPV_0557	unknown	0.05433	0.14968	0.10862	1
SPV_0558	prtA	0.46555	0.47523	0.02781	1
SPV_0561, SPV_0560, SPV_0562, SPV_0559	unknown, unknown, bgaA, unknown	0.05537	0.07374	0.03530	1
SPV_0563	unknown	0.02600	0.10879	0.09399	1
SPV_0564	unknown	-0.02262	0.28166	0.31775	1
SPV_0567	ytqB	-0.00334	0.10975	0.12758	1
SPV_0568	unknown	0.06939	0.15578	0.10056	1
SPV_0569	nha2	0.16565	0.05754	-0.09109	1
SPV_0570	yihY	-0.00415	0.05909	0.07240	1
SPV_0573, SPV_0572, SPV_0571	msrAB2, unknown, ccdA-1	-0.00427	-0.00571	0.01597	1
SPV_0575, SPV_0574	yesM, yesN	0.05653	0.04247	-0.00180	1
SPV_0576	ywlG	0.13193	0.03114	-0.08667	1
SPV_0577	zmpB	0.08700	0.01374	-0.05394	1
SPV_0578	pabB	0.00672	0.04391	0.05157	1
SPV_0579	cbpL	0.08114	0.05154	-0.01505	1
SPV_0580	gki	-0.16347	0.04778	0.22525	1
SPV_0581	thyA	-1.10597	-0.33562	0.78719	1
SPV_0582	unknown	0.08123	0.01658	-0.06244	1
SPV_0585, SPV_0584, SPV_0586, SPV_0583, SPV_0587	unknown, hflX, rnz, miaA, unknown	-0.30867	-0.77679	-0.45339	1
SPV_0588	mtaR	0.04281	0.11922	0.09343	1
SPV_0590, SPV_0589	unknown, unknown	-0.09307	0.12528	0.23074	1
SPV_0591	unknown	-0.07786	0.13795	0.22839	1
SPV_0592	rsuA-2	0.34401	0.03819	-0.28953	1
SPV_0594, SPV_0593	unknown, typA	0.09221	-0.28433	-0.36155	1
SPV_0597, SPV_0596, SPV_0595	unknown, unknown, unknown	0.03128	0.01968	0.00304	1
SPV_0599, SPV_0598	murG, murD	-5.39552	-4.34685	1.06701	1
SPV_0600	divIB	-0.21389	0.55813	0.78886	1
SPV_0601	unknown	-0.09653	0.29296	0.39998	1
SPV_0602	unknown	0.07468	0.36131	0.30360	1
SPV_0605	unknown	0.11142	-0.01486	-0.11797	1
SPV_0606	unknown	-0.06722	0.14665	0.22570	1
SPV_0607	unknown	0.18093	0.10768	-0.05818	1
SPV_0608, SPV_0610, SPV_0609	pyrF, unknown, pyrE	-0.08780	-0.02167	0.08092	1
SPV_0610	unknown	0.33599	0.06150	-0.25533	1
SPV_0611	unknown	0.07579	0.18567	0.12537	1
SPV_0612	unknown	0.10579	0.12533	0.03151	1
SPV_0613	unknown	-0.11827	-0.12781	-0.00151	1
SPV_0614	unknown	0.09822	-0.02576	-0.10535	1
SPV_0616, SPV_0618, SPV_0615, SPV_0617	glnQ3, glnP3a, glnH3, glnP3b	0.08684	-0.02191	-0.09264	1
SPV_0619	unknown	-0.09685	0.09363	0.19988	1
SPV_0620	lysS	-2.05287	-1.06378	1.00437	1
SPV_0621	lctO	0.04107	-0.07397	-0.10284	1
SPV_0623	thiM-1	-0.03917	0.11266	0.16890	1
SPV_0624	thiE-1	-0.15252	0.11510	0.28019	1

SPV_0632	thiD	0.09987	0.11622	0.02933	1
SPV_0634, SPV_0635, SPV_0633	cupA, ctpA, copY	0.17704	-0.00759	-0.17090	1
SPV_0636	spxB	0.13725	-0.10783	-0.23082	1
SPV_0637	unknown	-0.06813	0.11425	0.19371	1
SPV_0640	unknown	0.14222	-0.01585	-0.14203	1
SPV_0641	manA	-0.12257	0.04116	0.18020	1
SPV_0642	unknown	0.10912	-0.01592	-0.11247	1
SPV_0643	unknown	-0.03928	0.08981	0.14159	1
SPV_0644, SPV_0645	unknown, unknown	0.20532	0.12372	-0.05982	1
SPV_0646	unknown	0.15450	0.10223	-0.03612	1
SPV_0647, SPV_0648	unknown, comEB	0.06314	0.08524	0.03380	1
SPV_0649	upp	-0.74002	-0.65872	0.09543	1
SPV_0650	clpP	-0.18293	-0.90231	-0.70382	1
SPV_0651	unknown	0.35451	0.14163	-0.19238	1
SPV_0652	livJ	0.29225	0.06582	-0.20932	1
SPV_0653	livH	0.11417	0.05342	-0.04117	1
SPV_0654	livM	-0.07490	0.06079	0.15792	1
SPV_0655	livG	0.06357	0.06176	0.01413	1
SPV_0656	livF	0.28733	0.06701	-0.19988	1
SPV_0657	unknown	-0.11202	-0.03024	0.09411	1
SPV_0659, SPV_0660, SPV_2217	ftsE, ftsX, NA	-1.76081	-1.88719	-0.10769	1
SPV_0661	malT	0.11175	0.03133	-0.06420	1
SPV_0662	unknown	0.08929	0.10076	0.02406	1
SPV_0663	yqfR	-0.91709	-0.45739	0.47140	1
SPV_0666, SPV_0665	holA, pyrDa	0.12448	-0.00490	-0.11475	1
SPV_0667	sodA	0.06261	-0.11073	-0.15097	1
SPV_0668	unknown	-0.05124	0.13916	0.20143	1
SPV_0669	rlmN	0.20592	0.02629	-0.16566	1
SPV_0670	unknown	0.19043	0.01256	-0.15848	1
SPV_0671	unknown	0.03263	0.07941	0.06032	1
SPV_0672	ppiA	0.10383	0.13405	0.04116	1
SPV_0674, SPV_0675	rpsP, khpA	-3.24441	-2.82587	0.43315	1
SPV_0678	rimM	-0.13056	0.07339	0.21974	1
SPV_0679	trmD	-0.11560	0.06344	0.19265	1
SPV_0680	unknown	0.34877	0.24287	-0.09037	1
SPV_0681	unknown	-0.08939	-0.09688	0.00511	1
SPV_0682	unknown	0.20807	0.06665	-0.12506	1
SPV_0683	unknown	0.26518	0.07058	-0.18280	1
SPV_0684	bioY	0.18998	-0.06564	-0.23907	1
SPV_0685	gor	0.04774	-0.00719	-0.04165	1
SPV_0686, SPV_0687, SPV_0688	unknown, unknown, unknown	0.28582	0.08643	-0.18710	1
SPV_0689	metG	-2.66448	-1.43362	1.24792	1
SPV_0690	unknown	0.23304	0.06851	-0.14863	1
SPV_0691	unknown	-0.01946	-0.06703	-0.03237	1
SPV_0692	unknown	0.36476	0.03633	-0.31220	1
SPV_0693	unknown	-0.13141	0.11159	0.25242	1
SPV_0695, SPV_0694	fabG2, unknown	-0.02913	0.03030	0.07393	1
SPV_0696	unknown	0.15133	-0.00477	-0.13915	1
SPV_0697	unknown	0.05887	-0.11854	-0.16305	1
SPV_0698	unknown	0.03254	0.02858	0.00724	1
SPV_0699	unknown	-0.08544	-0.05888	0.03914	1
SPV_0700	pepN	-0.20406	-0.05833	0.16336	1
SPV_0701, SPV_0702	ciaR, ciaH	0.02323	0.13885	0.12548	1
SPV_0703	unknown	-0.10194	0.38352	0.49521	1
SPV_0704	unknown	0.09922	0.04377	-0.04050	1
SPV_0705	yoaA	0.15721	0.11192	-0.03019	1
SPV_0706	rodA	0.12079	0.03367	-0.07480	1
SPV_0707	thiJ	0.18952	0.07192	-0.09985	1
SPV_0708	unknown	-2.88000	-1.50163	1.38738	1
SPV_0709	gyrB	-0.31422	-0.10979	0.21904	1
SPV_0710	ezrA	-1.83525	-1.84176	0.00687	1
SPV_0713	unknown	-0.42602	0.62595	1.06588	1
SPV_0714	unknown	0.16113	0.15351	0.00629	1
SPV_0715	unknown	0.12257	0.08312	-0.02485	1
SPV_0717	clpE	0.17211	0.07195	-0.08545	1
SPV_0718	unknown	0.04626	-0.33313	-0.36763	1
SPV_0721, SPV_0720, SPV_0719	folD, glnQ4, glnP4	-0.32877	0.08487	0.42760	1
SPV_0722	nnrD	-0.05890	0.06694	0.13910	1

SPV_0723, SPV_0725, SPV_0726, SPV_0724	rpiA, unknown, unknown, deoB	0.01175	-0.10204	-0.10059	1
SPV_0729	unknown	0.10897	0.07969	-0.01556	1
SPV_0730	deoD	-0.04179	0.06694	0.12451	1
SPV_0731	flaR	0.22505	0.04508	-0.16939	1
SPV_0732	rpsT	0.07220	0.02382	-0.02981	1
SPV_0733	coaA	-0.17535	0.13294	0.32120	1
SPV_0734	unknown	0.16115	0.03356	-0.11586	1
SPV_0735	unknown	-0.10449	-0.01511	0.10139	1
SPV_0736	pdp	0.23669	0.05825	-0.16661	1
SPV_0738, SPV_0739, SPV_0737	cdd-1, unknown, deoC	0.15020	0.20298	0.07089	1
SPV_0740	unknown	0.10212	-0.06046	-0.14947	1
SPV_0741	unknown	0.18515	0.19893	0.02900	1
SPV_0742	unknown	0.07166	0.00888	-0.04988	1
SPV_0745	plsY	-0.96649	-0.62627	0.35784	1
SPV_0746	parE	-2.04661	-1.53584	0.52587	1
SPV_0747	unknown	-1.22686	-1.09338	0.14892	1
SPV_0748	parC	-1.50164	-1.54207	-0.02488	1
SPV_0750, SPV_0752, SPV_0751, SPV_0753, SPV_0749	unknown, unknown, unknown, pcp1, ilvE	-0.15324	-0.57492	-0.40614	1
SPV_0754, SPV_0755	unknown, tRNA-Tyr-1	0.12409	-0.35232	-0.46047	1
SPV_0755	tRNA-Tyr-1	-1.95250	-0.83243	1.13899	1
SPV_0756	tRNA-Gln-1	-3.64923	-2.64520	1.02242	1
SPV_0757	rpsA	-0.93129	-0.56620	0.37863	1
SPV_0758	unknown	-0.06099	0.11537	0.18612	1
SPV_0759	unknown	-2.92287	-1.71355	1.22284	1
SPV_0760	dnaX	-2.69116	-1.97302	0.73240	1
SPV_0761	unknown	-0.68513	-0.47282	0.22292	1
SPV_0764, SPV_0766, SPV_0763, SPV_0762, SPV_0765	sufS, sufB, sufD, sufC, sufE2	-0.02610	0.00058	0.04318	1
SPV_0767	pbp3	0.05063	0.27708	0.24283	1
SPV_0768	cozE	-0.74983	-0.46535	0.29760	1
SPV_0769	ssrA	-0.03976	-0.20404	-0.15386	1
SPV_0770	tRNA-Ser-1	-0.00848	0.11496	0.13379	1
SPV_0771, SPV_0772	fruR, fruB	-0.06030	0.00886	0.07858	1
SPV_0773	fruA	0.08329	0.12170	0.04721	1
SPV_0775	unknown	0.19319	0.14414	-0.03536	1
SPV_0776, SPV_0777	unknown, thiI	0.07740	0.11536	0.05398	1
SPV_0783, SPV_0782, SPV_0785, SPV_0784	unknown, unknown, unknown, unknown	0.18164	-0.04412	-0.20727	1
SPV_0785, SPV_0784	unknown, unknown	0.03158	0.04765	0.02994	1
SPV_0786, SPV_0787	argR2, pepX	0.03589	-0.01077	-0.03157	1
SPV_0788	dnaE	-0.16385	-0.03310	0.14422	1
SPV_0789, SPV_0790	pfkA, pyk	-3.73243	-3.12291	0.62504	1
SPV_0790	pyk	-2.67211	-2.71600	-0.03308	1
SPV_0792, SPV_0793	unknown, unknown	0.07522	0.05587	-0.00284	1
SPV_0794	unknown	0.09956	0.00678	-0.07504	1
SPV_0795	unknown	-0.10213	0.01632	0.13302	1
SPV_0796	unknown	0.15129	-0.06677	-0.20471	1
SPV_0797	unknown	-0.06068	0.03329	0.10237	1
SPV_0798	unknown	0.03551	0.03249	0.01321	1
SPV_0799	unknown	0.01489	-0.00109	-0.00568	1
SPV_0802	tex	0.13966	-0.05781	-0.18412	1
SPV_0803	unknown	-0.00288	0.00578	0.02591	1
SPV_0804	unknown	0.08093	0.02800	-0.04029	1
SPV_0805	unknown	0.18294	0.03274	-0.13672	1
SPV_0806	unknown	0.12169	0.13283	0.02728	1
SPV_0807	unknown	0.08520	0.07103	0.00093	1
SPV_0809	cad	0.03672	0.03630	0.01362	1
SPV_0810	unknown	0.01543	0.00706	0.00616	1
SPV_0811	speE	-0.03760	-0.11378	-0.05932	1
SPV_0812	lys1	0.26365	0.05832	-0.18898	1
SPV_0813	nspC	0.04590	-0.01755	-0.04931	1
SPV_0814	aguA	0.16035	-0.04375	-0.18902	1
SPV_0815	unknown	0.11561	0.06575	-0.03469	1
SPV_0816	unknown	0.05828	0.11309	0.07048	1
SPV_0819, SPV_0818	lspA, cmbR	0.03753	-0.05121	-0.07315	1
SPV_0820	rluD1	0.22671	-0.20005	-0.41041	1

SPV_0821	cbpE	0.27456	-0.00436	-0.26661	1
SPV_0822	proB	0.04272	0.22925	0.20241	1
SPV_0823	proA	-0.15775	0.15752	0.32756	1
SPV_0824	proC	0.36782	0.27499	-0.07184	1
SPV_0828	unknown	0.14399	1.14658	1.01869	1
SPV_0829	unknown	0.03884	0.25941	0.23556	1
SPV_0831	unknown	0.15779	0.06648	-0.07755	1
SPV_0833	trmFO	0.08251	-0.06628	-0.13582	1
SPV_0835	frr	-0.27657	-0.51670	-0.22439	1
SPV_0836	unknown	-0.09208	-0.64375	-0.53854	1
SPV_0837	unknown	0.05024	-0.04118	-0.08005	1
SPV_0838, SPV_2252, SPV_0839	phoH, unknown, unknown	-0.16770	0.17423	0.35424	1
SPV_0841	ald	-0.19006	0.07808	0.27864	1
SPV_0842	unknown	0.10706	0.07002	-0.02531	1
SPV_0847	infC	-3.11600	-2.06731	1.06371	1
SPV_0848	rpmI	-3.50099	-2.59448	0.91960	1
SPV_0849	rplT	-2.56772	-1.37239	1.20977	1
SPV_0850	gloA	0.07041	-0.03459	-0.09035	1
SPV_0852, SPV_0853, SPV_0851	pyrDb, lytB, pyrK	0.13468	-0.02542	-0.14674	1
SPV_0853	lytB	0.09781	0.04161	-0.04172	1
SPV_0854	pavA	-0.21518	-0.02126	0.20695	1
SPV_0855, SPV_0856	ybeY, dgkA	-1.12346	-0.83801	0.29996	1
SPV_0857	era	-0.61830	-0.76992	-0.14022	1
SPV_0858	mutM	-0.53367	-0.10443	0.44133	1
SPV_0859	coaE	-0.25228	-0.21225	0.05115	1
SPV_0860	pmrA	-0.04866	-0.08534	-0.02069	1
SPV_0861	secG	-1.11841	-0.67313	0.45513	1
SPV_0862	rnr	-0.16006	-0.49784	-0.32457	1
SPV_0863	smpB	-0.29194	-0.73928	-0.43727	1
SPV_0864	tehB	-0.11989	-0.14025	-0.00632	1
SPV_0865	coiA	-0.03420	0.13541	0.18220	1
SPV_0866, SPV_0867	pepF1, unknown	0.02785	0.09026	0.07896	1
SPV_0868	prsA	0.23650	0.02161	-0.20275	1
SPV_0870	gpmB2	0.04419	0.04544	0.01427	1
SPV_0871, SPV_0872	ebsC, unknown	-0.10226	0.32871	0.44564	1
SPV_0873	unknown	0.25917	-0.02222	-0.26605	1
SPV_0875, SPV_0874	unknown, glmU	-1.52365	-0.42915	1.10373	1
SPV_0876	unknown	-0.12561	-0.54356	-0.39946	1
SPV_0877	mtnN	-0.03681	-0.60151	-0.54942	1
SPV_0879	dnaQ	-0.06881	-0.03292	0.04835	1
SPV_0880	unknown	-0.68829	-0.19247	0.51327	1
SPV_0883	unknown	0.01263	-0.00520	-0.00419	1
SPV_0884	unknown	0.27587	-0.00997	-0.26589	1
SPV_0886	unknown	0.10557	0.06811	-0.02688	1
SPV_0886, SPV_0885	unknown, ccdA-2	-0.01997	0.06518	0.09964	1
SPV_0887	yfnA	0.18586	0.01010	-0.15778	1
SPV_0890	phtE	0.00705	0.06187	0.06640	1
SPV_0894	pepT	0.19613	-0.11838	-0.30432	1
SPV_0895	hemH	0.19057	0.08422	-0.09543	1
SPV_0896	mscL	0.01663	-0.02672	-0.02857	1
SPV_0897	mesH	0.01117	0.13167	0.13456	1
SPV_0898	queT	0.06332	0.02167	-0.03162	1
SPV_0899	tRNA-Thr-1	0.06109	0.03710	-0.01420	1
SPV_0901, SPV_0900	dapA, asd	-0.06391	0.11736	0.19423	1
SPV_0902	mnmE	-0.07492	-0.31228	-0.22393	1
SPV_0903	xylH	-0.12593	0.00380	0.14498	1
SPV_0904	tdk	-0.89026	-0.80697	0.09882	1
SPV_0905	bltD	-1.09815	-0.77353	0.33870	1
SPV_0906	prfA	-0.70845	-0.29180	0.43193	1
SPV_0907	hemK	-0.75701	-0.41071	0.35836	1
SPV_0908	tsaC	-1.14381	-0.41679	0.73800	1
SPV_0909	unknown	-0.73977	-0.28146	0.47044	1
SPV_0910	glyA	-0.20379	0.05880	0.27665	1
SPV_0911	unknown	-0.06733	0.34986	0.42826	1
SPV_0912	pvaA	0.01045	0.32857	0.33758	1
SPV_0913	unknown	-0.11670	0.13605	0.26692	1
SPV_0914	rlmCD	0.22663	-0.24715	-0.46038	1
SPV_0920	unknown	-0.10658	-0.01632	0.09754	1

SPV_0922, SPV_0921	unknown, ccrB	-0.01413	0.10989	0.13982	1
SPV_0923	unknown	0.01382	0.10834	0.10578	1
SPV_0924	unknown	-0.05461	0.03140	0.09779	1
SPV_0925	unknown	0.09480	0.00951	-0.07438	1
SPV_0926	unknown	0.15569	0.02638	-0.11056	1
SPV_0934	unknown	0.13367	0.01902	-0.10120	1
SPV_0935	unknown	0.10468	0.13900	0.04142	1
SPV_0939	mutR2	0.01803	0.04113	0.03522	1
SPV_0940	rffD	0.31442	-0.07627	-0.37674	1
SPV_0941	unknown	-0.05169	0.03985	0.10320	1
SPV_0943	unknown	0.17012	-0.12346	-0.27591	1
SPV_0944	unknown	-0.06947	0.09562	0.17763	1
SPV_0945	unknown	0.12197	0.16973	0.06413	1
SPV_0946	unknown	-0.03533	0.05617	0.10069	1
SPV_0947	unknown	0.10736	0.13970	0.05262	1
SPV_0948	nikS	-0.00420	-0.10373	-0.08519	1
SPV_0949	unknown	-0.03547	0.07714	0.12541	1
SPV_0950	mefE	0.02387	0.02027	0.01244	1
SPV_0951	unknown	0.10793	-0.00648	-0.09726	1
SPV_0953, SPV_0952	ppc, ftsW	-0.92804	-0.49642	0.44848	1
SPV_0955	trpY	0.12912	0.15210	0.03523	1
SPV_0956	unknown	0.36904	0.02534	-0.33019	1
SPV_0959, SPV_0957, SPV_0958	unknown, dnaG, rpoD	-2.98893	-2.21109	0.78240	1
SPV_0960	lafB	-0.66630	-0.54597	0.13290	1
SPV_0961	lafA	-0.30548	-0.12400	0.19889	1
SPV_0962	unknown	0.03022	0.21479	0.20179	1
SPV_0963	unknown	-1.02913	-0.71623	0.32553	1
SPV_0964	obgE	-0.62198	-0.43853	0.20149	1
SPV_0965	unknown	-0.62859	-0.48472	0.15217	1
SPV_0969	unknown	0.25039	-0.43712	-0.67098	1
SPV_0970	map	-0.05981	-0.71993	-0.64492	1
SPV_0973	pcrA	-0.47836	-0.47829	0.01486	1
SPV_0975	radC	-0.03091	0.11725	0.15943	1
SPV_0976, SPV_0974	rex, unknown	0.11946	0.11421	0.01533	1
SPV_0977	unknown	0.01181	0.05692	0.05355	1
SPV_0978	unknown	0.16464	-0.08615	-0.23184	1
SPV_0979	nifS	-1.19294	-0.82592	0.38215	1
SPV_0980	prs2	-1.33514	-0.78991	0.56214	1
SPV_0981	unknown	0.06071	0.06899	0.02169	1
SPV_0983, SPV_0984, SPV_0982, SPV_0985	ppnK, rluD3, unknown, eutD	-2.20726	-1.30531	0.91383	1
SPV_0986	unknown	-0.04630	0.08932	0.14695	1
SPV_0987	unknown	0.02239	0.10794	0.09939	1
SPV_0988	unknown	0.15868	0.04128	-0.10453	1
SPV_0989	rplU	-3.50302	-2.58755	0.92879	1
SPV_0990	unknown	-3.22686	-2.46750	0.77357	1
SPV_0991	rpmA	-2.93317	-1.76804	1.17882	1
SPV_0992	unknown	0.28123	0.04411	-0.22173	1
SPV_0993	unknown	-0.04051	-0.06628	-0.00768	1
SPV_0994	ribF	-1.56889	-0.99119	0.59415	1
SPV_0995	unknown	-0.04512	0.06130	0.12185	1
SPV_0996	unknown	-0.03279	-0.18003	-0.13563	1
SPV_0997	hlpA	-0.57284	-1.61427	-1.02428	1
SPV_0999, SPV_0998, SPV_1000	rggD, unknown, unknown	0.29277	-0.05962	-0.33481	1
SPV_1000	unknown	0.02394	0.06845	0.06180	1
SPV_1001, SPV_1002	ligA, pulA	-0.43769	-0.14641	0.30661	1
SPV_1003	unknown	0.12508	0.06227	-0.05039	1
SPV_1004	gapN	-0.20242	0.09035	0.30633	1
SPV_1008, SPV_1007	glgA, glgD	0.15530	-0.02581	-0.16233	1
SPV_1008, SPV_1007, SPV_1005, SPV_1006	glgA, glgD, glgB, glgC	0.11593	0.10053	-0.00174	1
SPV_1009	serB	0.09154	-0.07620	-0.14683	1
SPV_1010	unknown	0.11048	0.05553	-0.03989	1
SPV_1011	glxK	-0.02698	0.10819	0.14958	1
SPV_1012	eno	-2.43202	-1.54925	0.89347	1
SPV_1013	unknown	-0.00281	0.04479	0.06140	1
SPV_1017, SPV_1015, SPV_1016	unknown, rexB, rexA	-0.09666	-0.08992	0.01999	1
SPV_1018	zmpA	0.15568	0.04486	-0.09687	1

SPV_1020, SPV_1019	rnhB, rbgA	-0.86895	-0.30166	0.58259	1
SPV_1021	unknown	0.10876	0.08196	-0.00654	1
SPV_1023	xerS	0.00523	0.01438	0.02621	1
SPV_1024	lplA	-0.11395	0.14633	0.27287	1
SPV_1025	acoL	0.42460	-0.08153	-0.48753	1
SPV_1026	acoC	0.03217	0.13522	0.11717	1
SPV_1027	acoB	0.02570	0.12807	0.11730	1
SPV_1028	acoA	0.08813	0.00150	-0.07121	1
SPV_1029	pdrM	-0.20973	0.05838	0.27765	1
SPV_1030, SPV_1032, SPV_1031	pyrC, ung, mutX	-0.06491	-0.03216	0.04950	1
SPV_1034, SPV_1033	unknown, unknown	0.04924	0.04595	0.00964	1
SPV_1038	phtA	0.06339	-0.02192	-0.07152	1
SPV_1043	nrdF	-1.48964	-0.64390	0.85964	1
SPV_1044	lacR	-0.16257	0.06431	0.23958	1
SPV_1046	lacG-2	0.03463	0.00504	-0.01263	1
SPV_1047	lacE-2	0.15984	0.09356	-0.04984	1
SPV_1048	lacF-2	0.03758	0.03218	-0.00026	1
SPV_1049	lacT	0.07356	0.02399	-0.03714	1
SPV_1053, SPV_1052, SPV_1051, SPV_2301	lacA, lacB, lacC, lacD	0.17799	0.03976	-0.11924	1
SPV_1054	unknown	0.19008	-0.01677	-0.19291	1
SPV_1057	unknown	0.03044	0.05276	0.03334	1
SPV_1059	unknown	0.33821	0.12006	-0.19829	1
SPV_1060	lepA	0.06507	0.02102	-0.02942	1
SPV_1061	pphA	0.22491	0.02613	-0.18226	1
SPV_1062	recN	-0.45381	-0.67486	-0.20389	1
SPV_1063	ahrC	-0.33315	-0.19648	0.15490	1
SPV_1064, SPV_1065	unknown, ispA	-0.93927	0.04215	0.99303	1
SPV_1066	xseB	-0.83303	-0.01505	0.82986	1
SPV_1067	xseA	-0.69296	-0.12308	0.58175	1
SPV_1068	udk	0.19820	-0.03150	-0.20864	1
SPV_1069	unknown	-0.15656	0.14265	0.31538	1
SPV_1070	truB	-0.06465	0.03721	0.11749	1
SPV_1071	unknown	-0.01042	0.02814	0.05315	1
SPV_1072	unknown	-0.02111	-0.13450	-0.10080	1
SPV_1075	nirC	0.06338	0.04855	-0.00585	1
SPV_1076	srtA	-0.16064	-0.13247	0.04146	1
SPV_1077	gyrA	-1.69156	-0.82506	0.88077	1
SPV_1078	ldh	-0.42571	-0.06680	0.37486	1
SPV_1079	unknown	-0.02637	0.10943	0.15438	1
SPV_1080, SPV_1079	unknown, unknown	0.14462	0.02360	-0.10352	1
SPV_1081, SPV_1082, SPV_1080, SPV_1079	relE2, relB2, unknown, unknown	0.29695	-0.17364	-0.45749	1
SPV_1083, SPV_1085, SPV_1084	vicX, vicR, vicK	-2.46750	-2.02247	0.46008	1
SPV_1083, SPV_1085, SPV_1086, SPV_1084	vicX, vicR, mutY, vicK	-0.08092	0.12623	0.22266	1
SPV_1087	fhs	-1.10450	-0.13835	0.98237	1
SPV_1090, SPV_1088, SPV_1089	panT, coaB, coaC	-0.03257	0.01381	0.05834	1
SPV_1091, SPV_1093	niaX, niaR	0.10542	0.18192	0.09469	1
SPV_1092	unknown	-0.17111	-0.00652	0.17812	1
SPV_1093	niaR	0.10788	0.08236	-0.01413	1
SPV_1094	unknown	0.02295	0.02768	0.01838	1
SPV_1095	unknown	0.20152	0.05798	-0.12088	1
SPV_1096	uvrB	0.32857	-0.06391	-0.37541	1
SPV_1097	unknown	0.04921	0.04303	0.00645	1
SPV_1099, SPV_1098	glnQ5, glnHP5	-1.83679	-0.55989	1.29076	1
SPV_1100	zwf	-1.30042	-0.46080	0.85350	1
SPV_1101	ftsY	-0.05001	-0.08465	-0.01877	1
SPV_1102	unknown	0.11771	-0.45656	-0.56013	1
SPV_1103	unknown	-0.19495	-0.52000	-0.30927	1
SPV_1104	smc	-0.43910	-0.67173	-0.21541	1
SPV_1105	rnc	-0.65944	-0.34124	0.32836	1
SPV_1107, SPV_1106	guaC, tRNA-Arg-1	-0.05532	0.10846	0.17582	1
SPV_1108	unknown	-0.06659	0.08055	0.15974	1
SPV_1109	unknown	0.06327	-0.08187	-0.12754	1
SPV_1110	unknown	0.29463	0.07695	-0.20696	1
SPV_1111	unknown	0.07885	0.01640	-0.04401	1
SPV_1112	unknown	-0.02009	0.04234	0.07639	1

SPV_1113	unknown	0.33592	0.01853	-0.29804	1
SPV_1115	leuB	0.40073	-0.01420	-0.39658	1
SPV_1116	leuA	0.00601	0.00120	0.00995	1
SPV_1117	unknown	-0.01389	0.05142	0.07813	1
SPV_1118	cutC	0.06149	0.00327	-0.04317	1
SPV_1118, SPV_1119	cutC, unknown	0.42172	-0.04997	-0.45828	1
SPV_1120	topA	-0.32131	-0.54344	-0.20625	1
SPV_1121	unknown	-0.03312	0.16126	0.20125	1
SPV_1122	dprA	0.05762	0.14287	0.09380	1
SPV_1130	licD2	0.00696	0.24682	0.25948	1
SPV_1134, SPV_1131, SPV_1132, SPV_1133	pyrR, carB, carA, pyrB	-0.04836	0.04331	0.10515	1
SPV_1135	nth	0.30759	-0.00129	-0.29343	1
SPV_1136	unknown	0.03733	-0.08469	-0.10762	1
SPV_1137	unknown	0.44402	-0.02962	-0.46135	1
SPV_1139, SPV_1138	lemA, htpX	0.20624	0.04197	-0.14828	1
SPV_1140	rsmG	-0.04105	0.01127	0.06648	1
SPV_1141	uraA	-0.05985	0.01044	0.08330	1
SPV_1142	ffh	-0.40395	-0.35188	0.06393	1
SPV_1143	unknown	-0.32737	-0.38559	-0.04556	1
SPV_1144	unknown	0.19441	-0.07728	-0.25680	1
SPV_1146, SPV_1145	yidA, unknown	0.01151	0.20587	0.20697	1
SPV_1147	tRNA-Arg-2	0.03177	0.10240	0.08992	1
SPV_1148	rplS	-3.31667	-2.61883	0.70763	1
SPV_1151, SPV_1150, SPV_1149	unknown, crcB2, crcB1	0.22637	0.01320	-0.20153	1
SPV_1152	fld	-0.10258	-0.03430	0.08287	1
SPV_1153	pde2	-0.02706	0.00504	0.04301	1
SPV_1154	rpmE2	-1.47667	-1.76652	-0.27271	1
SPV_1155	efeU	0.15589	-0.04609	-0.18617	1
SPV_1156	efeB	0.00909	0.03436	0.04118	1
SPV_1158	gdhA	-0.09204	-0.05175	0.05141	1
SPV_1159	unknown	0.08162	0.07708	0.01250	1
SPV_1160	unknown	0.16331	0.02072	-0.12772	1
SPV_1162	unknown	0.07865	0.02323	-0.04311	1
SPV_1164	cdd-2	0.04130	-0.03892	-0.06746	1
SPV_1165	unknown	0.24372	-0.07283	-0.30504	1
SPV_1166	unknown	-0.02716	0.13823	0.17900	1
SPV_1167	appD	0.24911	0.02944	-0.20343	1
SPV_1168	appC	-0.14408	0.08323	0.23997	1
SPV_1169	appB	-0.07936	0.15745	0.25381	1
SPV_1173, SPV_1170, SPV_1171, SPV_1172	unknown, appA, unknown, nanE-2	0.08894	0.02863	-0.04981	1
SPV_1174	unknown	-0.03638	-0.04537	0.00402	1
SPV_1175	unknown	0.18646	0.06229	-0.11113	1
SPV_1176	unknown	0.04743	0.02737	-0.00828	1
SPV_1177	unknown	0.09717	-0.01863	-0.10034	1
SPV_1178	ptrB	0.35731	0.28212	-0.06314	1
SPV_1179	lanL	-0.20625	0.05198	0.27543	1
SPV_1182, SPV_2329, SPV_2327, SPV_1185, SPV_1180, SPV_2328	unknown, unknown, unknown, unknown, unknown, unknown	-0.05680	0.04307	0.11136	1
SPV_1186	unknown	-0.14756	0.11546	0.27633	1
SPV_1187	rplL	-0.27882	-0.23876	0.05344	1
SPV_1188	rplJ	-2.24878	-0.95140	1.30618	1
SPV_1189	unknown	-0.01874	0.07196	0.10887	1
SPV_1190	trzA	0.00939	0.25333	0.26049	1
SPV_1191	unknown	0.01522	-0.08167	-0.08855	1
SPV_1192	unknown	-0.04857	-0.00845	0.05644	1
SPV_1193	msrAB1	0.04859	-0.02208	-0.05355	1
SPV_1195, SPV_1193, SPV_2331, SPV_1194	hom, msrAB1, unknown, thrB	-0.11477	0.32095	0.45490	1
SPV_1196	mecA	0.08006	-0.21105	-0.27510	1
SPV_1205, SPV_1203, SPV_1204, SPV_1202	aroA, pheA, aroK, psr	-0.01993	0.16946	0.20312	1
SPV_1206	unknown	0.08674	0.07088	-0.00264	1
SPV_1207	tyrA	-0.04179	0.14803	0.20481	1
SPV_1208	aroC	-0.05049	0.16731	0.23327	1
SPV_1209	aroB	-0.01868	0.07252	0.10295	1
SPV_1210	aroE	-0.08134	-0.01643	0.07539	1

SPV_1211	aroD	0.03655	-0.25300	-0.27511	1
SPV_1212	ywbD	-0.06585	-0.21660	-0.13605	1
SPV_1213, SPV_1214	unknown, unknown	0.10718	0.11784	0.02013	1
SPV_1215	amy	0.05334	0.12217	0.07858	1
SPV_1218, SPV_1220, SPV_1219, SPV_1221, SPV_1222	potD, potB, potC, potA, murB	-5.68637	-5.30092	0.39547	1
SPV_1223	unknown	-0.15848	0.06175	0.23217	1
SPV_1224	budA	-0.07279	0.05959	0.14598	1
SPV_1225	unknown	0.06697	0.06965	0.01888	1
SPV_1226	unknown	0.31620	0.07960	-0.21731	1
SPV_1227	phoU2	0.11136	0.02315	-0.07210	1
SPV_1228	pstB2-2	0.03573	0.04361	0.02340	1
SPV_1229	pstB2-1	0.14882	-0.09315	-0.22691	1
SPV_1230	pstA2	-0.07326	-0.06169	0.02767	1
SPV_1231	pstC2	0.05631	0.04398	0.00380	1
SPV_1232	pstS2	-0.01547	-0.12010	-0.09169	1
SPV_1233	unknown	0.11380	0.04815	-0.04972	1
SPV_1234, SPV_1235, SPV_1236	unknown, unknown, spxA1	0.03414	0.21353	0.19207	1
SPV_1237	unknown	-0.03510	0.02860	0.07655	1
SPV_1238, SPV_1240, SPV_1239	nagD, hemN, unknown	0.03673	0.11306	0.09019	1
SPV_1241	unknown	0.06138	0.11584	0.06113	1
SPV_1242	unknown	-0.04414	0.12060	0.17579	1
SPV_1244, SPV_1243	hprK, lgt	-0.06158	0.17358	0.24987	1
SPV_1245	rpsU	-1.42669	-2.13911	-0.69625	1
SPV_1246	nagB	0.21057	-0.08185	-0.27691	1
SPV_1247	queA	-0.08749	-0.00110	0.10223	1
SPV_1248	cbpM	-0.06171	-0.00362	0.07562	1
SPV_1249, SPV_1250, SPV_1251	unknown, nadE, pncB	-0.42029	-0.46179	-0.03247	1
SPV_1252	unknown	0.19564	-0.01587	-0.19883	1
SPV_1253	unknown	0.14074	-0.11183	-0.24014	1
SPV_1254	unknown	-0.19083	0.01693	0.22046	1
SPV_1255	unknown	-0.00143	-0.00498	0.01137	1
SPV_1256	unknown	0.22316	-0.01514	-0.22285	1
SPV_1257	unknown	0.03753	0.05513	0.03241	1
SPV_1258	unknown	0.09181	-0.10360	-0.18048	1
SPV_1260, SPV_1259	unknown, unknown	0.07582	0.07364	0.00854	1
SPV_1261	unknown	0.16448	0.02630	-0.12106	1
SPV_1262	unknown	0.20332	0.08781	-0.10065	1
SPV_1263	unknown	-0.09013	0.07647	0.18104	1
SPV_1264	unknown	-0.02627	0.06006	0.09847	1
SPV_1265	unknown	0.10748	-0.03630	-0.12878	1
SPV_1266	unknown	0.23649	0.02150	-0.20050	1
SPV_1267	unknown	0.02500	0.00801	-0.00153	1
SPV_1272	unknown	0.12307	0.01242	-0.09819	1
SPV_1273	unknown	0.04185	0.07474	0.05391	1
SPV_1274	guaA	0.05672	0.42498	0.38213	1
SPV_1275	nagR	0.16017	0.09438	-0.04573	1
SPV_1276	unknown	0.12533	0.11004	-0.00023	1
SPV_1277	unknown	-0.12786	0.07656	0.21702	1
SPV_1278	cppA	0.07891	0.14006	0.08207	1
SPV_1279	unknown	0.05857	-0.02341	-0.06971	1
SPV_1280, SPV_2338	unknown, capA	0.24172	-0.04035	-0.26546	1
SPV_1284	unknown	0.03256	0.21149	0.19336	1
SPV_1285	def1	0.08338	-0.39324	-0.45970	1
SPV_1286	trmH	0.18987	-0.11357	-0.28944	1
SPV_1287	trxB	-0.69027	-0.05107	0.66404	1
SPV_1288	unknown	-0.05635	0.86069	0.93424	1
SPV_1290, SPV_1289	unknown, unknown	0.03481	-0.24035	-0.26069	1
SPV_1292, SPV_1293, SPV_1291	ogt, unknown, unknown	0.05326	-0.03432	-0.07342	1
SPV_1294, SPV_1295	unknown, unknown	0.06886	0.11155	0.05439	1
SPV_1296	pdxT	-0.12667	0.06532	0.20491	1
SPV_1297	pdxS	0.03101	0.05532	0.03733	1
SPV_1298	nox	0.23126	0.22454	0.00841	1
SPV_1299	unknown	-0.13216	0.04735	0.19305	1
SPV_1302, SPV_1301, SPV_1300	unknown, unknown, apbE	0.02837	0.08222	0.07703	1
SPV_1308	unknown	0.15336	0.02106	-0.11561	1
SPV_1309, SPV_1308	pgdA, unknown	-0.04197	-0.27668	-0.22487	1
SPV_1311	mocA	0.13756	-0.09693	-0.20565	1

SPV_1312	yfmL	-0.01440	-0.04148	-0.01382	1
SPV_1315	unknown	0.03956	-0.06923	-0.09280	1
SPV_1317	unknown	0.10560	0.10564	0.00985	1
SPV_1318	tuf	-3.54041	-2.42416	1.12932	1
SPV_1319	unknown	0.04626	0.15060	0.11788	1
SPV_1320	unknown	-0.34712	0.35454	0.71870	1
SPV_1321	mucB	0.16709	-0.03749	-0.18706	1
SPV_1322	unknown	0.15348	-0.02037	-0.15265	1
SPV_1323	unknown	-0.04403	0.08284	0.14389	1
SPV_1326	pgm	-0.05124	0.34877	0.41564	1
SPV_1327	bta	0.05005	0.97629	0.93913	1
SPV_1329, SPV_1328, SPV_1330	glnQ6, glnH6, glnP6	0.22919	0.14275	-0.08009	1
SPV_1331, SPV_1332	unknown, unknown	0.20830	0.17280	-0.02359	1
SPV_1333	unknown	0.01248	0.03797	0.04027	1
SPV_1339, SPV_1340, SPV_1338, SPV_1336, SPV_1337, SPV_1341, SPV_1335, SPV_1334	atpF, atpB, atpH, atpG, atpA, atpE, atpD, atpC	-1.57957	-0.71995	0.88247	1
SPV_1343	unknown	0.30865	0.06531	-0.23168	1
SPV_1344	unknown	0.10238	0.14104	0.05399	1
SPV_1346, SPV_1345	mltG, greA	-0.17890	0.19617	0.39218	1
SPV_1347	unknown	-0.21008	0.44177	0.66818	1
SPV_1348	unknown	-5.86888	-4.74299	1.13605	1
SPV_1349	murC	-5.81127	-4.81010	1.02559	1
SPV_1350	unknown	-6.20981	-4.90787	1.31648	1
SPV_1351	snf	0.04105	0.75264	0.72693	1
SPV_1353, SPV_1352	metB, patB2	0.35424	0.11163	-0.23219	1
SPV_1357	aliB	0.11257	-0.02245	-0.12413	1
SPV_1359	murE	-5.27234	-4.21521	1.06303	1
SPV_1360	unknown	0.10387	0.00124	-0.08849	1
SPV_1361	unknown	0.07071	0.04373	-0.01580	1
SPV_1362	unknown	0.10166	0.09589	0.01462	1
SPV_1363	ppaC	-1.23357	-0.50538	0.74664	1
SPV_1365, SPV_1364	unknown, unknown	0.00657	0.15668	0.16202	1
SPV_1367, SPV_1365, SPV_1366, SPV_1364	unknown, unknown, unknown, unknown	-0.12647	0.42472	0.56472	1
SPV_1369, SPV_1368	ssbA, rpsR	-3.19480	-2.33523	0.87242	1
SPV_1370	rpsF	-4.06394	-3.19361	0.88513	1
SPV_1371	asnC	-2.67622	-1.56722	1.12111	1
SPV_1372	unknown	-2.57125	-1.33795	1.24925	1
SPV_1375	unknown	0.18326	0.07202	-0.09397	1
SPV_1376	pclA	0.28112	0.10545	-0.16366	1
SPV_1377	mga1	0.03768	0.01815	-0.00341	1
SPV_1378	yaaA	0.09679	0.04226	-0.03786	1
SPV_1379	unknown	0.17596	-0.02418	-0.19031	1
SPV_1381, SPV_1380, SPV_1379	def2, unknown, unknown	0.05681	-0.02722	-0.06835	1
SPV_1383, SPV_1382	pacL, unknown	0.15654	0.13438	-0.00841	1
SPV_1384	mntE	-0.05591	0.05402	0.12433	1
SPV_1385, SPV_1389, SPV_1388, SPV_1387, SPV_1386	unknown, unknown, unknown, dapB, cca	-0.65494	-0.42066	0.24846	1
SPV_1390, SPV_1391, SPV_1392	glmM, unknown, disA	-1.40021	-0.14217	1.27429	1
SPV_1393	unknown	0.24999	-0.24924	-0.48407	1
SPV_1394, SPV_1396, SPV_1395	unknown, unknown, unknown	-0.00219	-0.04752	-0.03243	1
SPV_1397	aldR	0.02835	-0.03113	-0.04798	1
SPV_1398	engB	-0.92944	-0.72870	0.21467	1
SPV_1399	clpX	-2.05698	-1.05922	1.01270	1
SPV_1400	unknown	-0.24991	-0.24015	0.02370	1
SPV_1401	folA	-2.90783	-1.66179	1.26498	1
SPV_1402	dpr	-0.12751	0.05622	0.19714	1
SPV_1404, SPV_1403	tpiA, lytC	-1.41125	-1.54058	-0.11389	1
SPV_1405, SPV_1406	dnaD, metA	-2.65411	-2.48517	0.18525	1
SPV_1407	apt	-0.11899	-0.03374	0.09883	1
SPV_1408	unknown	0.01529	-0.04742	-0.07052	1
SPV_1409	msmK	0.20040	0.05205	-0.13467	1
SPV_1410	tRNA-Leu-1	-0.16259	-0.06272	0.11497	1
SPV_1411, SPV_1412	unknown, codY	0.10929	0.15392	0.05661	1
SPV_1413	cshA	-0.04024	0.18358	0.23584	1
SPV_1414	oxlT	0.02796	-0.01895	-0.03231	1
SPV_1415	merA	0.24797	0.10505	-0.12832	1

SPV_1419, SPV_1418	unknown, pepQ	0.03730	0.07376	0.05096	1
SPV_1420	unknown	0.17822	0.03346	-0.12882	1
SPV_1423, SPV_1424, SPV_1422	pdxK, truA, pdxU2	-1.57345	-0.85433	0.73295	1
SPV_1425	unknown	0.17788	-0.02910	-0.18830	1
SPV_1426	unknown	0.05651	0.10213	0.05554	1
SPV_1427	phnA	-0.03755	0.19502	0.25096	1
SPV_1429	unknown	-1.06854	0.07300	1.15596	1
SPV_1430	fer	-0.51943	0.26558	0.79739	1
SPV_1431, SPV_1432	gtrB, galE-1	0.10454	0.32947	0.24927	1
SPV_1433	unknown	0.15488	-0.00495	-0.14367	1
SPV_1434	ybgI	-0.24775	-0.05458	0.20677	1
SPV_1435	unknown	-0.09900	-0.00727	0.11280	1
SPV_1436	ctpE	0.16866	0.30528	0.16132	1
SPV_1437	plsC	-0.79442	0.15508	0.96377	1
SPV_1438	cadD	-0.15453	0.11063	0.27781	1
SPV_1439	rpsO	0.11596	0.02603	-0.07381	1
SPV_1440	unknown	0.02505	-0.01702	-0.02860	1
SPV_1441	unknown	0.16382	0.08088	-0.06990	1
SPV_1446, SPV_1445	unknown, unknown	0.08162	0.09016	0.02385	1
SPV_1447	unknown	0.09861	-0.04083	-0.12444	1
SPV_1448	unknown	-0.06448	-0.21178	-0.13901	1
SPV_1449	unknown	0.08647	0.05665	-0.01343	1
SPV_1451, SPV_1450	unknown, mntR	-0.18338	-0.21601	-0.01994	1
SPV_1454	unknown	-0.07003	-0.02257	0.06676	1
SPV_1457	dtd	0.43824	-0.03017	-0.45063	1
SPV_1458	relA	0.23061	0.00356	-0.21250	1
SPV_1459	unknown	0.03920	0.06974	0.04795	1
SPV_1460	pepO	0.34741	0.02775	-0.30480	1
SPV_1461, SPV_1463, SPV_1462	psaB, psaA, psaC	-0.47219	-0.05661	0.43200	1
SPV_1464	psaD	-0.11108	-0.05267	0.06906	1
SPV_1465	unknown	0.15652	0.11919	-0.01823	1
SPV_1466	unknown	0.04279	0.02639	0.00148	1
SPV_1467	unknown	0.01205	0.03956	0.04262	1
SPV_1468	gpmA	-1.41366	-1.00117	0.42524	1
SPV_1469	unknown	-0.01810	0.26660	0.29642	1
SPV_1474	divIVA	-1.91873	-1.13764	0.79280	1
SPV_1478	ylmE	-0.08039	0.27100	0.36686	1
SPV_1480, SPV_1479	ftsA, ftsZ	-4.61327	-3.29809	1.32948	1
SPV_1481	unknown	-0.05727	0.25225	0.32819	1
SPV_1482	unknown	-0.02435	0.50048	0.53748	1
SPV_1483	murF	-3.13062	-1.85529	1.28902	1
SPV_1485	recR	0.22931	0.40099	0.18351	1
SPV_1486	pbp2b	0.19694	-0.06353	-0.24627	1
SPV_1487	nanR	-0.01473	0.18796	0.21870	1
SPV_1488	nanK	0.03080	0.04296	0.02207	1
SPV_1490	unknown	-0.14024	0.08219	0.23097	1
SPV_1491	unknown	0.08953	0.08682	0.01440	1
SPV_1492	yjgK	0.18405	0.00395	-0.16806	1
SPV_1493	satC	-0.09218	0.01928	0.13069	1
SPV_1494	satB	-0.16981	0.16990	0.35169	1
SPV_1495	satA	-0.08596	-0.00776	0.09220	1
SPV_1496	nanP	0.26171	-0.07195	-0.32761	1
SPV_1497	nanE-1	-0.03594	0.10117	0.14137	1
SPV_1498	unknown	-0.00665	0.07787	0.09871	1
SPV_1499	nanB	-0.05308	0.02421	0.09098	1
SPV_1500	unknown	-0.03637	0.03992	0.08877	1
SPV_1501	ycjO	0.07849	0.01711	-0.04379	1
SPV_1502	unknown	-0.13242	0.10295	0.24864	1
SPV_1503	unknown	0.11064	0.07305	-0.02417	1
SPV_1504, SPV_1505	nanA, unknown	0.07299	0.00729	-0.05211	1
SPV_1506	axe1	-0.13160	0.11367	0.25782	1
SPV_1507	recG	0.23999	0.13117	-0.09415	1
SPV_1508	alr	-2.23272	-1.22075	1.02248	1
SPV_1509	acpS	-1.86491	-1.11027	0.76993	1
SPV_1510	aroF	-2.05898	-1.07787	0.99649	1
SPV_1511	aroG	-1.32412	-0.79108	0.55410	1
SPV_1512	secA	-1.64107	-0.71292	0.94829	1
SPV_1513	unknown	-0.10504	0.08741	0.20633	1

SPV_1514	unknown	0.01663	0.18907	0.18656	1
SPV_1515	unknown	0.22191	0.12134	-0.08981	1
SPV_1516	unknown	0.09478	0.05298	-0.02659	1
SPV_1517	unknown	0.24483	0.08027	-0.15110	1
SPV_1518	unknown	-0.00420	0.15289	0.16879	1
SPV_1519	engA	-1.00854	-0.48670	0.53584	1
SPV_1520	frp	-2.61686	-1.51161	1.12046	1
SPV_1521	dnaI	-3.49371	-2.27645	1.23419	1
SPV_1522, SPV_1523	dnaB, nrdR	-3.96051	-2.66387	1.31036	1
SPV_1526, SPV_1524, SPV_1525	unknown, gntR, unknown	-0.05813	0.17235	0.24750	1
SPV_1527, SPV_1528, SPV_2383	qsrB, qsrA, srf-22	0.11720	-0.01915	-0.12107	1
SPV_1529	unknown	0.19379	0.06856	-0.11013	1
SPV_1530	unknown	0.05611	0.09936	0.05891	1
SPV_1531	scrK	-0.05063	0.19467	0.26185	1
SPV_1532	scrA	-0.19659	0.05732	0.26823	1
SPV_1533	unknown	-0.16756	0.01600	0.19585	1
SPV_1534, SPV_1535	scrB, scrR	-1.23211	-1.26130	-0.01525	1
SPV_1536, SPV_1537	mvaA, mvaS	-0.68605	0.59957	1.29841	1
SPV_1538	unknown	-0.06977	0.11364	0.20064	1
SPV_1539	unknown	0.03448	0.15696	0.13550	1
SPV_1540	unknown	0.29953	0.01832	-0.26362	1
SPV_1541	unknown	0.06984	0.08362	0.02735	1
SPV_1543, SPV_1542	phpP, stkP	-0.26155	-0.31611	-0.04092	1
SPV_1544	sun	0.08298	-0.03493	-0.10281	1
SPV_1545	fmt	-0.40672	-0.12103	0.30215	1
SPV_1546	priA	-0.49589	-0.55831	-0.04978	1
SPV_1549	rny	-0.23249	-0.01121	0.23789	1
SPV_1551, SPV_1550	yefM, yoeB	-0.05207	-0.21539	-0.14705	1
SPV_1552	unknown	-0.33494	-0.22667	0.12414	1
SPV_1553	unknown	-0.10246	-0.17647	-0.05785	1
SPV_1554	rsfA	-0.36283	0.02377	0.40177	1
SPV_1557, SPV_1556, SPV_1558, SPV_1559, SPV_1561, SPV_1555, SPV_1560	nadD, yqeK, unknown, yqeH, corA2, unknown, unknown	-2.84749	-1.41461	1.44641	1
SPV_1562, SPV_1557, SPV_1556, SPV_1558, SPV_1559, SPV_1561, SPV_1555, SPV_1560	unknown, nadD, yqeK, unknown, yqeH, corA2, unknown, unknown	0.05642	-0.03475	-0.07875	1
SPV_1564	unknown	-0.04293	-0.03206	0.02287	1
SPV_1565	unknown	0.00993	0.05201	0.05630	1
SPV_1567, SPV_1566	trxA, unknown	0.21019	0.02986	-0.16670	1
SPV_1568	queF	0.15191	0.04178	-0.09442	1
SPV_1569	aqpZ	0.13376	0.07841	-0.04136	1
SPV_1570	unknown	0.07574	0.02225	-0.04241	1
SPV_1571	pepF2	0.23339	-0.00812	-0.22903	1
SPV_1572	unknown	0.12174	-0.00606	-0.11326	1
SPV_1573	prmA	0.13792	0.00575	-0.11676	1
SPV_1574	unknown	0.11519	0.08408	-0.01298	1
SPV_1575	unknown	-0.08912	0.11037	0.21340	1
SPV_1576	unknown	0.14144	0.02214	-0.10513	1
SPV_1577	hicB	0.13606	0.00571	-0.10793	1
SPV_1578, SPV_1579	unknown, unknown	0.00663	0.12804	0.13116	1
SPV_1580, SPV_2392, SPV_1581	unknown, ssrS, tRNA-Lys-1	-0.02579	0.01918	0.05919	1
SPV_1583, SPV_1582, SPV_1585, SPV_1584	unknown, sacA, unknown, unknown	0.11072	0.03775	-0.05959	1
SPV_1586	unknown	0.02659	0.00373	-0.00870	1
SPV_1587	mga2	-0.10020	0.04998	0.17028	1
SPV_1590, SPV_2393, SPV_1591, SPV_1589, SPV_1588	gls24, unknown, unknown, unknown, unknown	0.14039	-0.04741	-0.16928	1
SPV_1592	unknown	0.22115	0.01434	-0.19564	1
SPV_1593	cclA	0.17651	0.03749	-0.12022	1
SPV_1595, SPV_1594	unknown, unknown	-2.00901	-1.34155	0.68094	1
SPV_1597, SPV_1598, SPV_1600, SPV_1596, SPV_1602, SPV_1599, SPV_1601	trpB, trpF, trpD, trpA, trpE, trpC, trpG	0.06682	0.09571	0.04421	1
SPV_1603	unknown	0.21685	0.06296	-0.13466	1
SPV_1605	unknown	0.19317	-0.10771	-0.28375	1
SPV_1609, SPV_1610, SPV_1608, SPV_1606, SPV_1607	unknown, unknown, unknown, mgtC, sfuB	-0.06105	0.07768	0.14997	1

SPV_1609, SPV_1617, SPV_2396, SPV_1613, SPV_1610, SPV_1608, SPV_1606, SPV_1614, SPV_1612, SPV_1607	unknown, pfbA, unknown, galT-1, unknown, unknown, mgtC, phoU3, galE-2, sfuB	0.11932	0.06883	-0.03844	1
SPV_1618	unknown	0.01059	0.02593	0.02988	1
SPV_1620, SPV_1619	unknown, aatB	-0.77017	0.13846	0.92650	1
SPV_1621	unknown	0.11563	-0.00301	-0.10686	1
SPV_1622	unknown	-0.01318	0.15691	0.18073	1
SPV_1626	exoA	0.30553	-0.06331	-0.35498	1
SPV_1627	unknown	-0.01731	0.02431	0.05810	1
SPV_1630, SPV_1631	dpnD, dpnC	0.20555	0.11772	-0.08012	1
SPV_1632	paaI	-0.06410	0.09552	0.17041	1
SPV_1633	galT-2	-0.04071	0.01977	0.08146	1
SPV_1634	galK	0.34345	0.02230	-0.31200	1
SPV_1635	galR	0.10808	0.03415	-0.06066	1
SPV_1637, SPV_1636	nmlR, adhB	-0.12773	-0.04697	0.09930	1
SPV_1637, SPV_1636, SPV_1638	nmlR, adhB, czcD	0.20301	0.00860	-0.18232	1
SPV_1640	pnuC	0.31537	-0.00694	-0.30556	1
SPV_1643, SPV_1644, SPV_1645, SPV_1642	proV, unknown, unknown, proWX	-0.09277	0.08803	0.19433	1
SPV_1647, SPV_1646	pepA, unknown	0.20638	0.09710	-0.09078	1
SPV_1648	unknown	0.18532	-0.07528	-0.24791	1
SPV_1649, SPV_1652, SPV_1650, SPV_1651	piuB, piuA, piuC, piuD	0.15854	0.03476	-0.11028	1
SPV_1653	yidD	0.20713	0.17942	-0.01114	1
SPV_1654	rluB	0.00288	-0.00131	0.00620	1
SPV_1655	scpB	-0.09915	-0.24795	-0.13483	1
SPV_1656	scpA	-0.21367	-0.21968	0.00645	1
SPV_1657	xerD	-0.00904	-0.15759	-0.13577	1
SPV_1658, SPV_1659, SPV_1661, SPV_1660	unknown, unknown, murI, rdgB	-0.64250	0.38391	1.04175	1
SPV_1662	unknown	-0.22710	0.53106	0.77189	1
SPV_1663	treC	0.38204	-0.02501	-0.39001	1
SPV_1664	treP	-0.06361	0.05610	0.13281	1
SPV_1665	treR	0.06335	0.02514	-0.02857	1
SPV_1667	amiF	-0.85752	-1.05611	-0.18371	1
SPV_1668	amiE	-1.09217	-1.09768	0.00565	1
SPV_1669	amiD	-0.91112	-1.09690	-0.17182	1
SPV_1670	amiC	-1.23488	-0.96944	0.28015	1
SPV_1671	amiA	-1.36007	-0.80871	0.56764	1
SPV_1672	tacL	-0.09694	0.87849	0.98954	1
SPV_1673	gtfA	0.05089	0.03364	-0.00905	1
SPV_1674	unknown	-0.44694	-0.51302	-0.05122	1
SPV_1675	msmG	0.03057	0.12528	0.11302	1
SPV_1676	msmF	-0.06987	0.02830	0.11095	1
SPV_1677	msmE	0.09614	0.08701	0.00729	1
SPV_1678	agaN	0.19508	-0.11286	-0.30578	1
SPV_1679, SPV_1680	msmR, birA	0.00540	0.11412	0.12547	1
SPV_1682	tRNA-Ser-2	-2.88957	-1.81952	1.08184	1
SPV_1683, SPV_1682	tRNA-Ile-1, tRNA-Ser-2	-3.65502	-2.31561	1.33232	1
SPV_1697	tRNA-Asp-1	-3.77733	-2.59266	1.19531	1
SPV_1698	tRNA-Val-1	-0.26343	-0.06871	0.20850	1
SPV_1703	tRNA-Glu-2	-3.98688	-3.18217	0.82476	1
SPV_1704	rumA-2	0.19074	0.04833	-0.13230	1
SPV_1705	recX	0.12514	-0.00793	-0.11835	1
SPV_1706	unknown	-0.30752	0.54835	0.86848	1
SPV_1707	unknown	0.07675	0.12114	0.05682	1
SPV_1708	unknown	0.19137	0.02426	-0.15499	1
SPV_1710, SPV_1709	groES, groEL	-0.02384	-0.07090	-0.03123	1
SPV_1711	ssbB	0.03960	0.08832	0.06369	1
SPV_1712	ydfG	0.10560	0.03390	-0.05920	1
SPV_1713	unknown	-0.01243	-0.12665	-0.09937	1
SPV_1714	unknown	0.02586	0.08243	0.07333	1
SPV_1715	unknown	0.15036	0.13969	0.00732	1
SPV_1717, SPV_1718, SPV_1716	unknown, unknown, unknown	-0.25011	0.02585	0.28237	1
SPV_1719, SPV_1720	unknown, unknown	0.20280	0.04231	-0.14629	1
SPV_1725	yeeN	0.00665	-0.01945	-0.01150	1

SPV_1727, SPV_1729, SPV_1726, SPV_1728	unknown, unknown, ply, unknown	0.22536	-0.04671	-0.25555	1
SPV_1731	unknown	-0.01344	0.10427	0.13361	1
SPV_1732	unknown	-0.11214	0.08988	0.20881	1
SPV_1736	unknown	0.16802	0.07829	-0.07835	1
SPV_1737	lytA	-0.49306	-0.08943	0.41541	1
SPV_1738, SPV_1737	dinF, lytA	-0.06639	-0.02842	0.05399	1
SPV_1738, SPV_1739, SPV_1737	dinF, recA, lytA	-0.10528	0.06713	0.18309	1
SPV_1738, SPV_1739, SPV_1740, SPV_1737	dinF, recA, cinA, lytA	0.02803	0.22430	0.21143	1
SPV_1743, SPV_1741, SPV_1742	tsaE, lytR, unknown	-1.09279	0.08011	1.19142	1
SPV_1743, SPV_1744, SPV_1741, SPV_1742	tsaE, comM, lytR, unknown	-0.12000	0.08311	0.21712	1
SPV_1745	plcR	0.07332	-0.04799	-0.10865	1
SPV_1751, SPV_1755, SPV_1752, SPV_1754, SPV_1750, SPV_1746, SPV_1747, SPV_1748, SPV_2420, SPV_1749, SPV_1753	unknown, unknown, clyB, unknown, wrbA, unknown, pneA1, pneA2, unknown, lanM, unknown	0.06690	0.16544	0.11051	1
SPV_1757	ndk	0.39899	-0.15395	-0.53445	1
SPV_1758	rpoC	-2.31099	-1.24669	1.08437	1
SPV_1759	rpoB	-2.51445	-1.51141	1.01711	1
SPV_1760	tRNA-Cys-1	-2.56083	-1.89031	0.68324	1
SPV_1761	hlyX	0.14772	-0.03527	-0.16919	1
SPV_1762	endA	-0.00306	0.11916	0.13854	1
SPV_1763	epuA	-0.01191	0.02727	0.05656	1
SPV_1764	murA-2	-0.16190	0.01207	0.18617	1
SPV_1765, SPV_1767, SPV_1766	unknown, unknown, coaD	-0.02780	0.04425	0.08616	1
SPV_1768	asnA	-0.00679	0.11253	0.13125	1
SPV_1769	unknown	0.03287	0.01902	-0.00198	1
SPV_1771	yjfA	0.15126	0.04748	-0.08638	1
SPV_1772	acyP	0.27947	0.02556	-0.23792	1
SPV_1773	yidC1	-0.31962	0.26233	0.59460	1
SPV_1774	pflA	-0.06351	0.16223	0.24248	1
SPV_1775	lysA	0.35213	0.11104	-0.21813	1
SPV_1776	purR	0.02303	-0.31366	-0.32022	1
SPV_1778, SPV_1777	rmuC, yhaM	-0.05833	0.09759	0.16661	1
SPV_1780, SPV_1782, SPV_1779, SPV_1781	rpe, ksgA, unknown, rsgA	-0.81860	-0.74097	0.09569	1
SPV_1785, SPV_1784, SPV_1783	unknown, unknown, unknown	0.12712	0.05284	-0.05631	1
SPV_1786	unknown	0.16118	0.02985	-0.11903	1
SPV_1787, SPV_1788	rnmV, tatD	0.34881	-0.09160	-0.42817	1
SPV_1789	diiA	-0.06937	0.01463	0.09479	1
SPV_1792	unknown	-0.02352	0.11217	0.15130	1
SPV_1793	unknown	0.11104	-0.03841	-0.13557	1
SPV_1794	unknown	0.18411	0.01476	-0.15474	1
SPV_1795	unknown	0.21686	0.12106	-0.08224	1
SPV_1796	unknown	-0.15638	0.11207	0.28494	1
SPV_1797	ccpA	-0.44300	-0.20594	0.25332	1
SPV_1803, SPV_1800, SPV_1801, SPV_1799, SPV_1798, SPV_1802	unknown, unknown, unknown, unknown, unknown, unknown	0.06879	0.07365	0.01673	1
SPV_1819	nusG	-0.84842	-0.63227	0.22836	1
SPV_1820	secE	-1.43889	-0.92399	0.53570	1
SPV_1821	pbp2a	-0.19654	0.20394	0.41615	1
SPV_1822	rluD2	-0.07502	0.03714	0.12563	1
SPV_1823	gap	-2.02538	-1.42464	0.61488	1
SPV_1824	unknown	0.19264	0.06111	-0.11820	1
SPV_1826	nadC	-0.00967	0.09499	0.11892	1
SPV_1827, SPV_2426	unknown, unknown	0.10011	0.00894	-0.07762	1
SPV_1829	bguR	0.08520	-0.15675	-0.23195	1
SPV_1833, SPV_1831, SPV_1830, SPV_1832	bguC, bguD, bguA, bguB	0.13651	-0.03787	-0.15473	1
SPV_1834	adhE	-0.07209	0.03014	0.11560	1
SPV_1836	unknown	0.23763	0.08582	-0.13948	1
SPV_1837	unknown	-0.03126	0.12860	0.17423	1
SPV_1838	yajC	0.01986	0.11869	0.11297	1
SPV_1839, SPV_1838	ulaH, yajC	-0.19924	-0.32279	-0.10395	1
SPV_1840, SPV_1841	ulaG, ulaR	-0.09553	-0.03907	0.07128	1
SPV_1842	ulaF	0.05392	-0.08281	-0.12241	1

SPV_1843	ulaE	0.21144	0.05267	-0.14345	1
SPV_1844	ulaD	0.03011	0.14005	0.12360	1
SPV_1845	ulaC	0.09659	0.13743	0.05528	1
SPV_1846	ulaB	0.07195	0.19451	0.13902	1
SPV_1847	ulaA	-0.10054	0.06623	0.17890	1
SPV_1848	unknown	0.18407	-0.01126	-0.17931	1
SPV_1849	eloR	-0.18090	-0.97982	-0.78498	1
SPV_1850	yidC2	-0.17537	-1.41747	-1.22722	1
SPV_1851	rnpA	-2.28556	-2.04554	0.24758	1
SPV_1852	unknown	0.06354	-0.10310	-0.15121	1
SPV_1853	ackA	-0.00619	-0.38267	-0.36242	1
SPV_1854	unknown	-0.01385	0.04718	0.07559	1
SPV_1863, SPV_1861, SPV_1858, SPV_1859, SPV_1862, SPV_2427, SPV_1857, SPV_1860	comGA, comGC, comGF, comGE, comGB, unknown, comGG, comGD	0.18628	-0.06061	-0.22957	1
SPV_1864	unknown	0.27834	0.08984	-0.17284	1
SPV_1865	adh	0.18040	0.04572	-0.12211	1
SPV_1866	nagA	0.33979	-0.00543	-0.33314	1
SPV_1867	adr	0.15246	0.03715	-0.10182	1
SPV_1868	tgt	-0.08473	0.08395	0.18265	1
SPV_1869	unknown	-0.00782	0.12736	0.15093	1
SPV_1870	pcp2	0.42982	0.13629	-0.27504	1
SPV_1871, SPV_1872, SPV_1870	unknown, unknown, pcp2	0.09896	0.07024	-0.01619	1
SPV_1873	unknown	-0.07774	0.09761	0.18670	1
SPV_1874	unknown	0.11682	0.07470	-0.02124	1
SPV_1875	unknown	0.54895	-0.04259	-0.56735	1
SPV_1876	unknown	-0.01209	0.08763	0.11349	1
SPV_1877	thrC	0.19001	0.11344	-0.06866	1
SPV_1878	unknown	0.22810	0.01582	-0.19577	1
SPV_1879	tRNA-Leu-6	-1.16677	-0.88556	0.29513	1
SPV_1880, SPV_1879, SPV_1881	tRNA-Gln-2, tRNA-Leu-6, tRNA-His-1	-3.05386	-2.42215	0.64505	1
SPV_1882	tRNA-Trp-1	-0.79864	-0.05824	0.75679	1
SPV_1883	tRNA-Tyr-2	-2.36869	-1.36762	1.02232	1
SPV_1884	tRNA-Phe-2	-0.06443	0.00720	0.08984	1
SPV_1888	tRNA-Gly-4	-3.79064	-2.51530	1.28671	1
SPV_1895	unknown	-2.18938	-1.21116	0.99767	1
SPV_1896	gltX	-2.78445	-1.70051	1.10559	1
SPV_1897	pgi	-2.57631	-1.81740	0.77793	1
SPV_1899, SPV_1898	unknown, unknown	-0.15607	0.07780	0.24739	1
SPV_1900	patB	0.10935	-0.00122	-0.09155	1
SPV_1903	hexA	0.13500	0.06333	-0.05446	1
SPV_1904	argR1	-2.27630	-0.97125	1.31356	1
SPV_1905	argS	-0.44012	-0.17346	0.27896	1
SPV_1906	unknown	-0.02029	0.03731	0.07251	1
SPV_1907	unknown	0.07698	-0.09798	-0.15779	1
SPV_1909, SPV_1908	pnpS, pnpR	0.07510	0.02450	-0.03729	1
SPV_1910	pstS1	0.04403	0.03169	0.00295	1
SPV_1911	pstC1	0.06178	0.17932	0.13188	1
SPV_1912	pstA1	-0.00188	-0.04323	-0.02850	1
SPV_1913	pstB1	0.18310	0.10021	-0.06379	1
SPV_1914	phoU1	0.29148	0.02389	-0.25488	1
SPV_1915	unknown	0.29384	0.10065	-0.17492	1
SPV_1916	unknown	-0.06059	-0.09402	-0.02241	1
SPV_1917	unknown	-0.43470	-0.23017	0.21909	1
SPV_1918, SPV_1919	gpsA, galU	-0.19177	-0.33598	-0.13013	1
SPV_1920	unknown	-0.01720	0.09460	0.13008	1
SPV_1921	unknown	0.03358	0.04750	0.02785	1
SPV_1922	hipO	-1.11030	-0.45389	0.66732	1
SPV_1923	dapD	-0.02738	0.10776	0.15700	1
SPV_1924	unknown	0.06118	0.12306	0.07639	1
SPV_1925	pbp1b	0.07473	-0.16377	-0.22379	1
SPV_1926	tyrS	0.06535	0.03195	-0.02161	1
SPV_1927, SPV_1928	ctpC, unknown	0.06638	0.09200	0.03996	1
SPV_1929	rrmA	-0.08995	-0.01404	0.08459	1
SPV_1930	unknown	0.16706	0.05003	-0.10210	1
SPV_1931	unknown	0.11993	0.10027	-0.00354	1
SPV_1933, SPV_1932	malQ, malP	0.06394	-0.00866	-0.05970	1

SPV_1934	malX	-0.02696	0.03099	0.07843	1
SPV_1935	malC	0.09473	0.02644	-0.06170	1
SPV_1936	malD	0.04886	-0.02008	-0.05333	1
SPV_1938, SPV_1937	malR, malA	-0.18344	-0.01212	0.18659	1
SPV_1943	unknown	-0.02037	0.01458	0.04774	1
SPV_1944	unknown	0.13131	-0.03996	-0.15675	1
SPV_1945	unknown	0.13765	-0.02411	-0.13743	1
SPV_1946	unknown	0.10131	0.04233	-0.03642	1
SPV_1947	unknown	-0.19281	0.14625	0.35084	1
SPV_1948	unknown	-0.18895	0.04429	0.24557	1
SPV_1950	hisS	-2.07638	-1.24266	0.84664	1
SPV_1951	unknown	0.07791	0.06614	0.00325	1
SPV_1952	rgg	0.16638	-0.01051	-0.15294	1
SPV_1953	unknown	-0.11453	-0.03681	0.08991	1
SPV_1954	unknown	0.00341	0.03905	0.04779	1
SPV_1955	unknown	0.39446	0.11586	-0.26101	1
SPV_1956	ilvD	0.17668	0.05793	-0.10545	1
SPV_1957	tktC	0.29936	0.14594	-0.13789	1
SPV_1958	tktN	0.01526	0.02985	0.02941	1
SPV_1959	ulaA2	-0.06122	0.03127	0.10554	1
SPV_1960	ulaB2	0.07449	0.04400	-0.01642	1
SPV_1961	ulaR2	0.22255	0.09740	-0.10698	1
SPV_1962, SPV_2438	unknown, unknown	0.09741	0.09590	0.01266	1
SPV_1963, SPV_1964	rpmF, rpmG3	-2.47969	-1.38806	1.10554	1
SPV_1965	cbpN	0.40956	-0.00701	-0.39380	1
SPV_1972, SPV_1970, SPV_1969, SPV_2439, SPV_1971	unknown, unknown, unknown, unknown, unknown	0.24905	0.02902	-0.20366	1
SPV_1973	unknown	0.14405	0.07986	-0.04886	1
SPV_1975, SPV_2442	arcA, srf-26	-0.03865	0.09446	0.14280	1
SPV_1976	arcB	0.13393	0.04811	-0.07909	1
SPV_1977	arcC	0.21196	0.05357	-0.13971	1
SPV_1978	arcD	-0.09414	0.04325	0.16743	1
SPV_1979	unknown	0.02732	0.07391	0.06132	1
SPV_1981	unknown	0.14608	0.13216	-0.00005	1
SPV_1983	unknown	0.04847	-0.02724	-0.06603	1
SPV_1984	ybbK	0.09932	-0.00011	-0.08170	1
SPV_1988, SPV_1992, SPV_1987, SPV_1989, SPV_1986, SPV_1990, SPV_1995, SPV_1994, SPV_1993, SPV_1985, SPV_1991	fucY, unknown, fucL, unknown, fucI, unknown, fucK, fucA, fucU, adh2, unknown	0.09993	0.03244	-0.05720	1
SPV_1996	fucR	0.01039	0.05291	0.05503	1
SPV_1997, SPV_1999, SPV_2000, SPV_1998	adcA, adcC, adcR, adcB	-0.29931	-0.69201	-0.37921	1
SPV_2002	dltD	0.15433	0.02841	-0.11070	1
SPV_2003	dltC	0.04035	0.02063	-0.00517	1
SPV_2004	dltB	-0.03127	0.12333	0.16663	1
SPV_2005	dltA	0.04549	0.00937	-0.02770	1
SPV_2006	unknown	0.33243	-0.02132	-0.33157	1
SPV_2007	unknown	-0.02688	0.00490	0.04584	1
SPV_2008	unknown	-0.13840	0.05105	0.20253	1
SPV_2009	unknown	-0.05194	0.12141	0.18313	1
SPV_2010, SPV_2011, SPV_2012, SPV_2013	unknown, glpF, glpO, glpK	-0.05305	-0.01773	0.04676	1
SPV_2014, SPV_2010, SPV_2011, SPV_2012, SPV_2013	unknown, unknown, glpF, glpO, glpK	-0.01083	0.15049	0.17370	1
SPV_2015, SPV_2016	hslO, unknown	0.00239	-0.27782	-0.26415	1
SPV_2018, SPV_2017	unknown, cbpA	-0.04136	0.07482	0.12625	1
SPV_2019	unknown	-0.01379	-0.04818	-0.01911	1
SPV_2020	unknown	0.00067	0.06461	0.07978	1
SPV_2021	unknown	0.35805	-0.14530	-0.48439	1
SPV_2023, SPV_2022	ctsR, clpC	-0.06724	-0.14198	-0.05750	1
SPV_2024, SPV_2026, SPV_2027, SPV_2025	thiZ, thiX, unknown, thiY	0.08261	-0.01970	-0.09214	1
SPV_2029	unknown	-0.93675	0.11434	1.06519	1
SPV_2030	dnaC	-2.57753	-1.44103	1.15683	1
SPV_2031, SPV_2032	rplI, pde1	-3.09541	-1.89475	1.21655	1
SPV_2033	hpf	-0.13842	0.03345	0.18634	1
SPV_2034, SPV_2035	comFC, comFA	0.26729	0.07673	-0.17609	1

SPV_2036	unknown	-0.15466	0.01005	0.17559	1
SPV_2037	cysK	-0.01795	0.08093	0.11674	1
SPV_2039	unknown	-0.01066	0.04654	0.07382	1
SPV_2040	unknown	0.02233	0.08305	0.07637	1
SPV_2041	tsf	-2.94743	-1.93990	1.02471	1
SPV_2042, SPV_2458	rpsB, srf-30	-3.63920	-2.48122	1.17186	1
SPV_2043	pcsB	-5.03540	-3.38934	1.65658	1
SPV_2044	mreD	0.19792	-0.02291	-0.20643	1
SPV_2045	mreC	0.22616	0.13504	-0.07948	1
SPV_2046	cbiQ	0.31649	-0.07969	-0.37873	1
SPV_2047	cbiO2	0.04266	-0.05320	-0.08426	1
SPV_2048	cbiO1	-0.05028	-0.23861	-0.18545	1
SPV_2050, SPV_2051, SPV_2052, SPV_2049	rodZ, unknown, unknown, pgsA	-0.04013	-0.05908	-0.00475	1
SPV_2054, SPV_2053	recF, unknown	-0.19850	-0.07077	0.14476	1
SPV_2055	guaB	0.15961	0.04325	-0.10219	1
SPV_2056	trpS	-1.92911	-1.33385	0.61146	1
SPV_2057	unknown	-0.12206	0.02092	0.15334	1
SPV_2058	unknown	0.20918	0.02280	-0.17795	1
SPV_2059	unknown	0.06504	0.08513	0.03153	1
SPV_2060	pipR	0.21712	0.14190	-0.06162	1
SPV_2061	tRNA-Asn-2	-0.48057	-0.21273	0.28423	1
SPV_2062, SPV_2064, SPV_2063, SPV_2065	tRNA-Glu-5, comD, comE, comC1	0.16162	0.07673	-0.06911	1
SPV_2067, SPV_2066	rlmH, tRNA-Arg-5	0.18414	0.01026	-0.15560	1
SPV_2068, SPV_2069	htrA, parB	0.04014	0.06462	0.04105	1
SPV_2069	parB	0.11099	0.09725	-0.00059	1
SPV_2070	unknown	-0.22590	0.04244	0.28542	1
SPV_2073	unknown	0.02070	-0.03789	-0.04004	1
SPV_2076	unknown	0.06413	0.03773	-0.01273	1
SPV_2078	ccnC	-0.15049	0.28481	0.44213	1
SPV_2078, SPV_0024	ccnC, purA	0.17133	0.05362	-0.10440	1
SPV_2078, SPV_0024, SPV_0023	ccnC, purA, comW	-0.06784	0.06249	0.14556	1
SPV_2081	srf-01	0.09793	-0.04613	-0.12603	1
SPV_2082	unknown	-0.08222	-0.04431	0.05335	1
SPV_2084	srf-02	0.14186	0.06336	-0.06391	1
SPV_2085	unknown	-0.09444	0.09976	0.21156	1
SPV_2086, SPV_0049, SPV_0050	srf-03, comA, comB	0.06014	-0.02277	-0.06171	1
SPV_2087	unknown	-0.03076	0.12536	0.16910	1
SPV_2088	unknown	0.09442	0.01091	-0.06647	1
SPV_2089	unknown	0.09878	0.08000	-0.00515	1
SPV_2090	unknown	0.01679	0.00571	-0.00212	1
SPV_2091	unknown	0.09522	0.05333	-0.02363	1
SPV_2092	unknown	0.25479	0.06769	-0.17952	1
SPV_2096	unknown	0.08898	-0.01190	-0.08330	1
SPV_2100	unknown	0.32325	0.02447	-0.28537	1
SPV_2102, SPV_0120, SPV_0123, SPV_0119, SPV_2103, SPV_0122, SPV_0121, SPV_0124	unknown, unknown, unknown, unknown, unknown, unknown, unknown, unknown	0.05579	0.02536	-0.01356	1
SPV_2104	unknown	0.11393	0.03358	-0.06559	1
SPV_2109, SPV_0132	cibC, cibB	-0.01738	0.04863	0.07962	1
SPV_2109, SPV_0132, SPV_0133	cibC, cibB, cibA	-0.02959	0.03555	0.07713	1
SPV_2110	unknown	0.07543	-0.04947	-0.10894	1
SPV_2113	unknown	0.01046	-0.01066	-0.00367	1
SPV_2115	unknown	0.36538	0.01880	-0.32801	1
SPV_2116	unknown	-0.01002	0.20557	0.22847	1
SPV_2117	unknown	-0.23119	0.03450	0.27623	1
SPV_2119	srf-05	0.21693	0.01212	-0.19357	1
SPV_2120	srf-06	-0.21151	0.08862	0.31203	1
SPV_2121, SPV_0186	unknown, unknown	0.05898	-0.19872	-0.24939	1
SPV_2125	ccnE	0.19576	0.14442	-0.03573	1
SPV_2126	unknown	-0.01703	0.06420	0.09582	1
SPV_2127	unknown	-0.13101	0.10743	0.25480	1
SPV_2129	ccnA	0.11379	0.07206	-0.02396	1
SPV_2130	ccnB	-0.09168	0.09752	0.20218	1
SPV_2131	srf-07	0.18018	0.02570	-0.13826	1
SPV_2132	unknown	-0.00682	0.04314	0.06023	1
SPV_2133	ccnD	0.15606	0.04434	-0.09697	1

SPV_2134	unknown	-0.00759	0.02250	0.04081	1
SPV_2135	unknown	0.22801	0.03817	-0.17475	1
SPV_2137	unknown	0.30642	0.06304	-0.22676	1
SPV_2139	srf-08	-0.05177	0.14059	0.20396	1
SPV_2140, SPV_0288	NA, unknown	0.47023	0.00983	-0.44583	1
SPV_2141	unknown	-0.05983	0.18740	0.26787	1
SPV_2142	unknown	0.01729	0.12654	0.12166	1
SPV_2144	unknown	-0.31089	-0.46096	-0.13244	1
SPV_2146	unknown	-0.12872	-0.41871	-0.27058	1
SPV_2157, SPV_0391	ydiL, briC	0.12631	0.03198	-0.07579	1
SPV_2158	unknown	-0.01904	0.12660	0.16194	1
SPV_2160	unknown	0.11136	0.14701	0.04960	1
SPV_2163	unknown	0.08046	0.09937	0.03515	1
SPV_2164	unknown	0.05399	0.16174	0.12173	1
SPV_2165	unknown	0.12553	0.03926	-0.07027	1
SPV_2166	unknown	-0.25476	0.29274	0.62094	1
SPV_2167	srf-09	-0.30198	-0.11629	0.20408	1
SPV_2168	unknown	0.02902	0.01595	0.00124	1
SPV_2169	unknown	0.11780	-0.00820	-0.11898	1
SPV_2178	unknown	-0.11391	-0.08321	0.04178	1
SPV_2185	srf-10	0.14744	0.02712	-0.11042	1
SPV_2185, SPV_0500	srf-10, unknown	-0.08696	0.08043	0.18345	1
SPV_2188	unknown	0.32508	0.01678	-0.29846	1
SPV_2189	unknown	0.12218	0.04154	-0.06549	1
SPV_2192	unknown	0.13540	0.06458	-0.05633	1
SPV_2193	unknown	0.25019	0.14053	-0.09444	1
SPV_2200	srf-11	0.13383	0.05368	-0.07054	1
SPV_2201	unknown	-0.11498	-0.01446	0.11287	1
SPV_2202	unknown	0.20744	-0.00563	-0.19797	1
SPV_2203	unknown	0.08902	0.00836	-0.06253	1
SPV_2205	unknown	-0.15212	0.05158	0.21898	1
SPV_2213	unknown	-0.32036	-0.24201	0.09191	1
SPV_2218, SPV_0673	unknown, unknown	-0.27957	0.09509	0.38346	1
SPV_2219	unknown	-0.03395	0.04129	0.08573	1
SPV_2220	unknown	-0.13951	-0.16811	-0.01389	1
SPV_2226	srf-12	0.01968	0.03104	0.02492	1
SPV_2227	unknown	0.04859	0.00696	-0.02834	1
SPV_2238, SPV_0781, SPV_0778	unknown, unknown, unknown	0.01896	0.02621	0.01900	1
SPV_2239	unknown	-0.01309	-0.04284	-0.01754	1
SPV_2240	unknown	0.23059	0.03251	-0.17656	1
SPV_2242	unknown	0.17666	0.00592	-0.15849	1
SPV_2244	unknown	0.16528	0.03098	-0.12133	1
SPV_2245	unknown	0.10478	0.07166	-0.01755	1
SPV_2246	unknown	0.15533	0.08495	-0.06155	1
SPV_2247	srf-13	0.16417	-0.05166	-0.20696	1
SPV_2249, SPV_0817	unknown, unknown	0.20216	0.03022	-0.15790	1
SPV_2250	unknown	-0.19150	0.06191	0.26765	1
SPV_2251	unknown	-0.06796	0.08097	0.16160	1
SPV_2257, SPV_0846, SPV_0844, SPV_2256, SPV_0843	unknown, unknown, comEC, unknown, comEA	-0.00787	0.04696	0.05431	1
SPV_2257, SPV_0846, SPV_2256	unknown, unknown, unknown	0.03433	0.07888	0.06371	1
SPV_2258	srf-14	-3.71015	-2.60907	1.11507	1
SPV_2259	rpmG1	-0.05300	-0.08555	-0.01647	1
SPV_2260	unknown	0.09863	0.00626	-0.07374	1
SPV_2265, SPV_0888	phtD, lmb	-0.06977	0.13178	0.21412	1
SPV_2266	dhaM	0.30949	0.01262	-0.28219	1
SPV_2267	unknown	-0.08881	0.01783	0.11955	1
SPV_2270	srf-15	-3.81885	-2.68678	1.14270	1
SPV_2274	unknown	0.21205	-0.05805	-0.25621	1
SPV_2278	unknown	0.09432	0.07577	-0.00180	1
SPV_2279	unknown	0.07908	0.01963	-0.04131	1
SPV_2282	shp	0.13889	0.13934	0.01568	1
SPV_2283	unknown	0.22420	0.06034	-0.14943	1
SPV_2285, SPV_0931, SPV_0930	unknown, pezT, pezA	-0.49415	0.31523	0.82404	1
SPV_2287, SPV_2285, SPV_0931, SPV_2286, SPV_0930	unknown, unknown, pezT, unknown, pezA	0.37259	-0.01259	-0.36652	1
SPV_2287, SPV_2285, SPV_0931, SPV_2286, SPV_0930, SPV_0927	unknown, unknown, pezT, unknown, pezA, nplT	0.14491	0.07698	-0.05206	1

SPV_2288, SPV_0918, SPV_0919, SPV_0917, SPV_0915, SPV_0916	unknown, piaD, unknown, piaC, piaA, piaB	0.21826	0.09242	-0.11515	1
SPV_2289	unknown	-0.03383	0.03877	0.08634	1
SPV_2290	unknown	0.00461	0.06967	0.08031	1
SPV_2291	srf-16	-0.14460	0.03787	0.19306	1
SPV_2292	srf-17	0.11703	0.04265	-0.06579	1
SPV_2293	phtF	0.18354	0.05667	-0.11006	1
SPV_2297	phtB	0.30780	0.02589	-0.26963	1
SPV_2298	unknown	0.06931	0.02402	-0.03085	1
SPV_2299	unknown	0.09629	-0.00168	-0.08420	1
SPV_2304	unknown	0.20171	0.01814	-0.18142	1
SPV_2309	unknown	0.00371	0.07994	0.08357	1
SPV_2310	unknown	0.22823	0.01384	-0.20042	1
SPV_2311	unknown	0.09261	-0.04083	-0.12043	1
SPV_2314	unknown	-0.11357	-0.06839	0.05911	1
SPV_2315	unknown	0.35807	-0.03087	-0.36704	1
SPV_2317	srf-19	0.11392	0.15730	0.05804	1
SPV_2318	unknown	0.41899	0.06856	-0.33994	1
SPV_2319	unknown	0.24129	-0.01516	-0.24273	1
SPV_2320	unknown	0.01647	-0.01530	-0.01708	1
SPV_2325	unknown	0.37519	0.00464	-0.35456	1
SPV_2326	unknown	0.27851	0.01493	-0.24900	1
SPV_2332	unknown	0.26767	-0.08778	-0.34138	1
SPV_2333	unknown	-0.17010	-0.06793	0.11508	1
SPV_2337	unknown	0.12716	0.06418	-0.04703	1
SPV_2340, SPV_1309, SPV_1308	unknown, pgdA, unknown	0.13633	-0.02680	-0.15177	1
SPV_2349	unknown	0.08464	0.04162	-0.02754	1
SPV_2351	msbA	-0.04609	0.12670	0.18217	1
SPV_2362	unknown	0.14666	0.01543	-0.11578	1
SPV_2363	unknown	0.19989	0.01847	-0.16462	1
SPV_2364	unknown	0.10335	0.17071	0.08115	1
SPV_2365, SPV_1444	unknown, thrS	-3.21358	-1.92598	1.30251	1
SPV_2366	unknown	0.10714	0.01981	-0.07336	1
SPV_2367	unknown	-0.08402	0.05141	0.14569	1
SPV_2370, SPV_2369	unknown, unknown	0.07655	0.13915	0.08321	1
SPV_2371	unknown	0.06062	0.15096	0.10521	1
SPV_2372	unknown	-0.02925	-0.02607	0.01655	1
SPV_2373	unknown	0.10515	0.08012	-0.01442	1
SPV_2377	unknown	0.07676	0.03218	-0.03218	1
SPV_2378	srf-21	0.35745	0.10559	-0.23527	1
SPV_2381	unknown	0.22952	-0.05543	-0.27164	1
SPV_2382	unknown	0.15725	0.00427	-0.13453	1
SPV_2384	unknown	0.39623	-0.03628	-0.41466	1
SPV_2388	unknown	-0.13421	-0.01897	0.12821	1
SPV_2389	unknown	0.41194	-0.14951	-0.53090	1
SPV_2391	hicA	0.33831	0.04532	-0.27682	1
SPV_2392, SPV_1581	ssrS, tRNA-Lys-1	-0.01924	0.16425	0.19663	1
SPV_2395	unknown	0.27336	-0.00112	-0.26068	1
SPV_2398, SPV_2397	unknown, unknown	0.06350	0.05465	0.00476	1
SPV_2407	unknown	0.18801	0.03832	-0.13670	1
SPV_2408	unknown	-0.05812	0.07026	0.14008	1
SPV_2411	unknown	0.20903	0.10270	-0.09438	1
SPV_2414	unknown	0.07109	-0.01547	-0.07541	1
SPV_2416	unknown	0.03991	-0.02222	-0.04806	1
SPV_2417	unknown	0.24016	0.04085	-0.17644	1
SPV_2418	unknown	-0.01027	0.10436	0.12611	1
SPV_2419	unknown	0.12982	0.11386	-0.00460	1
SPV_2422	unknown	-0.01620	-0.11903	-0.09272	1
SPV_2423	rpmG2	-1.60897	-1.06610	0.56021	1
SPV_2432	unknown	0.00505	-0.00743	0.00216	1
SPV_2433, SPV_1902	srf-24, patA	0.23631	-0.01811	-0.23973	1
SPV_2434	unknown	0.27611	-0.00455	-0.25248	1
SPV_2436	srf-25	0.14635	-0.01037	-0.14397	1
SPV_2437	unknown	-0.12156	0.01325	0.14387	1
SPV_2441, SPV_1974	unknown, unknown	0.10864	-0.01171	-0.09778	1
SPV_2446	unknown	0.30421	0.04522	-0.24504	1
SPV_2448	unknown	-0.05683	0.09947	0.17221	1
SPV_2450	unknown	-0.09339	-0.00072	0.10465	1

SPV_2453	unknown	-0.02334	-0.08015	-0.04197	1
SPV_2454, SPV_2028	srf-29, cbpD	0.23700	0.00137	-0.22093	1
SPV_2455	unknown	0.13076	0.10157	-0.01370	1

Supplementary References

1. van Raaphorst, R., Kjos, M. & Veening, J.-W. Chromosome segregation drives division site selection inStreptococcus pneumoniae. *Proc. Natl. Acad. Sci. U. S. A.* **114**, E5959–E5968 (2017).
2. Keller, L. E. *et al.* Three New Integration Vectors and Fluorescent Proteins for Use in the Opportunistic Human Pathogen Streptococcus pneumoniae. *Genes (Basel).* **10**, 394 (2019).
3. Liu, X. *et al.* High-throughput CRISPRi phenotyping identifies new essential genes inStreptococcus pneumoniae. *Mol. Syst. Biol.* **13**, 931 (2017).
4. Gibson, D. G. *et al.* Enzymatic assembly of DNA molecules up to several hundred kilobases. *Nat. Methods* **6**, 343–345 (2009).
5. Sorg, R. A., Kuipers, O. P. & Veening, J.-W. Gene Expression Platform for Synthetic Biology in the Human Pathogen *Streptococcus pneumoniae*. *ACS Synth. Biol.* **4**, 228–239 (2015).
6. Engler, C., Kandzia, R. & Marillonnet, S. A one pot, one step, precision cloning method with high throughput capability. *PLoS One* **3**, e3647 (2008).
7. Mercy, C. *et al.* RocS drives chromosome segregation and nucleoid protection in Streptococcus pneumoniae. *Nat. Microbiol.* **4**, 1661–1670 (2019).
8. Hoskins, J. *et al.* Genome of the bacterium Streptococcus pneumoniae strain R6. *J. Bacteriol.* **183**, 5709–17 (2001).
9. Tettelin, H. *et al.* Complete genome sequence of a virulent isolate of Streptococcus pneumoniae. *Science* **293**, 498–506 (2001).
10. Sorg, R. A. *et al.* Collective Resistance in Microbial Communities by Intracellular Antibiotic Deactivation. *PLOS Biol.* **14**, e2000631 (2016).
11. O'Neil, P., Lovell, S., Mehzabeen, N., Battaile, K. & Biswas, I. Crystal structure of histone-like protein from Streptococcus mutans refined to 1.9 Å resolution. *Acta Crystallogr. Sect. F, Struct. Biol. Commun.* **72**, 257–62 (2016).
12. Stamsås, G. A. *et al.* CozEa and CozEb play overlapping and essential roles in controlling cell division in *Staphylococcus aureus*. *Mol. Microbiol.* **109**, 615–632 (2018).
13. Liu, X. *et al.* Exploration of Bacterial Bottlenecks and Streptococcus pneumoniae Pathogenesis by CRISPRi-Seq. *Cell Host Microbe* **29**, 107-120.e6 (2021).
14. Diebold-Durand, M. L., Bürmann, F. & Gruber, S. High-throughput allelic replacement screening in bacillus subtilis. in *Methods in Molecular Biology* vol. 2004 49–61 (Humana Press Inc., 2019).
15. Youngman, P. *et al.* Methods for genetic manipulation, cloning and functional analysis of sporulation genes in Bacillus subtilis. (1989).
16. Hassan, A. K. *et al.* Suppression of initiation defects of chromosome replication in Bacillus subtilis dnaA and oriC-deleted mutants by integration of a plasmid replicon into the chromosomes. *J. Bacteriol.* **179**, 2494–502 (1997).
17. Yansura, D. G. & Henner, D. J. Use of the Escherichia coli lac repressor and operator to control gene expression in Bacillus subtilis. *Proc. Natl. Acad. Sci. U. S. A.* **81**, 439–43 (1984).
18. Lemon, K. P., Kurtser, I. & Grossman, A. D. Effects of replication termination mutants on chromosome partitioning in Bacillus subtilis. *Proc. Natl. Acad. Sci. U. S. A.* **98**, 212–7 (2001).
19. Avery, O. T., Macleod, C. M. & McCarty, M. Studies on the chemical nature of the substance inducing transformation of pneumococcal types : induction of transformation by a desoxyribonucleic acid fraction isolated from pneumococcus type III. *J. Exp. Med.* **79**, 137–58

(1944).

20. Tettelin, H. *et al.* Complete genome sequence of a virulent isolate of Streptococcus pneumoniae. *Science (80-.).* **293**, 498–506 (2001).

21. Kjos, M. *et al.* Bright fluorescent Streptococcus pneumoniae for live-cell imaging of host-pathogen interactions. *J. Bacteriol.* **197**, 807–18 (2015).

22. Horsburgh, M. J. *et al.* sigmaB modulates virulence determinant expression and stress resistance: characterization of a functional rsbU strain derived from Staphylococcus aureus 8325-4. *J. Bacteriol.* **184**, 5457–67 (2002).

23. Perego, M., Spiegelman, G. B. & Hoch, J. A. Structure of the gene for the transition state regulator, abrB: regulator synthesis is controlled by the spo0A sporulation gene in Bacillus subtilis. *Mol. Microbiol.* **2**, 689–699 (1988).

24. Keller, L. E., Rueff, A. S., Kurushima, J. & Veening, J. W. Three new integration vectors and fluorescent proteins for use in the opportunistic human pathogen Streptococcus pneumoniae. *Genes (Basel).* **10**, (2019).

25. Hassan, A. K. *et al.* Suppression of initiation defects of chromosome replication in Bacillus subtilis dnaA and oriC-deleted mutants by integration of a plasmid replicon into the chromosomes. *J. Bacteriol.* **179**, 2494–502 (1997).

26. Ouellette, S. P. *et al.* Analysis of MreB interactors in Chlamydia reveals a RodZ homolog but fails to detect an interaction with MraY. *Front. Microbiol.* **5**, 279 (2014).

27. Monk, I. R., Tree, J. J., Howden, B. P., Stinear, T. P. & Foster, T. J. Complete Bypass of Restriction Systems for Major Staphylococcus aureus Lineages. *MBio* **6**, e00308-15 (2015).
