# Supplementary material for: CcrZ is a pneumococcal spatiotemporal cell cycle regulator that interacts with FtsZ and controls DNA replication by modulating the activity of DnaA
Source: Nat Microbiol. 2021 Aug 9;6(9):1175–87. doi: 10.1038/s41564-021-00949-1 (PMC8387234; doi:10.1038/s41564-021-00949-1)
Supplement: Supplementary file 2 — Reporting Summary [file 41564_2021_949_MOESM2_ESM.pdf]

nature research

Corresponding author(s): Jan-Willem Veening

Last updated by author(s): Jun 30, 2021

Reporting Summary

Nature Research wishes to improve the reproducibility of the work that we publish. This form provides structure for consistency and transparency in reporting. For further information on Nature Research policies, see our Editorial Policies and the Editorial Policy Checklist.

Statistics

For all statistical analyses, confirm that the following items are present in the figure legend, table legend, main text, or Methods section.

n/a	Confirmed	
☐	☒	The exact sample size (n) for each experimental group/condition, given as a discrete number and unit of measurement
☐	☒	A statement on whether measurements were taken from distinct samples or whether the same sample was measured repeatedly
☐	☒	The statistical test(s) used AND whether they are one- or two-sided<br>*Only common tests should be described solely by name; describe more complex techniques in the Methods section.*
☒	☐	A description of all covariates tested
☐	☒	A description of any assumptions or corrections, such as tests of normality and adjustment for multiple comparisons
☐	☒	A full description of the statistical parameters including central tendency (e.g. means) or other basic estimates (e.g. regression coefficient) AND variation (e.g. standard deviation) or associated estimates of uncertainty (e.g. confidence intervals)
☐	☒	For null hypothesis testing, the test statistic (e.g. F, t, r) with confidence intervals, effect sizes, degrees of freedom and P value noted<br>*Give P values as exact values whenever suitable.*
☐	☒	For Bayesian analysis, information on the choice of priors and Markov chain Monte Carlo settings
☒	☐	For hierarchical and complex designs, identification of the appropriate level for tests and full reporting of outcomes
☒	☐	Estimates of effect sizes (e.g. Cohen's d, Pearson's r), indicating how they were calculated

Our web collection on statistics for biologists contains articles on many of the points above.

Software and code

Policy information about availability of computer code

Data collection	OD measurement data where acquired using i-control 3.8.2.0 software (Tecan) or Gen5 3.04.17 (BioTek); Microscopy images were acquired using Las X 3.4.2.18368 (Leica), SoftWoRx 7.0.0 (GE Healthcare) or ZEN Blue 1.1.2.0 (Zeiss).
Data analysis	Microscopy images were deconvolved using Huygens 17.10.0p4 (SVI) and analyzed using Fiji 1.52q (ImageJ distribution; https://fiji.sc/). Microscopy signal was analyzed using Oufti (Paintdakhi, A. et al. 2016. Mol. Microbiol. 99, 767–777), MicrobeJ 5.13l (Ducret, A. et al. 2016. Nat Microbiol 1, 16077), Morphometrics (Ursell, T. et al. 2017. BMC Biol. 15, 17) and iSBatch (Caldas, V. E. A. et al. 2015. Mol. Biosyst. 11, 2699–2708). Mass spectrometry data were acquired using Xcalibur 4.0.27.19 (Thermo Fisher) and analyzed using Mascot 2.5 (Matrix Science) and Scaffold 4.4 (Proteome Software Inc). Data were analyzed and plotted using R 3.6.1.

For manuscripts utilizing custom algorithms or software that are central to the research but not yet described in published literature, software must be made available to editors and reviewers. We strongly encourage code deposition in a community repository (e.g. GitHub). See the Nature Research guidelines for submitting code & software for further information.

Data

Policy information about availability of data

All manuscripts must include a data availability statement. This statement should provide the following information, where applicable:

- Accession codes, unique identifiers, or web links for publicly available datasets
- A list of figures that have associated raw data
- A description of any restrictions on data availability

The data that support the findings of this study are available from the corresponding author upon request. Data gathered from string-db are available at https://string-db.org/cgi/network?taskId=bGMNPfTunceU&sessionId=bWgBlgywaw1D. Published crystal structure of LicA in complex with AMP is available at https://www.rcsb.org/structure/4R78. SPD_0476 (CcrZ) amino acid sequence can be found on the UniProt Knowledgebase https://www.uniprot.org/uniprot/A0A0H2ZQL5.

Genomes sequences data are available at NCBI Sequence Read Archive (SRA) under the following accession number PRJNA564501 and CRISPRi-seq data are available under accession number PRJNA740244.

Field-specific reporting

Please select the one below that is the best fit for your research. If you are not sure, read the appropriate sections before making your selection.

☒ Life sciences ☐ Behavioural & social sciences ☐ Ecological, evolutionary & environmental sciences

For a reference copy of the document with all sections, see nature.com/documents/nr-reporting-summary-flat.pdf

Life sciences study design

All studies must disclose on these points even when the disclosure is negative.

Sample size	No sample-size calculations were performed. Sample sizes were chosen to allow appropriate statistical tests and were in line with other published studies in the field.
Data exclusions	No data was excluded.
Replication	All observed effects were highly significant and always successfully replicated (at least twice).
Randomization	Not applicable. Samples were not allocated to experimental groups
Blinding	Not applicable. Samples were not allocated to experimental groups

Reporting for specific materials, systems and methods

We require information from authors about some types of materials, experimental systems and methods used in many studies. Here, indicate whether each material, system or method listed is relevant to your study. If you are not sure if a list item applies to your research, read the appropriate section before selecting a response.

Materials & experimental systems

n/a	Involved in the study	
☐	☒	Antibodies
☒	☐	Eukaryotic cell lines
☒	☐	Palaeontology and archaeology
☒	☐	Animals and other organisms
☒	☐	Human research participants
☒	☐	Clinical data
☒	☐	Dual use research of concern

Methods

n/a	Involved in the study	
☒	☐	ChIP-seq
☒	☐	Flow cytometry
☒	☐	MRI-based neuroimaging

Antibodies

Antibodies used	Polyclonal rabbit anti-GFP IgG (Invitrogen #A-6455) ;<br>Goat anti-rabbit IgG HRP-conjugated (Abcam AB205718) ;<br>Serum anti-serotype 2 from rabbit (Neufeld antisera, Statens Serum Institut 16745) ;<br>Goat anti-rabbit IgG coupled to Alexa Fluor 555 (Invitrogen #A27039)<br>Anti-CcrZ IgG from rabbit serum
Validation	All four commercial antibodies (#A-6455, #A27039, 16745, AB205718) were purchased from providers who have validated the antibodies for the use of Western blot and/or immunostaining .<br>Anti-CcrZ IgG were validated in the present study by Western blot with purified S. pneumoniae CcrZ.
