# Supplementary material for: CcrZ is a pneumococcal spatiotemporal cell cycle regulator that interacts with FtsZ and controls DNA replication by modulating the activity of DnaA
Source: Nat Microbiol. 2021 Aug 9;6(9):1175–87. doi: 10.1038/s41564-021-00949-1 (PMC8387234; doi:10.1038/s41564-021-00949-1)
Supplement: Source Data Fig. 3 — Unprocessed membranes and gel images for Fig. 3c,d. [file 41564_2021_949_MOESM15_ESM.pdf]

Source Data Figure 3. Uncropped western blot membranes used in Figure 3c and uncropped gel used in Figure 3d

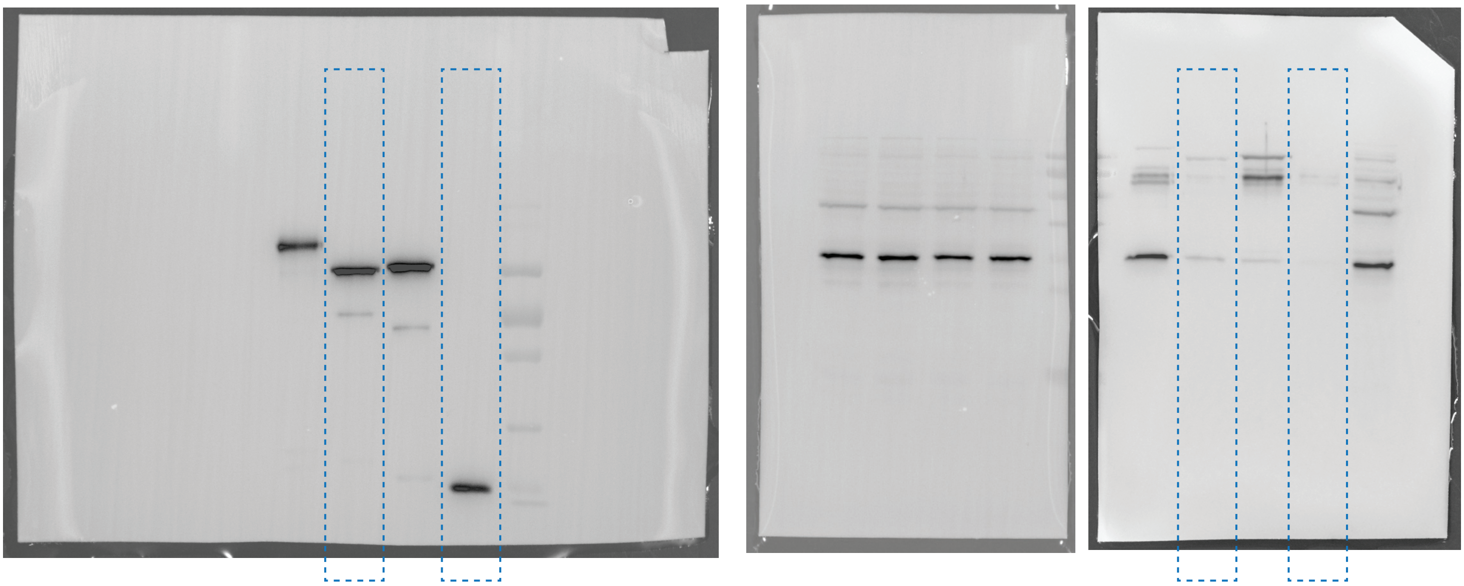

Fig. 3C left

Fig. 3C right

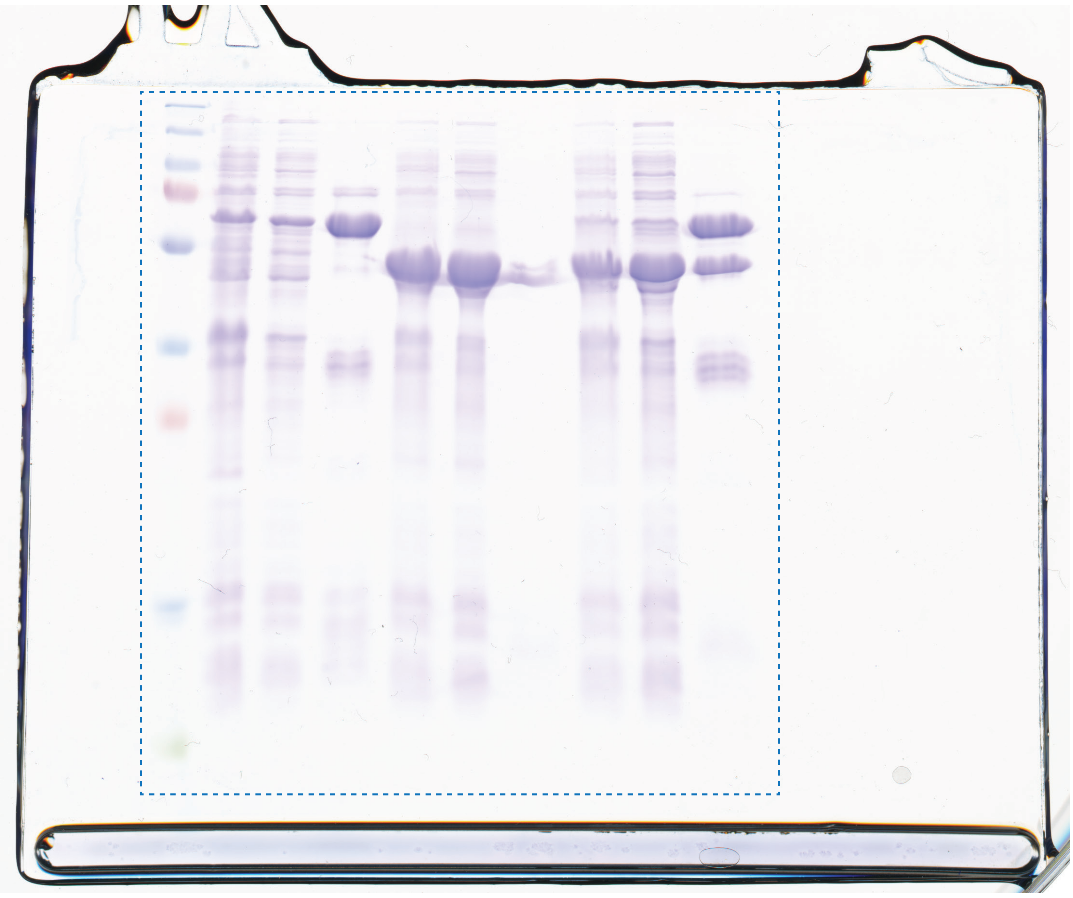

Fig. 3d
