# Supplementary material for: CcrZ is a pneumococcal spatiotemporal cell cycle regulator that interacts with FtsZ and controls DNA replication by modulating the activity of DnaA
Source: Nat Microbiol. 2021 Aug 9;6(9):1175–87. doi: 10.1038/s41564-021-00949-1 (PMC8387234; doi:10.1038/s41564-021-00949-1)
Supplement: Source Data Extended Data Fig. 1 — Unprocessed membranes images for Extended Data Fig. 1c. [file 41564_2021_949_MOESM20_ESM.pdf]

Source Data Extended Data Figure 1. Uncropped western blot membranes used in Extended Data Figure 1c

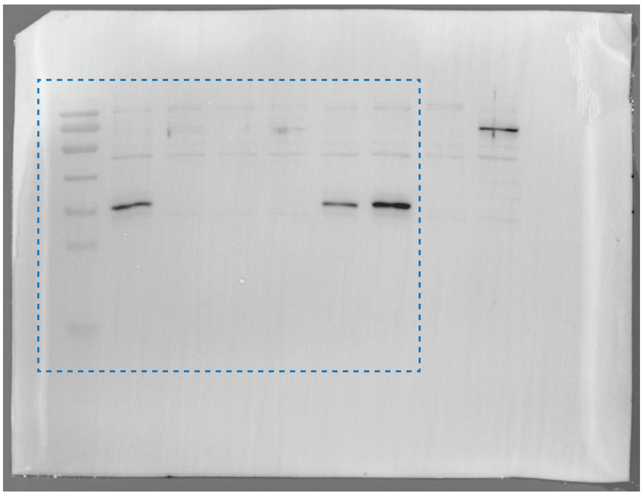

ED Fig. 1c left

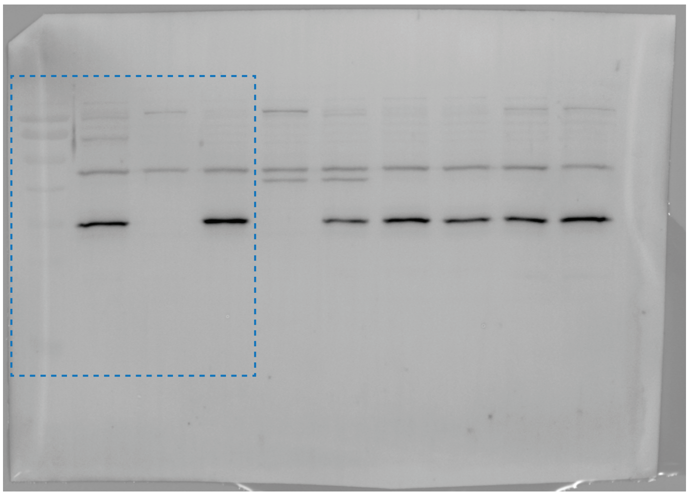

ED Fig. 1c middle left

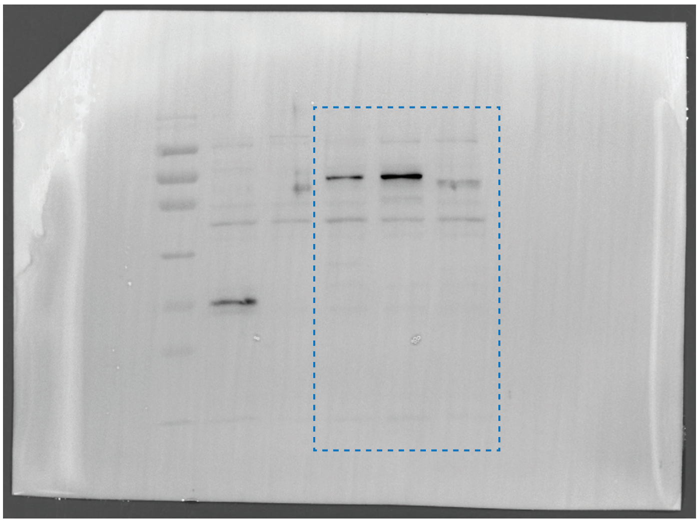

ED Fig. 1c middle right

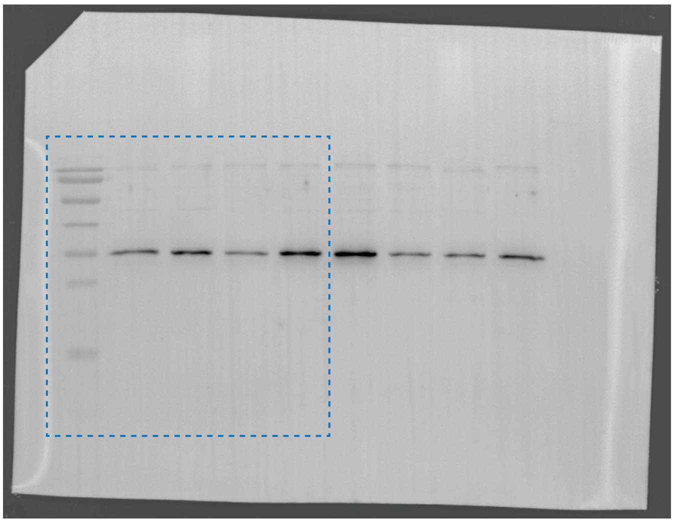

ED Fig. 1c right
